# Shallow whole-genome sequencing of plasma cell-free DNA accurately differentiates small from non-small cell lung carcinoma

**DOI:** 10.1186/s13073-020-00735-4

**Published:** 2020-04-21

**Authors:** Lennart Raman, Malaïka Van der Linden, Kim Van der Eecken, Karim Vermaelen, Ingel Demedts, Veerle Surmont, Ulrike Himpe, Franceska Dedeurwaerdere, Liesbeth Ferdinande, Yolande Lievens, Kathleen Claes, Björn Menten, Jo Van Dorpe

**Affiliations:** 1 Department of Pathology, Ghent University Hospital, Ghent University, Corneel Heymanslaan 10, 9000 Ghent, Belgium; 2 Center for Medical Genetics, Department of Biomolecular Medicine, Ghent University Hospital, Ghent University, Corneel Heymanslaan 10, 9000 Ghent, Belgium; 3 Department of Respiratory Medicine, Ghent University Hospital, Ghent University, Corneel Heymanslaan 10, 9000 Ghent, Belgium; 4 Department of Respiratory Medicine, AZ Delta, Deltalaan 1, 8800 Roeselare, Belgium; 5 Deparment of Pathology, AZ Delta, Deltalaan 1, 8800 Roeselare, Belgium; 6 Department of Radiation Oncology, Ghent University Hospital, Ghent University, Corneel Heymanslaan 10, 9000 Ghent, Belgium

**Keywords:** Lung cancer, Liquid biopsy, Cell-free DNA, Shallow whole-genome sequencing, Copy number alterations, Histological classification

## Abstract

**Background:**

Accurate lung cancer classification is crucial to guide therapeutic decisions. However, histological subtyping by pathologists requires tumor tissue—a necessity that is often intrinsically associated with procedural difficulties. The analysis of circulating tumor DNA present in minimal-invasive blood samples, referred to as liquid biopsies, could therefore emerge as an attractive alternative.

**Methods:**

Concerning adenocarcinoma, squamous cell carcinoma, and small cell carcinoma, our proof of concept study investigates the potential of liquid biopsy-derived copy number alterations, derived from single-end shallow whole-genome sequencing (coverage 0.1–0.5×), across 51 advanced stage lung cancer patients.

**Results:**

Genomic abnormality testing reveals anomalies in 86.3% of the liquid biopsies (16/20 for adenocarcinoma, 13/16 for squamous cell, and 15/15 for small cell carcinoma). We demonstrate that copy number profiles from formalin-fixed paraffin-embedded tumor biopsies are well represented by their liquid equivalent. This is especially valid within the small cell carcinoma group, where paired profiles have an average Pearson correlation of 0.86 (95% CI 0.79–0.93). A predictive model trained with public data, derived from 843 tissue biopsies, shows that liquid biopsies exhibit multiple deviations that reflect histological classification. Most notably, distinguishing small from non-small cell lung cancer is characterized by an area under the curve of 0.98 during receiver operating characteristic analysis. Additionally, we investigated how deeper paired-end sequencing, which will eventually become feasible for routine diagnosis, empowers tumor read enrichment by insert size filtering: for all of the 29 resequenced liquid biopsies, the tumor fraction could be increased in silico, thereby “rescuing” three out of five cases with previously undetectable alterations.

**Conclusions:**

Copy number profiling of cell-free DNA enables histological classification. Since shallow whole-genome sequencing is inexpensive and often fully operational at routine molecular laboratories, this finding has current diagnostic potential, especially for patients with lesions that are difficult to reach.

## Background

Despite research and development at unrivaled pace, lung cancer remains the most dominant cause of cancer-related deaths worldwide [1]. With time, the disease’s overall 5-year survival rate did however increase, mainly due to the expanding pool of diverse treatment [2]. In order to administer the most appropriate therapy, accurate histological classification is essential to guide individual decisions.

The subcategorization of non-small cell lung cancer (NSCLC), representing approximately 85% of all lung cancers, in inter alia adenocarcinoma (LUAD) and squamous cell carcinoma (LUSC), has long been clinically relevant, especially for targeted therapy [3]. For chemotherapy, likewise, the therapeutic agent pemetrexed, for example, proves to be effective in patients with non-squamous histology; thus, it is not recommended for treating LUSC [4]. In contrast, further subclassifying small cell lung cancer (SCLC) has fewer diagnostic consequence, as it is sufficient to correctly determine the small cell histology in order to initiate chemotherapeutic treatment [5]. Ongoing clinical trials are evaluating targeted and immunotherapies for molecularly characterized SCLCs, yet none of these are routinely implemented at present [6].

Current favored histological subtyping approaches are based on hematoxylin and eosin staining, and morphologic tissue examination, often in combination with immunohistochemistry. Notwithstanding these methodologies are rapid and affordable, they coexist with major disadvantages, inherent to the requirement of tumor biopsies. These drawbacks mainly emerge from the invasive nature of the used procedures, such as bronchoscopy, endobronchial ultrasound with transbronchial needle aspiration, or percutaneous computed tomography-guided transthoracic lung biopsy—techniques that require expertise and operator skills and, importantly, always coincide with considerable patient discomfort and sometimes serious complications. For lung cancer patients with inaccessible lesions or substantial comorbidity, tissue examination might be delayed or simply not possible [7].

Conceptionally, establishing diagnosis through tissue analysis introduces another inconvenience: since tumors are heterogenic in essence, solely a portion of the cancer complexity is examined [8]. This bias underestimates both intratumoral and intermetastatic heterogeneity.

With the above intrinsic limitations of current methods in mind, the idea of liquid biopsies (LBs), which are classic blood samples, is rapidly emerging as an interesting alternative. Cell-free plasma DNA (cfDNA), likely to contain a share of tumor-derived fragments in cancer patients, forms an attractive novel source of diagnostic information [9]. The most profound advantage of LBs is undeniably the convenience by which tumor DNA is collected, which could enable molecular pathologists to genetically track tumor evolution over time in a personalized manner.

Several specialized high-throughput techniques to analyze cfDNA have been developed. Especially, ultra-deep duplex sequencing for mutation calling appears to be a promising approach; however, it remains expensive, requires targeted panels, and is yet to be extensively validated for its clinical use [10]. Shallow (coverage 0.1–0.5×) whole-genome sequencing (sWGS), on the other hand, has been shown to reliably detect copy number alterations (CNAs) in cfDNA [11–13]. As for single nucleotide polymorphisms (SNPs), specific CNAs are widely described to correlate with diagnosis in lung cancer [14].

Since approximately 70–75% of all lung cancer cases are diagnosed as advanced stage diseases (stage III and IV), and plasma genomic abnormality increases with tumor stage, we focused on patients with advanced stage tumors during recruitment [10, 15]. For this proof of concept study, 51 LBs (20 LUADs, 16 LUSCs, and 15 SCLCs) and 39 matched formalin-fixed paraffin-embedded (FFPE) solid biopsies (SBs) have been analyzed.

## Methods

### Study population

Between January 2016 and June 2019, 51 patients diagnosed with LUAD, LUSC, or SCLC were enrolled (Additional file 1: Table S1). Classification was executed according to the 2016 World Health Organization’s guidelines. When available, results were compared with FFPE tissue (*n* = 39). SBs were mostly taken at primary diagnosis, whilst LBs were sometimes drawn shortly before starting second-line treatment (Additional file 1: Table S2). Negative controls included LBs from healthy subjects (females from routine non-invasive prenatal testing (NIPT) and healthy males; *n* = 60) and FFPE samples from benign tissue (*n* = 9). Other than these in-house cases, public segmental copy number data, derived from SNP array 6.0 (Affymetrix, Santa Clara, CA) experiments, complemented with clinical information and a list of significantly aberrant loci per histological subtype, were collected from the supplement of the study of Seidel et al., which presents the collective effort from the consortia “Clinical Lung Cancer Genome Project” (CLCGP) and “Network Genomic Medicine” (NGM) [16]. This dataset was filtered on histology (exclusively LUAD, LUSC, and SCLC; *n* = 843).

### Formalin-fixed paraffin-embedded DNA sequencing

Seven sections were cut from the FFPE tumor blocks. The middle five were subjected to DNA extraction whilst section one and seven, stained with hematoxylin and eosin, served as references to locate regions with high tumor cell concentrations. After macrodissection, DNA extraction was performed with the QIAamp DNA FFPE Tissue Kit (Qiagen, Hilden, Germany), according to the manufacturer’s instructions. DNA shearing to 200 bp fragments was executed by Covaris’ Adaptive Focused Acoustics technology using an M220 Focused-ultrasonicator (Covaris, Woburn, Massachusetts). Using 200 ng of starting material, library construction was completed by use of the NEXTflex Rapid DNA-Seq Kit and NEXTflex DNA Barcodes (Bioo Scientific, Austin, TX). After pooling, cluster generation and sequencing were executed by respectively a cBot 2 and HiSeq 3000 system (Illumina, Essex, UK). The minimal number of reads (single-end (SE); 50-cycle mode) per sample was intended to be at least 15 million (mean coverage of 0.25×).

### Cell-free DNA sequencing

Blood samples were collected in Cell-Free DNA BCT tubes (10 mL) (Streck, La Vista, NE) or PAXgene Blood ccfDNA tubes (10 mL) (PreAnalytiX, Hombrechtikon, Switzerland). Within 24 h of collection, plasma isolation was executed by one (PAXgene) or two consecutive (BCT) centrifugation steps, according to the manufacturer’s protocol. cfDNA extraction from 3.5 mL of plasma was performed using the Maxwell RSC ccfDNA Plasma Kit (Promega, Madison, WI), following the manufacturer’s instructions.

Using 25 μL (~ 12 ng) of cfDNA, library preparation was executed by a Hamilton Star liquid handler using the NEXTflex Cell Free DNA-Seq Library Prep Kit and protocol (Bioo Scientific) and NEXTflex DNA Barcodes (Bioo Scientific), initiated by magnetic bead-based size selection to enrich for 100–170 bp fragments [17]. Pooling, cluster generation, and sequencing were performed in correspondence to the SBs.

A selection of 29 LBs (Additional file 1: Table S2) was reanalyzed by paired-end (PE) sequencing, using similar steps as described above, to computationally enrich for tumor-derived reads by insert size (IS) filtering [17, 18]. We aimed at obtaining at least 80 million reads per sample, employing the Illumina NovaSeq 6000 (Illumina, Essex, UK).

### Copy number profiling

Raw reads were mapped by Bowtie 2 (v2.3.2) onto human reference genome GRCh38, using the *fast-local* flag [19]. Biobambam’s bamsormadup (v2.0.87) was used to mark duplicate reads and to sort the resulting bam files [20]. No additional quality filtering was applied. The latter files were indexed by SAMtools (v1.4.1) [21]. The novel WisecondorX (v1.1.2) was deployed to reliably deduce normalized genome-wide bin-wise (100 kb) log_2_ ratios, representing copy number [22]. Normalization was performed using two healthy reference sets: one for cfDNA (*n* = 333) and one for FFPE samples (*n* = 181). Note that these sets exclude the 60 liquid and nine solid controls used for comparative analyses in this study, to avoid normalization bias. Stretches of expected equal copy number were defined by circular binary segmentation segments [23]. Regions without information were interpreted as loci of undeterminable copy number (e.g., at centromeres).

### Aberration calling

Losses and gains were called once segments had an absolute *Z*-score of 3 or more. These scores are calculated as shown by Eq. (1) [22]:

1 $$ {Z}_{\mathrm{segment}\left(n\to m\right)}=\frac{\mu_w\left({R}_n,{R}_{\dots },{R}_m\right)-\mu \left({\mu}_w\left({r}_{1,n},{r}_{1,\dots },{r}_{1,m}\right),\dots, {\mu}_w\Big({r}_{p,n},{r}_{p,\dots },{r}_{p,m}\Big)\right)}{std\left({\mu}_w\left({r}_{1,n},{r}_{1,\dots },{r}_{1,m}\right),\dots, {\mu}_w\left({r}_{p,n},{r}_{p,\dots },{r}_{p,m}\right)\right)} $$

*Z*_segment(*n* → *m*)_ represents the *Z*-score of a segment ranging from bin *n* until *m*. *μ*_*w*_() calculates the average of a sequence of bins weighted by normal variability derived during reference creation in WisecondorX [22]. The functions *μ*() and *std*() calculate a default mean and standard deviation, respectively. *R*_*n*_ represents the ratio of the studied sample at bin *n*, whilst, for example, *r*_1, *n*_ holds the ratio of the same locus in the first reference “control sample.” There are *p* controls in the reference.

### Defining copy number tumor burden

The “plasma genomic abnormality (PGA) score” has previously been shown to correlate to clinical outcome across different cancer types [24, 25], whereas ichorCNA calculates the most likely circulating tumor DNA (ctDNA) fraction according to copy number profiles [26]. In accordance to their intention—quantifying copy number tumor burden—we developed a novel, more robust score, which enables control-case comparison in a manner that is less subject to variable Gaussian variance resulting from coverage bias and less subject to variable sample quality: the copy number profile abnormality (CPA) score. This score quantifies the deviation of segments from the normal diploid state, using segmental *Z*-scores, as shown by Eq. (2):

2 $$ \mathrm{CPA}={\sum}_{i=1}^n\left(\left|{Z}_{{\mathrm{segment}}_i}\right|\times {l}_{{\mathrm{segment}}_i}\right)/n $$

In this equation, ${Z}_{{\mathrm{segment}}_i}$ represents the *Z*-score of segment_*i*_. The length of this segment is given by ${l}_{{\mathrm{segment}}_i}$. Copy number profiles are defined by *n* segments. The CPA score is expressed per 100 Mb. More details covering this formula (Additional file 2), and a thorough comparison between CPA, PGA, and ichorCNA values (Additional file 3: Figure S1), can be found in the supplement.

### Abnormality calling

In order to detect cancerous LBs, the theoretical cumulative distribution function of the controls’ CPA scores, which is assumed to be normally distributed, was calculated. The abnormality cutoff was chosen at *P*(*x*) = 0.99, delineating a type 1 error cutoff: samples that cross this limit are abnormal at the 1% false discovery rate (FDR) level. This process was repeated for the SBs separately, as FFPE-derived profiles tend to be subject to increased levels of noise, which generally lowers *Z*-scores and thereby CPA values [27].

### Predictive modeling

The used public training set contains copy number data complemented with relevant clinical information [16]. This set holds LUAD (*n* = 424), LUSC (*n* = 351), and SCLC (*n* = 68) patients, each of which were released alongside segmental continuous copy number states derived from array experiments on tumor tissue. This dataset is sufficiently large to train a robust model, which served as an evaluation platform for our in-house SBs and LBs. For all samples (public and in-house), whilst aiming at partly sidelining variable tumor fraction as a source of variability, loci (100 kb bins) were given three states to serve as model features: loss (− 1), copy neutral (0), and gain (+ 1).

Five different classifiers (random forest; support vector machine; logistic regression with ridge, elastic net, and lasso regularization) were evaluated using leave-one-out cross-validation (LOOV) on a class-balanced training set (*n* = 204), sampled from the public data. The most accurate model, according to the mean area under the curve (mAUC), preceded by a one-versus-all receiver operating characteristic (ROC) analysis, was passed to the newly sequenced SBs and LBs. Details on all machine learning steps can be found in supplement (Additional file 2).

### Tumor enrichment by insert size filtering

As mentioned before, 29 LBs were reanalyzed by PE sequencing. Three parallel computational pipelines were implemented to derive copy number profiles: the first uses all raw PE reads, the second uses exclusively properly paired reads with an IS between 90 and 135 bp, and the third uses randomly sampled PE reads, such that the same number of reads as in the second pipeline is obtained—this to assure novel CNAs do not result from increased levels of noise caused by downsampling. Raw IS statistics were derived by Picard (v2.21.1; broadinstitute.github.io/picard/), using the *CollectInsertSizeMetrics* functionality.

## Results

**Fig. 1:**
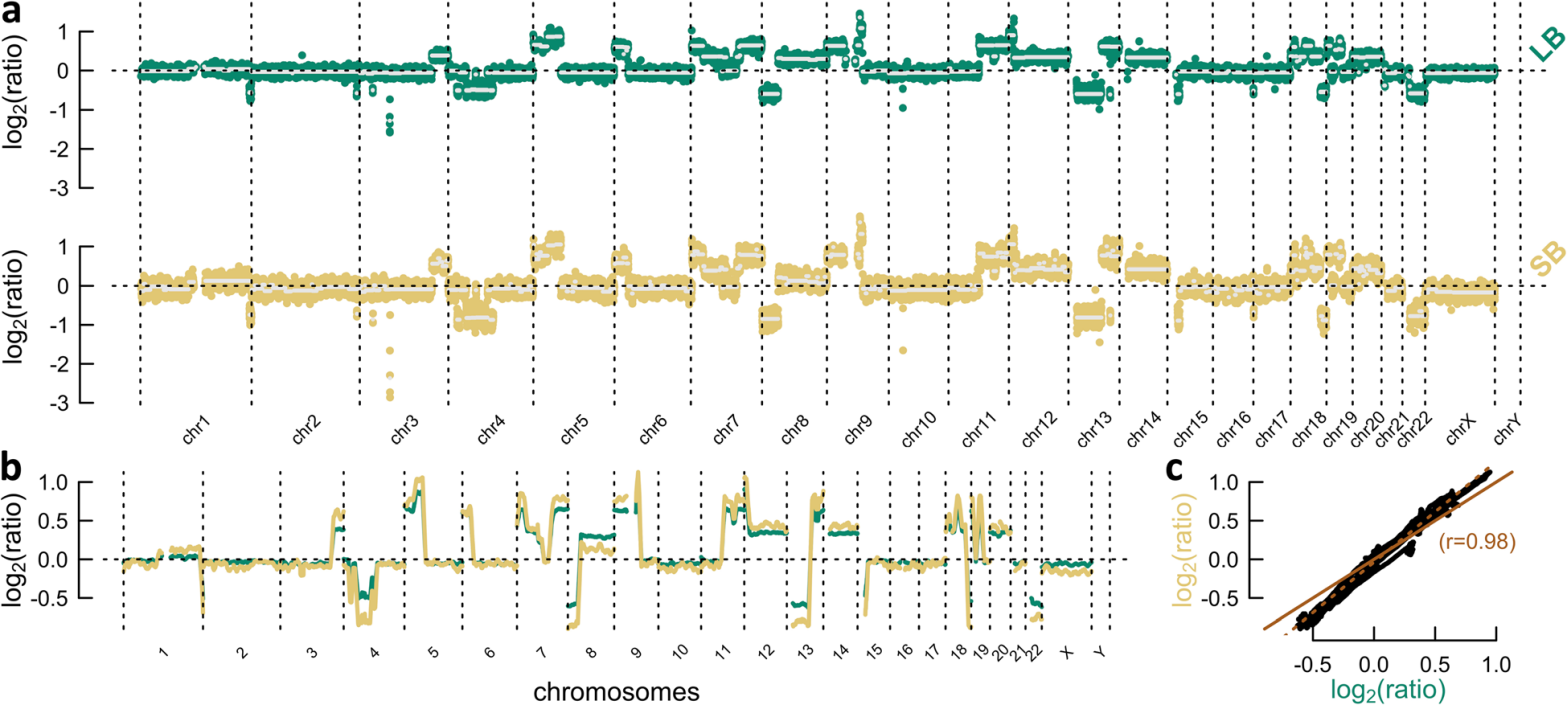
Copy number profile comparison between the liquid (LB) and solid biopsy (SB) of patient 17. **a** Copy number profile of the LB (top) and SB (bottom). Dots represent bins, whereas horizontal white lines indicate segments, covering bins of expected equal copy number. **b** Overlap plot derived from a smoothing sliding window, which interpolates the average of 100 enclosed bins to its central position. **c** Correlation scatter plot, using previous smoothened values. The solid line indicates identity (*y* = *x*), whereas the dotted line results from a total least squares analysis. A steeper dotted than solid line shows that the tumor fraction is higher in the SB. The Pearson correlation coefficient (*r*) is given

Following sample collection, sequencing, and read mapping, CNAs were inferred from 51 LBs and 39 SBs (see the “Methods” section). A copy number profile, a major concept throughout our study, tries to visualize the copy number state across the genome in a predefined number of bins (Fig. 1). Every dot in such a profile represents a bin for which copy number is inferred. Each bin is expressed as a log_2_ ratio between the observed and the expected number of reads, the latter matching the healthy diploid state. As bin-wise values are subject to Gaussian noise, segments are typically inferred, covering bins of equal copy number. It is paramount to comprehend that, for example, not every gain has the same log_2_ ratio value. This is caused by three main effects: the copy number state of the gain (3*n*, 4*n*, …), tumor heterogeneity (when not all tumor cells express the gain), and tumor fraction (samples always contain germline DNA). With these concepts in mind, following outcome could be described.

### LUAD displays less plasmatic abnormalities in comparison to LUSC and SCLC

To gain insight in the level of plasmatic abnormality, we developed a novel statistic, named the “CPA score” (see the “Methods” section). In practice, this measure can range from zero, representing a “flat” profile, to, for example, 10, matching a highly aberrant sample. This approach was found to outperform previously published methods in terms of tumor detection accuracy, such as ichorCNA and the PGA score (Additional file 3: Figure S1) [24, 26].

**Fig. 2:**
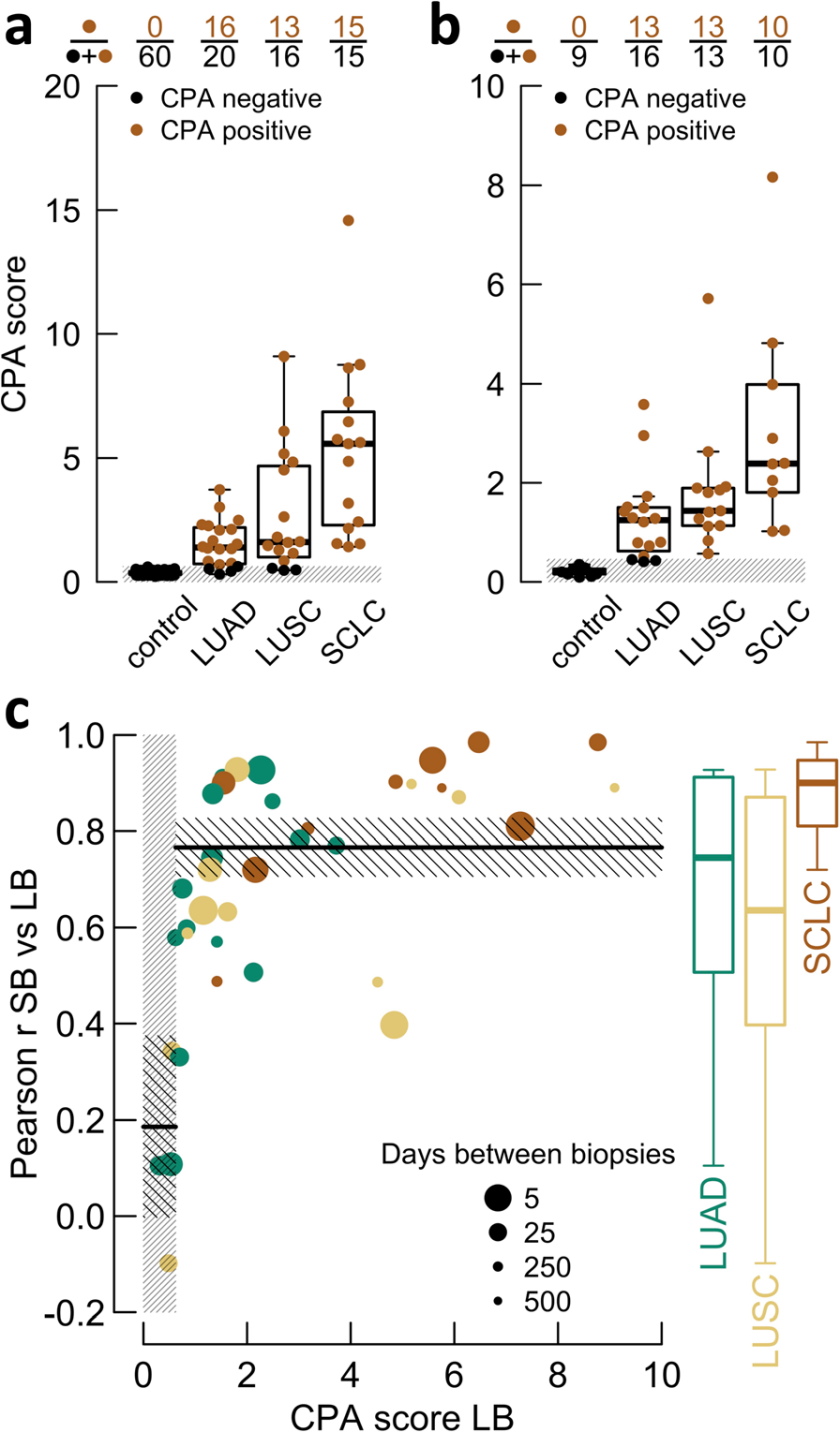
Copy number abnormality and concordance analyses across histological subtypes. **a** Dots represent the copy number profile abnormality (CPA) score for liquid biopsies (LBs). Boxplots indicate distributions. The gray box (bottom) shows the 1% false discovery rate (FDR) cutoff. Dot colors (positive/negative) clarify their position with respect to this box. The fractions on top summarize the latter. **b** Identical to **a**, yet dots represent solid biopsies (SBs). **c** Scatter plot evaluating the SB/LB Pearson correlation (*r*; defined as in Fig. 1) in relation to the CPA score of LBs. Dot size indicates days between biopsies and is quadratically scaled. The dense gray box (left) represents the 1% FDR cutoff. The horizontal solid lines are means, embedded within non-dense boxes, showing their uncertainty (95% CI). Boxplots (right) indicate the underlying distributions per histological subtype. Means, standard errors, and boxplots are weighted by dot size

The CPA score across LBs was found to significantly differ (*P* < .001; Welch’s *t* test) between lung cancer patients and healthy controls. More specifically, LUAD and LUSC were noticed with a lower plasmatic abnormality than SCLC (*P* < .001 and *P* = .014, respectively; Welch’s *t* test). Assuming normality (Lilliefors’ test returns *P* = .372 in the control group), 99% of control LBs are expected to have a CPA lower than 0.623 (see the “Methods” section), suggesting aberrations can be detected in 86.3% (44/51) of advanced stage lung cancer LBs (80% of LUADs, 81.3% of LUSCs, and 100% SCLCs) at the 1% FDR level (Fig. 2a). For the SBs, this was the case for 92.3% (36/39) of the samples (Fig. 2b). Here, a similar increase in abnormality along the sequence LUAD-LUSC-SCLC is present.

Two possible effects explain the observed differences in plasmatic abnormality between the histological subtypes. First, LUAD tumors tend to contain tumor cells with less structural aberrations, which is, as anticipated, illustrated by the CPA across SBs (Fig. 2b) [14]. Second, since a higher portion of SBs have detectable aberrations, a prominent flat profile cause must be an insufficient ctDNA fraction, noticeable in LUAD and LUSC. SCLCs, on the other hand, are known for having excessive cell turnovers (a high proliferation rate in combination with extensive apoptosis and necrosis), which consequently affects the plasmatic tumor fraction [28]. Interesting to note in this context, circulating tumor cells have been described to be excessively present in SCLC patients [29].

### Concordance between solid and liquid biopsies highly depends on plasmatic tumor fraction

When comparing the copy number profiles between paired SBs and LBs, mostly well-correlating cases were encountered. In general, LBs with a high tumor fraction often exhibit identical aberrations compared to their solid counterpart (e.g., patient 17; Fig. 1). Here, deletions and gains are positioned in the same loci, whereas their amplitudes—a concept defined as the absolute value of a segment’s log_2_ ratio—are mostly tumor fraction dependent.

Concerning less concordant cases, three main factors explain their presence. First, disregarding constitutional events, a LB without observable aberrations, presumably caused by insufficient ctDNA, cannot show agreement (e.g., patient 5; Additional file 4). Second, as SBs represent a distinct part of the total tumor, whereas LBs study all sources of cfDNA simultaneously, tumor heterogeneity introduces additional disconcordance (e.g., patient 37; at 5p and chromosome X; Additional file 4). Third, since paired SBs and LBs were sometimes taken at independent moments, another source of potential divergence is present, considering time and treatment both contribute to tumor evolution, as, for example, tumor cells resistant to first-line treatment can evolve or clonally expand to alter genomic composition. These dissimilarities were thus possibly seen when dealing with large time gaps (e.g., patient 22; at 6p; this patient was treated with concomitant chemoradiotherapy, after which progression was observed 7 months later, when palliative treatment was initiated, indicating chemoresistance; Additional file 4); however, whether these were caused by either heterogeneity or evolution could not be confirmed. Revisiting tumor heterogeneity, 19 patients were represented by aberrant LBs and SBs taken within the same period of time. Of these, four showed clear evidence of heterogeneity, whereas three others solely suggested heterogeneity (Additional file 3: Figure S2).

Notwithstanding the potential consequence of the above confounders, LBs with detectable tumor DNA (CPA > 0.623) represent their paired SB well, as indicated by a mean Pearson correlation of 0.767 (95% CI 0.706–0.827) (Fig. 2c). For specifically SCLC, this measure amounted to 0.861 (95% CI 0.790–0.931). Although often large interval times are present (e.g., patient 15 and 22, with intervals of 504 and 345 days, respectively), the correlation metric (close to 0.9) implies that tumor characteristics, according to the observed CNAs, largely remain the same at progression, confirming ineffective first-line treatment. Remark that for LBs to operate as a diagnostic tool, the described effect of interval time is evidently not relevant.

### Liquid biopsy copy number profiles correlate with public solid biopsy data

**Fig. 3:**
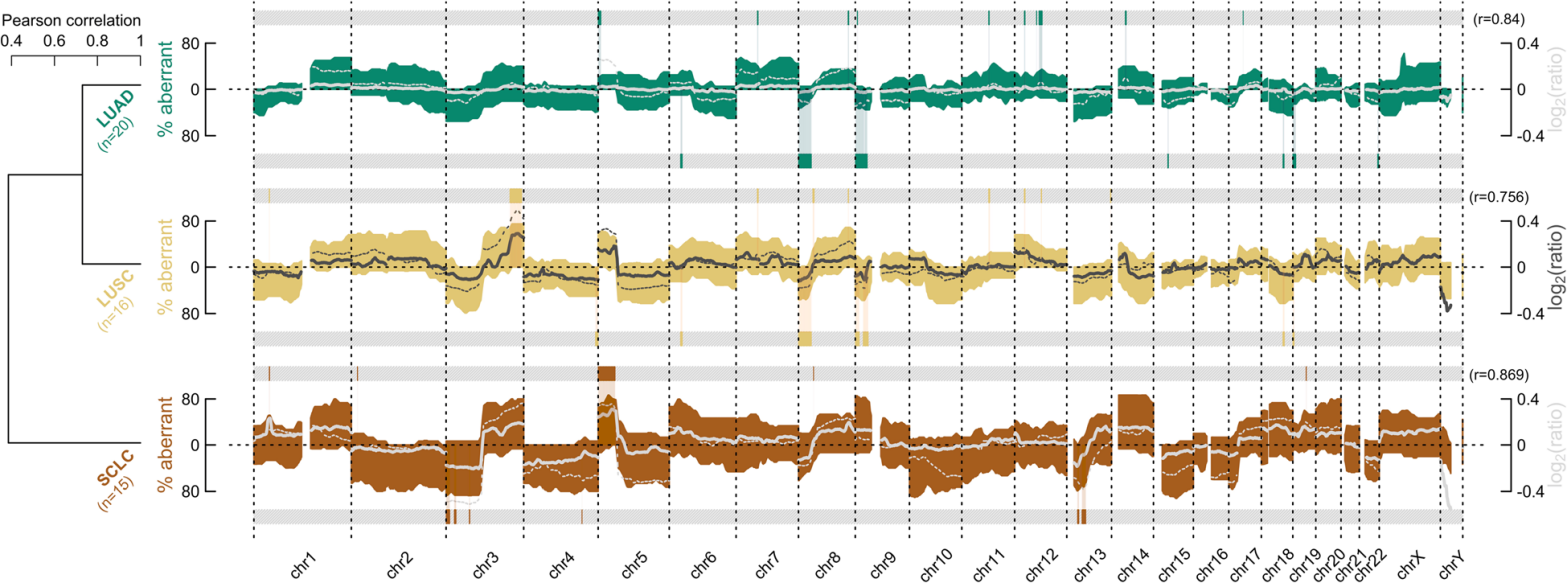
Overview of aberrations detected using liquid biopsies (LBs) in comparison to public data. Colored waves represent fractions of aberrant LBs per histological subtype. Patterns above the *x*-axes indicate gains, whilst opposite contours represent losses. The dendrogram (left) results from agglomerative complete-linkage clustering applied to the Pearson distances (i.e., *d* = (1 − *r*)/2) between these waves (vectors defined as %gains–%losses). Line graphs represent smoothened mean log_2_ ratios for LBs (thick solid) and the used large public dataset (thin dotted) [16]. The Pearson correlation (*r*) between these lines is given. Horizontal gray bars below and above each profile hold subtype-specific aberrant loci according to public data [16]

When summarizing all gains and losses per histological subtype, both lung cancer (e.g., gains at 5p) and distinct subtype-specific fingerprints are detected (Fig. 3). The overall correlation between the mean log_2_ ratios of the LBs and public SB data amounts to 0.840, 0.756, and 0.869 for LUAD, LUSC, and SCLC, respectively. In addition, non-supervised clustering applied to these resumptive profiles reflects histological hierarchy: NSCLC and SCLC are depicted as two separate entities. A sample-wise alternative cluster is shown in the supplement (Additional file 3: Figure S3).

To assess whether the high concordance with public data can be translated into a clinical application, the performance of a predictive model was assessed.

### A predictive model trained with public data performs well on liquid biopsies

Machine learning based on LBs is non-trivial as there are no conform training sets available at present. Nonetheless, the consortia CLCGP and NGM released data from large-scale genome-wide microarray experiments [16]. Although these data are thus array derived, in contrast to our study, superior training sets are currently not available. Five different multiclass classifiers were compared, followed by evaluating the in-house SBs and LBs using the former most performant model (see the “Methods”section).

**Fig. 4:**
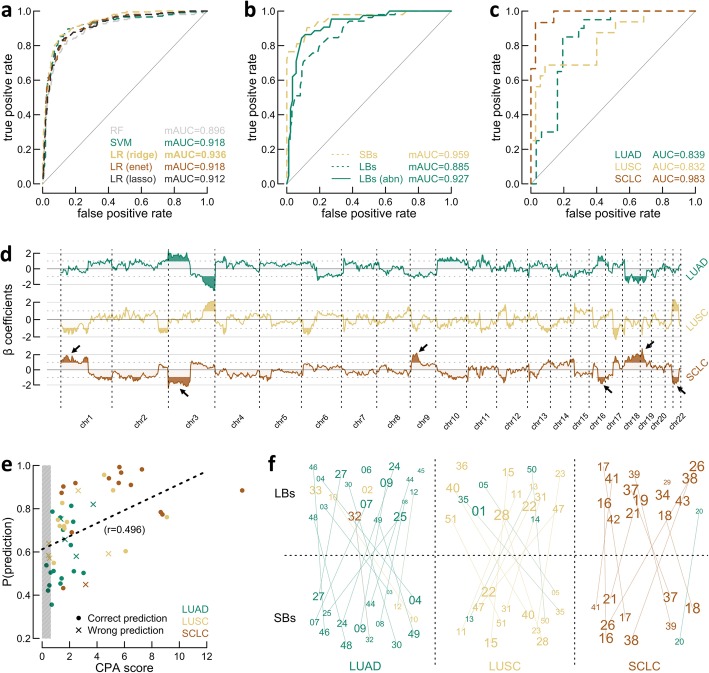
Multiclass predictive modeling for liquid biopsy (LB)-based histological classification. **a** One-vs-all receiver operating characteristic (ROC) analysis was executed in combination with leave-one-out cross-validation (LOOV) using the public training set for classifier selection [16]. Evaluated classifiers include random forest (RF); support vector machine (SVM); and logistic regression (LR) with ridge, elastic net (enet), and lasso regularization. Lines represent average ROC curves. Performance is quantified by the mean area under the curve (mAUC). **b** Solid (SBs) and LBs were evaluated with the best model (LR with ridge penalty) from **a**, using one-vs-all ROC analysis (dotted lines). Abnormal (abn) LBs, defined by copy number profile abnormality (CPA > 0.623), are shown separately in addition (solid line). **c** LBs evaluated using default ROC analysis. **d** β coefficients from the best model (LR with ridge penalty) from **a**. For perceptibility, the most prominent regions are colored (absolute value > 1), where the six most important loci to distinguish non-small cell lung cancer from small cell lung cancer (SCLC), according to the model, are emphasized by arrows. Coefficients were multiplied by 100. **e** Scatter plot of the relation between the CPA score and the prediction probability for LBs. The gray box (left) shows the 1% false discovery rate (FDR) cutoff. Colors indicate histology according to pathologists. The dotted line represents an ordinary least squares fit with the corresponding Pearson correlation (*r*). **f** Custom performance plot, where numbers represent patient IDs. Paired LBs (top) and SBs (bottom) are connected. Colors represent predicted type, grid position type according to pathologists (adenocarcinomas (LUADs), left; squamous cell carcinomas (LUSCs), central; SCLCs, right). The prediction probability linearly sets character size. Position within grid squares is random

Logistic regression with ridge regularization produced the best outcome according to an mAUC of 0.936, resulting from an iterative one-vs-all ROC analysis following training set LOOV (Fig. 4a). Where other learning strategies were discarded, the ridge model was enforced on the newly sequenced SBs, establishing an mAUC of 0.959 and an accuracy of 89.7% (Fig. 4b). The LBs however were predicted less precisely (mAUC of 0.885; accuracy of 80.4%), mainly because seven samples had no detectable aberrations in plasma: these were all predicted as LUAD, yet pathologists claimed three were LUSC. When dismissing cases without detectable aberrations, performance increased (mAUC of 0.927; accuracy of 84.1%). This was repeated across a range of possible CPA cutoffs (Additional file 3: Figure S4), showing that once the previously established 0.623 is reached, a more conservative cutoff does not necessary produce more accurate results. Finally, the ability to differentially diagnose SCLC from NSCLC employing LBs is given by an AUC of 0.983 and an accuracy of 96.1% (Fig. 4c). In comparison, during training set LOOV, this AUC amounted to 0.969.

Since the used logistic regression model appears to be reliable, its β coefficients (i.e., class-wise weights assigned to genomic loci to guide differential classification) were studied in detail (Fig. 4d). The six most prominent regions, according to the multinomial model, that discriminate NSCLC from SCLC are located at chromosome arm 1p (e.g., *MYCL1*), 3p (e.g., *FHIT*), 9p (e.g., *CDKN2A*), 16q, 19p (e.g., *STK11*), and chromosome 22. Revising the CNAs detected in the LBs (Fig. 3), it seems reasonable why these loci produce accurate predictions, e.g., chromosome arm 1p and 9p were frequently gained in SCLC, whilst they were more often lost in NSCLC, and chromosome arm 3p was deleted more often in SCLC.

It is not surprising that the prediction probability highly depends on the tumor fraction and thus the CPA score, as measured by a correlation of 0.496 (*P* < .001) (Fig. 4e). Therefore, because of the apparent inherent variability between the ctDNA fractions across the histological subgroups, SCLCs are favored for correct classification. Furthermore, SCLC is characterized by more distinct features in comparison to both NSCLC subtypes. Discriminating LUAD from LUSC was indeed expected to be intrinsically more ambitious, as previously demonstrated by cluster analysis (Fig. 3).

Amongst all wrongly predicted LBs (Fig. 4f), two (patients 10 and 20) were identically falsely classified based on their solid equivalent, three (patients 5, 14, and 35) resemble flat profiles due to an insufficient tumor fraction, three (patients 1, 20, and 33; Additional file 1: Table S1) were marked by pathologists for having an ambiguous histology prior to any in silico examination, and one (patient 50) concerned a patient with liver metastases identified with excessive heterogeneity (Additional file 3: Figure S2), possibly affecting the original classification.

### Copy number detection sensitivity can be improved by paired-end sequencing

PE sequencing at greater depths (1–1.5×) is expected to become increasingly feasible. Therefore, this section is dedicated to the latter technique as a “future prospect”: PE approaches could be favored over SE alternatives, since the acquired IS (i.e., cfDNA fragment size) information can be employed to enrich for tumor reads, as shorter DNA fragments are more likely to be tumor derived [17, 18]. We selected 29 LBs (especially wrong predictions and samples with insufficient ctDNA) for resequencing, after which copy number profiles were derived for raw PE data, IS filtered PE data, and randomly sampled PE data (as a “negative” control for IS filtered PE data) (see the “Methods” section).

**Fig. 5:**
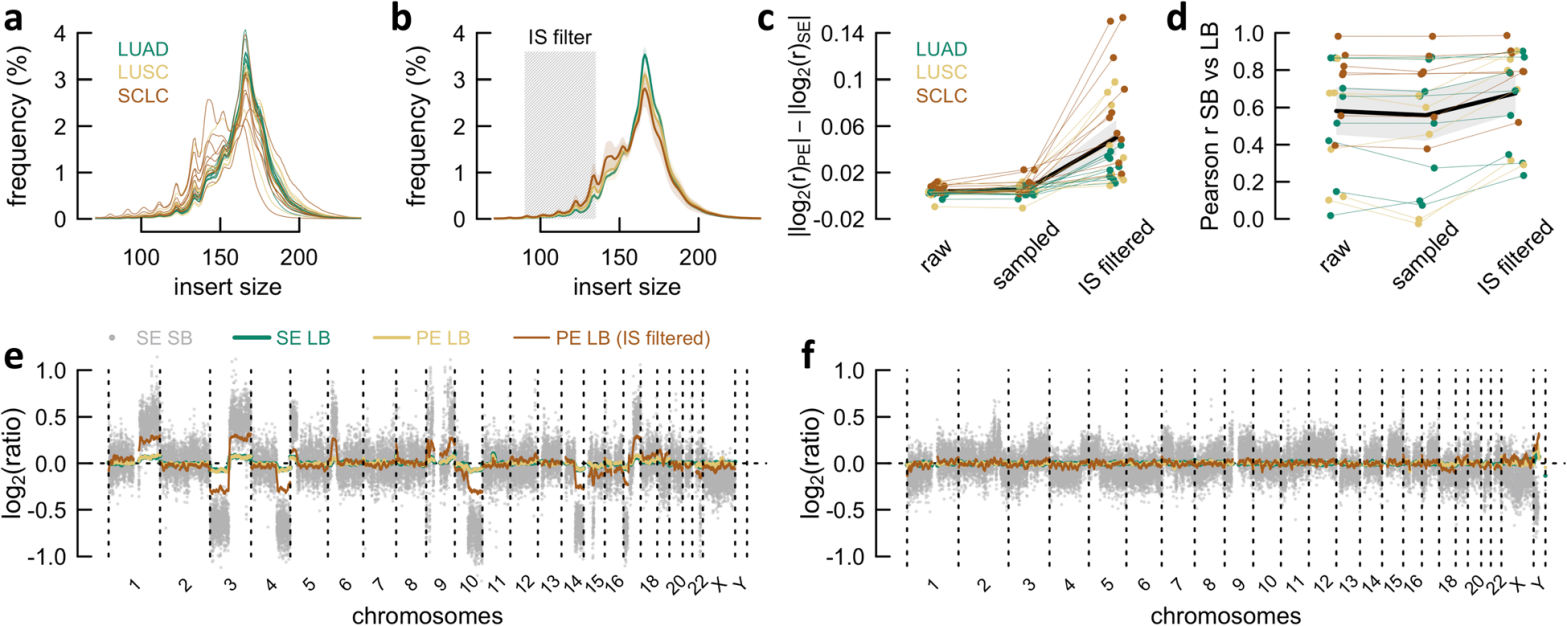
Paired-end (PE) sequencing and insert size (IS) filtering to increase sensitivity. **a** Sample-wise IS histograms of liquid biopsies (LBs). **b** Histology-wise mean IS histograms of LBs, with transparent background waves, which indicate the 95% CI. The gray box delineates the applied IS filter. **c** Comparison between three types of PE data: raw, randomly sampled, and sampled according to IS. The distance to the single-end (SE) profiles is shown for each instance. Thicker black line and gray background show mean and 95% CI interval, respectively. **d** Identical to **c**, yet for the Pearson correlation (*r*) with paired solid biopsies (SBs). **e** Copy number profiles of patient 41. The gray background dots represent the SB; line graphs on top are smoothened LB profiles, derived from SE data, PE data, and PE data, followed by IS filtering. **f** Identical to **e**, yet for patient 35

Following IS filtering (range 90–135 bp), on average, 4.31 million mapped reads (95% CI 2.40–6.22) remained. Sample-wise and histologically averaged IS distributions again signal that SCLCs release an abundance of ctDNA (Fig. 5a, b). As expected, segmental log_2_ ratios of copy number profiles differ little between SE and PE sequenced LBs (Fig. 5c). After IS filtering, however, the segmental amplitudes increased with an average of 0.050 (95% CI 0.035–0.065) in comparison to SE sequencing (Fig. 5c; Additional file 5). Similar observations were made for the LB/SB Pearson correlations, especially for patients with an originally low concordance (Fig. 5d).

The increase in absolute log_2_ ratio was most prominent for LBs with detectable tumor fraction, such as the SCLC samples (Fig. 5c)—patient 41, a representative SCLC case, demonstrates this effect elegantly (Fig. 5e). For “flat” SE profiles, this raise was less pronounced: although the correlation with the paired SB doubled, clear CNAs remain difficult to discriminate for patient 35 (Fig. 5f; Additional file 5).

In total, five LBs without detectable CNAs (CPA < 0.623) were resequenced, where three expressed significantly aberrant segments after IS filtering. Two of these concerned LUSC cases, previously misclassified as LUAD, yet now correctly classified following tumor read enrichment (patients 5 and 35; Additional file 5). To conclude, after substituting the original profiles with the tumor-enriched profiles, the overall mAUC raised from 0.885 to 0.912 (Additional file 3: Figure S5).

## Discussion

Molecular profiling was long challenged by instrumental bottlenecks and economic feasibility, yet recent technological advancements have largely overcome these obstacles. Tumor classification by next-generation sequencing is now expected to mature into the most informed avenue to direct therapeutic decisions; however, with the necessity of tumor tissue, associated intrinsic complications remain. In reaction, LBs could emerge as a complementary source—and, ultimately, an alternate practice—to obtain genomic information on tumors minimally invasive.

In this proof of concept study, we set out to examine 51 advanced stage lung cancer patients, using sWGS and copy number profiling. In 44 liquid samples, genomic aberrations could be identified. Plasmatic abnormality analysis revealed a significant difference between NSCLCs and SCLCs. Next, we noticed a high correlation in a subsequent concordance study between matched SBs and LBs once genomic aberrations were identifiable, despite the long interval times for several patients between pairs. Four patients did however express clear evidence of tumor heterogeneity, which could bias diagnosis using tissue-based methodologies. Our work further shows that LB-derived copy numbers can accurately differentiate SCLC from NSCLC, partly because SCLC ctDNA seems to be detectable with high sensitivity, and SCLCs are represented by distinct copy number profiles. This is highly relevant, as to date, correctly diagnosing SCLC is necessary and sufficient, due to the absence of effective targeted SCLC therapies. Differentiating LUAD versus LUSC, equally important to direct therapy, seems less accurate. Similar reasoning applies: profiles are less specific and ctDNA fractions are lower. The latter shortcoming, however, can be partly minimized by deeper PE sequencing: computational tumor enrichment based on IS statistics manages to increase the overall model performance.

Whilst some lung cancer studies have recently investigated LBs, currently none have truly examined their use for histological subtyping [25, 30–34]. To evaluate the adopted modeling approach, we therefore compared ours to other published tissue-based alternatives. The CLCGP and NGM study reports binary classification accuracies of 71.3%, 77.1%, and 91.7% for LUAD, LUSC, and SCLC, respectively (*n* = 637; including only samples with at least one genetic alteration) [14]. Using our strategy in combination with cross-validation, binary accuracies of respectively 86.8%, 84.8%, and 92.2% were settled on a class-balanced subset of the same public dataset (*n* = 204; no additional filtering). Two more large studies performed (binary) classification using copy numbers between LUAD and LUSC: the work of Li et al. claims an accuracy of 86.1% (*n* = 301), whilst Qiu and colleagues report 84.0% (*n* = 986) [35, 36]. Likewise, the in-house SBs (89.7%, 92.3%, and 97.4%, respectively) and LB (SE sequenced; 80.4%, 84.3%, and 96.1%, respectively) sets returned similar statistics.

Adopting discrete states (loss, copy neutral, gain) as model features (see the “Methods” section), which was favored to disregard variable tumor fractions, downweighs the presence of amplifications, indicated by, for example, patient 32: a 7p11.2 (*EGFR*) amplification, indicative for NSCLC, is clearly present (Additional file 4), yet the overall signal (wrongly) pushed classification towards SCLC [14]. Interesting in this context, small cell transformation of *EGFR* mutated LUAD is sometimes seen as a mechanism of resistance [37]. Transformation possibly occurred in patient 26, who was included at progressive stage and was subsequently correctly classified by the model as SCLC. A baseline SB was sequenced in addition to the main cohort, exposing initial LUAD-specific aberrations (Additional file 3: Figure S6).

To mimic a clinically realistic cohort, diagnostically challenging cases were not excluded. Moreover, the set comprised small biopsies and eight cytological specimens (Additional file 1: Table S2). Central pathology review identified five cases with divergent immunohistochemical results: these were therefore annotated with a genuine uncertainty (Additional file 1: Table S1). The most interesting case, patient 20, was the sole wrong prediction amongst the SCLCs. Its SB, representing a brain metastasis that was originally morphologically classified as SCLC, showed atypical small cell features on review: cell nuclei were rather large with sometimes prominent nucleoli, suggesting large cell neuroendocrine carcinoma [38]. Using additional immunohistochemistry analyses, RB1 seemed copy neutral and p53 resulted in a wild-type pattern (unfortunately, no information on mutations could be obtained, as *TP53* sequencing failed due to coverage issues). However, as described for small cell-like large cell neuroendocrine carcinoma, a high proliferation index of 80% was documented by Ki67 staining. Therefore, based on morphology and immunohistochemistry, patient 20 remained difficult to categorize and could thus concern either SCLC or large cell neuroendocrine carcinoma.

A considerable advantage of sWGS is the convenience by which LBs for copy number profiling could be implemented as routine practice in molecular diagnostic laboratories. To clarify, NIPT has evolved into a daily executed application, and in essence, it concerns the same technological and laboratory steps. A turnaround time of less than 4 days and a total (i.e., including processing costs) price tag of roughly $200 could be expected.

Classification according to morphology and immunohistochemistry is sometimes subjective, as it is not consistently a black-and-white story. Therefore, a computationally processed copy number profile could be of considerable help as an addition to current traditional diagnostic methods. However, our conclusions should be confirmed on larger independent datasets.

To conclude, this work reveals the presence of rather surprisingly large amounts of aberrant ctDNA, especially for SCLC. This realization offers opportunities in future research: introducing single nucleotide variants, obtained from targeted sequencing, to current classification model, seems largely feasible. It is likely that in the near future, combinations of these sequencing methods could tackle molecular complexity beyond histology: given a patient with a clinical picture suspicious for advanced stage lung cancer, a diagnostic LB could offer both accurate classification and all necessary sequencing information to direct precision medicine.

## Conclusions

We demonstrate, as a proof of principle, that copy number profiling of cell-free DNA can be used to differentiate NSCLC from SCLC. The central technique, sWGS, is inexpensive and often fully operational at routine molecular laboratories. These concepts therefore have relevant diagnostic potential, especially for patients with lesions that are difficult to reach—all the more since correctly diagnosing SCLC is sufficient to initiate therapy.

## Supplementary information

**Additional file 1: Table S1** Patient information. **Table S2** Sample information.

**Additional file 2: Supplementary methods.** The copy number profile abnormality score & Details on predictive modeling.

**Additional file 3: Figure S1** Performance comparison between the copy number profile abnormality (CPA) score and previously published alternatives, applied to all liquid biopsies. **Figure S2** Tumor heterogeneity analysis, applied to patients with a liquid (LB) and solid biopsy (SB) taken no longer than 50 days apart. **Figure S3** Cluster analysis, applied to all liquid biopsies. **Figure S4** Prediction accuracy in relation to abnormality cutoff stringency. **Figure S5** Performance of predicting histology by single-end (SE) versus paired-end (PE) sequencing. **Figure S6** Copy number profiles of a relapsed patient with small cell transformation/a second primary tumor.

**Additional file 4.** Supplementary single-end copy number profiles, containing all in-house lung cancer copy number profiles.

**Additional file 5.** Supplementary paired-end copy number profiles, containing a thorough patient-wise comparison of paired single/paired-end copy number profiles. Prediction probabilities are shown in addition.

**Additional file 6.** Copy number profile data of solid biopsies (single-end).

**Additional file 7.** Copy number profile data of liquid biopsies (single-end).

**Additional file 8.** Copy number profile data of liquid biopsies (paired-end).

**Additional file 9.** Copy number profile data of liquid biopsies (paired-end; insert size filtered).

## Data Availability

Copy number data generated and analyzed for this study are available in Additional files 6, 7, 8, and 9. Public data can be downloaded from the supplement of the CLCGP and NGM study [16].

## Abbreviations

NSCLC: Non-small cell lung cancer
LUAD: Lung adenocarcinoma
LUSC: Lung squamous cell carcinoma
SCLC: Small cell lung cancer
LB: Liquid biopsy
cfDNA: Cell-free DNA
sWGS: Shallow whole-genome sequencing
CNA: Copy number alteration
SNP: Single nucleotide polymorphism
FFPE: Formalin-fixed paraffin-embedded
SB: Solid biopsy
NIPT: Non-invasive prenatal testing
CLCGP: Clinical Lung Cancer Genome Project (consortium)
NGM: Network Genome Medicine (consortium)
SE: Single-end
PE: Paired-end
IS: Insert size
PGA: Plasma genomic abnormality
CPA: Copy number profile abnormality
FDR: False discovery rate
LOOV: Leave-one-out cross-validation
(m)AUC: (mean) Area under the curve
ROC: Receiver operating characteristic
ctDNA: Circulating tumor DNA
CI: Confidence interval
RF: Random forest
SVM: Support vector machine
LR: Logistic regression
ENET: Elastic net
