# Supplementary material for: Shallow whole-genome sequencing of plasma cell-free DNA accurately differentiates small from non-small cell lung carcinoma
Source: Genome Med. 2020 Apr 21;12:35. doi: 10.1186/s13073-020-00735-4 (PMC7175544; doi:10.1186/s13073-020-00735-4)
Supplement: Supplementary file 1 — Additional file 1: Table S1 Patient information. Table S2 Sample information. [file 13073_2020_735_MOESM1_ESM.docx]

| **Table S1** Selection criteria applied during patient recruitment were based on histology and stage (exclusively III and IV). All cases were reviewed by central pathology review (CPR). In case of doubt about the true histology, the potential alternative is given between brackets, where ‘O’ indicates other than lung adenocarcinoma (LUAD), lung squamous cell carcinoma (LUSC) or small cell lung cancer (SCLC). | | | | | | | **Table S2** Both liquid and, when available, tissue biopsies were collected from the recruited patients. *Abbreviations*: LB, liquid biopsy; SB, solid biopsy; WBRT, whole brain radiotherapy; FNA, fine-needle aspiration; EBUS, endobronchial ultrasound; EUS, endoscopic ultrasound. *Symbols*: †, sequenced paired-end in addition; *, cytological specimen. | | | | |
| --- | --- | --- | --- | --- | --- | --- | --- | --- | --- | --- | --- |
| ID | Sex | Age | Smoking status | Histology (CPR) | TNM | Stage | LB at … | SB at … | SB site (type) | LB - SB (days) |  |
| 1 | Female | 65 | Never | LUSC (LUAD) | TxN0M1b | IV | Primary diagnosis† | - | - | - |  |
| 2 | Male | 66 | Former | LUAD | - | IV | Progressive disease† | - | - | - |  |
| 3 | Male | 72 | Former | LUAD | T3N3M1b | IV | Progressive disease† | Primary diagnosis | Bronchus (biopsy) | 210 |  |
| 4 | Female | 64 | Former | LUAD | T1bN2M1b | IV | After WBRT† | After WBRT | Lung (biopsy) | 51 |  |
| 5 | Male | 69 | Active | LUSC | T3N0M1c | IV | Primary diagnosis† | Primary diagnosis | Lung (biopsy) | 35 |  |
| 6 | Female | 54 | Active | LUAD | - | IV | Progressive disease | - | - | - |  |
| 7 | Male | 78 | Never | LUAD | T3N0M1b | IV | Relapse | Relapse | Skin (biopsy) | 18 |  |
| 8 | Male | 80 | Active | LUAD | T4N3M1c | IV | Primary diagnosis† | Primary diagnosis | Adrenal gland (FNA*) | 33 |  |
| 9 | Male | 75 | Former | LUAD | T2bN2M1c | IV | Primary diagnosis | Primary diagnosis | Bronchus (biopsy) | 25 |  |
| 10 | Female | 60 | Active | LUAD | T3N0M1c | IV | After WBRT† | Primary diagnosis | Brain (biopsy) | 63 |  |
| 11 | Female | 63 | Former | LUSC (O) | T3N3M1b | IV | Primary diagnosis† | Primary diagnosis | Kidney (biopsy) | 12 |  |
| 12 | Female | 65 | Former | LUAD (LUSC) | T3N2M1b | IV | During treatment | Primary diagnosis | Bronchus (biopsy) | 42 |  |
| 13 | Male | 64 | Active | LUSC | T4N3M1b | IV | Progressive disease† | Primary diagnosis | Liver (biopsy) | 221 |  |
| 14 | Male | 76 | Active | LUSC | T3N2M1a | IV | Primary diagnosis† | - | - | - |  |
| 15 | Male | 44 | Active | LUSC | - | IV | Progressive disease† | Primary diagnosis | Lung (biopsy) | 504 |  |
| 16 | Male | 71 | Active | SCLC | T4N3M0 | III | Primary diagnosis | Primary diagnosis | Bronchus (biopsy) | 8 |  |
| 17 | Female | 73 | Former | SCLC | T4N3Mx | III | Primary diagnosis† | Primary diagnosis | Bronchus (biopsy) | 42 |  |
| 18 | Female | 53 | Active | SCLC | T4N2M1b | IV | Relapse† | Primary diagnosis | Bronchus (biopsy) | 280 |  |
| 19 | Male | 46 | Former | SCLC | T1bN0M1b | IV | Relapse | - | - | - |  |
| 20 | Female | 48 | Former | SCLC (O) | T4N3M1b | IV | Progressive disease† | Primary diagnosis | Brain (biopsy) | 140 |  |
| 21 | Male | 74 | Former | SCLC | T4N3M1c | IV | Primary diagnosis | Primary diagnosis | Bronchus (biopsy) | 19 |  |
| 22 | Male | 72 | Former | LUSC | T4N0Mx | III | Progressive disease | Primary diagnosis | Bronchus (biopsy) | 345 |  |
| 23 | Male | 71 | Former | LUSC | T2bN3M1b | IV | Progressive disease | Primary diagnosis | Bronchus (biopsy) | 102 |  |
| 24 | Female | 53 | Active | LUAD | T1N3M1b | IV | Primary diagnosis | Primary diagnosis | Lymph node (EBUS*) | 54 |  |
| 25 | Male | 57 | Former | LUAD | T3N3M1b | IV | Primary diagnosis | Primary diagnosis | Lymph node (EUS*) | 29 |  |
| 26 | Male | 71 | Former | SCLC | T1bN3M0 | III | Progressive disease | Progressive disease | Bronchus (biopsy) | 97 |  |
| 27 | Male | 57 | Former | LUAD | T2aN2M1c | IV | Primary diagnosis† | Primary diagnosis | Lymph node (EBUS*) | 0 |  |
| 28 | Male | 52 | Former | LUSC | T3N2M1c | IV | Progressive disease | Primary diagnosis | Lymph node (EBUS*) | 308 |  |
| 29 | Male | 78 | Former | SCLC | T1aN2M0 | III | Progressive disease | - | - | - |  |
| 30 | Female | 71 | Active | LUAD | T2bN2M1b | IV | Primary diagnosis† | Primary diagnosis | Lymph node (EBUS*) | 13 |  |
| 31 | Female | 64 | Former | LUSC | T4N3M0 | III | Primary diagnosis | Primary diagnosis | Lung (resection) | -11 |  |
| 32 | Female | 55 | Never | LUAD | T2bN2M1b | IV | After WBRT† | After WBRT | Bronchus (biopsy) | 44 |  |
| 33 | Male | 76 | Former | LUAD (LUSC) | - | IV | Primary diagnosis† | - | - | - |  |
| 34 | Male | 69 | Former | SCLC | - | IV | Primary diagnosis† | - | - | - |  |
| 35 | Male | 52 | Active | LUSC | T4N1M1c | IV | Primary diagnosis† | Primary diagnosis | Bronchus (biopsy) | 40 |  |
| 36 | Male | 76 | Former | LUSC | T4N3M1c | IV | Relapse† | - | - | - |  |
| 37 | Male | 61 | Former | SCLC | - | IV | Primary diagnosis† | Primary diagnosis | Bronchus (biopsy) | 6 |  |
| 38 | Male | 65 | Former | SCLC | TxN0M1c | IV | Relapse† | Primary diagnosis | Bronchus (biopsy) | 507 |  |
| 39 | Female | 71 | Former | SCLC | - | IV | Primary diagnosis† | Primary diagnosis | Bronchus (biopsy) | 9 |  |
| 40 | Male | 51 | Active | LUSC | T4N2M0 | III | Primary diagnosis† | Primary diagnosis | Bronchus (biopsy) | 45 |  |
| 41 | Female | 63 | Active | SCLC | - | IV | After WBRT† | After WBRT | Bronchus (biopsy) | 14 |  |
| 42 | Female | 67 | Active | SCLC | T1N2Mx | IV | Primary diagnosis† | - | - | - |  |
| 43 | Male | 62 | Active | SCLC | - | IV | Primary diagnosis† | - | - | - |  |
| 44 | Female | 54 | Former | LUAD | rTN3M1c | IV | Relapse† | Relapse | Lymph node (biopsy) | 21 |  |
| 45 | Male | 66 | Former | LUAD | T4N0M1a | IV | Primary diagnosis | - | - | - |  |
| 46 | Female | 52 | Active | LUAD | T4N2M1c | IV | Primary diagnosis | Primary diagnosis | Bronchus (biopsy) | 27 |  |
| 47 | Male | 76 | Former | LUSC | T3N3M1b | IV | Primary diagnosis | Primary diagnosis | Bronchus (biopsy) | 31 |  |
| 48 | Female | 66 | Former | LUAD | N3M1b | IV | Primary diagnosis | Primary diagnosis | Lymph node (EBUS*) | 29 |  |
| 49 | Male | 66 | Former | LUAD | T3N1M1c | IV | Primary diagnosis | Primary diagnosis | Bronchus (biopsy) | 18 |  |
| 50 | Male | 76 | - | LUSC | T4N2M1c | IV | Primary diagnosis | Primary diagnosis | Bronchus (biopsy) | 7 |  |
| 51 | Male | 76 | Former | LUSC | T4N1M1a | IV | Primary diagnosis | Primary diagnosis | Lymph node (EBUS*) | 0 |  |
