# Supplementary material for: Shallow whole-genome sequencing of plasma cell-free DNA accurately differentiates small from non-small cell lung carcinoma
Source: Genome Med. 2020 Apr 21;12:35. doi: 10.1186/s13073-020-00735-4 (PMC7175544; doi:10.1186/s13073-020-00735-4)
Supplement: Supplementary file 2 — Additional file 2: Supplementary methods. The copy number profile abnormality score & Details on predictive modeling. [file 13073_2020_735_MOESM2_ESM.docx]

**Supplementary methods**

[The copy number profile abnormality (CPA) score 1](#_Toc37147689)

[Performance comparison 1](#_Toc37147690)

[Structure of the formula 1](#_Toc37147691)

[Details on predictive modeling 2](#_Toc37147692)

[Features 2](#_Toc37147693)

[Training 2](#_Toc37147694)

[Testing 3](#_Toc37147695)

# The copy number profile abnormality (CPA) score

## Performance comparison

The CPA score is designed to distinguish abnormal from healthy copy number profiles (i.e., ‘flat’ profiles, disregarding natural variation). This means that this variable is not directly proportional to the tumor fraction, yet it is evidently highly influenced by the tumor fraction. To establish its performance, the CPA score was compared to ichorCNA (*details at the end of this section) and plasma genomic abnormality (PGA) values (Additional file 3: Fig S1a-c)—both are published alternatives to quantify tumor burden. Three cases were selected (Additional file 3: Fig S1d-f) to further practically demonstrate our approach’s power in comparison to these previously released efforts.

## Structure of the formula

Another way to express the CPA score, in comparison to what is shown in the main paper, is given below:

$$CPA={\sum_{i=0}^{n} \left( \left| Z_{{segment}_{i}} \right|*l_{{segment}_{i}} \right)}/{\sum_{i=0}^{n} l_{{segment}_{i}}}* {\sum_{i=0}^{n} l_{{segment}_{i}}}/n$$

The first part of this formula represents the mean of all bin-wise interpolated absolute segmental Z-scores. There are three main reasons for its structure. (1) By using a bin-wise **average**, the formula is assumed to be largely independent of the number of bins, which differs between males and females. (2) Small sequencing depth differences do not influence the CPA score, since **segmental values** rather than the raw bin-wise ones are used—the latter are heavily influenced by Gaussian noise, which is directly correlated with sequencing depth. (3) **Z-scores** are considered rather than log_2_ ratios, as the first account for naturally occurring healthy variability. For instance, a deviation at the highly variable Y chromosome will contribute less to an increased CPA score than an apparent equal alteration at chromosome 1. The second part, holding the average segment length, represents a penalty term for sample quality. In general, short segments are exclusively observed in *bad quality* or *truly highly aberrant* samples. In the first case, the penalty term ensures the CPA score doesn’t skyrocket (e.g. Additional file 3: Fig S1e); whilst in the second case, segmental amplitudes are more extreme, which overcomes this penalty (e.g. patient 34; Additional file 4).

** ichorCNA v0.2.0 was used. Parameter arguments were set to allow three subclonal states; the maximum copy number was set to 5; the optimization algorithm was reinitiated using five different starting values, representing the non-tumoral contamination: 20%, 35%, 50%, 65% and 80%. The obtained tumor fraction corresponding to the highest likelihood was interpreted as the final estimated tumor fraction.*

# Details on predictive modeling

With predictive modeling (or machine learning), a classifier (or model) transforms a selection of features (processed copy number data) to a response variable (LUAD, LUSC or SCLC). With classification, this process eventually produces class probabilities (P):

$P\left( LUAD \right)=classifier(features)$ $P(LUSC)=classifier(features)$

$P\left( SCLC \right)=classifier\left( features \right)$ $P\left( LUAD \right)+P\left( LUSC \right)+P\left( SCLC \right)=1$

## Features

To reduce tumor fraction bias/variability, three states were considered: -1 (loss), 0 (copy neutral) and +1 (gain). Two different strategies to obtain these features were applied, depending the available input information.

1. **The** **public training dataset** exclusively contains segmental continuous copy number states, mapped to GRCh37 coordinates. Using the R library *rtracklayer* (v1.42.1), which depends on UCSC’s liftOver tool, all coordinates were translated to match the GRCh38 reference. Liftover was successful for 94.93% of the loci. For the remainder, the GRCh37 coordinates were kept—in a 100 kb binned setting, these were assumed be mostly reliable. Continuous copy number state hard cutoffs of <1.8 and >2.2 designated the discrete segmental states as described above.
2. **For the in-house data,** consult the main paper.

Finally, for both (1) and (2), all states were mapped to 100 kb bins across the GRCh38 genome (30,894 bins). Bins where at least one patient had missing data were excluded. Since the gonosomes were not correctly normalized in the public data, these were also excluded from the analysis. After these filtering steps, 24017 (77.7%) bins remained.

## Training

Five classifiers were evaluated using leave-one-out cross-validation (LOOV) executed on the training set. The latter set exclusively contained public samples. Since some classifiers cannot deal with unbalanced data (LUAD, n=424; LUSC, n=351; SCLC, n=68), the set was class-balanced (LUAD, n=68; LUSC, n=68; SCLC, n=68) by random sampling. All evaluated classifiers with corresponding parameters are detailed below:

1. **Random forest** (using R package *iRF*; v2.0.2). All parameters remained default.
2. **Support vector machine** (using R package *e1071*; v1.7.1). The gamma parameter during LOOV was set to 0.001, after evaluating nine logarithmically ranked gamma possibilities (0.1, 0.01, 0.001, …) with the *tune.svm* function.
3. **Multinomial logistic regression with ridge regularization** (using R package *glmnet*; v2.0.16). The elastic net mixing parameter (alpha) was set to 0 (ridge penalty). Lambda (the amount of regularization), was optimized using the *cv.glmnet* function. The lambda corresponding to the minimal multinomial deviance was passed to LOOV.
4. **Multinomial logistic regression with elastic net regularization** (using R package *glmnet*; v2.0.16). The elastic net mixing parameter (alpha) was set to 0.5 (elastic net penalty). Lambda was optimized as above.
5. **Multinomial logistic regression with lasso regularization** (using R package *glmnet*; v2.0.16). The elastic net mixing parameter (alpha) was set to 1 (lasso penalty). Lambda was optimized as above.

The performance of each classifier was established using an iterative one-vs-all (LUAD vs LUSC and SCLC; LUSC vs LUAD and SCLC; SCLC vs LUAD and LUSC) receiver operation characteristic (ROC) analysis in combination with the mean area under the curve (mAUC). The most performant model was retrained using the complete training set (now including the ‘left out sample’).

## Testing

All newly sequenced in-house solid and liquid biopsies were evaluated using the final model. The one-vs-all mAUC was again deployed to compare the performance of the SBs with the LBs, whereas the one-vs-all AUC was used to judge to what extent the model can differentiate certain classes from others within LBs.
