# Supplementary material for: Shallow whole-genome sequencing of plasma cell-free DNA accurately differentiates small from non-small cell lung carcinoma
Source: Genome Med. 2020 Apr 21;12:35. doi: 10.1186/s13073-020-00735-4 (PMC7175544; doi:10.1186/s13073-020-00735-4)
Supplement: Supplementary file 3 — Additional file 3: Figure S1 Performance comparison between the copy number profile abnormality (CPA) score and previously published alternatives, applied to all liquid biopsies. Figure S2 Tumor heterogeneity analysis, applied to patients with a liquid (LB) and solid biopsy (SB) taken no longer than 50 days apart. Figure S3 Cluster analysis, applied to all liquid biopsies. Figure S4 Prediction accuracy in relation to abnormality cutoff stringency. Figure S5 Performance of predicting histology by single-end (SE) versus paired-end (PE) sequencing. Figure S6 Copy number profiles of a relapsed patient with small cell transformation/a second primary tumor. [file 13073_2020_735_MOESM3_ESM.docx]

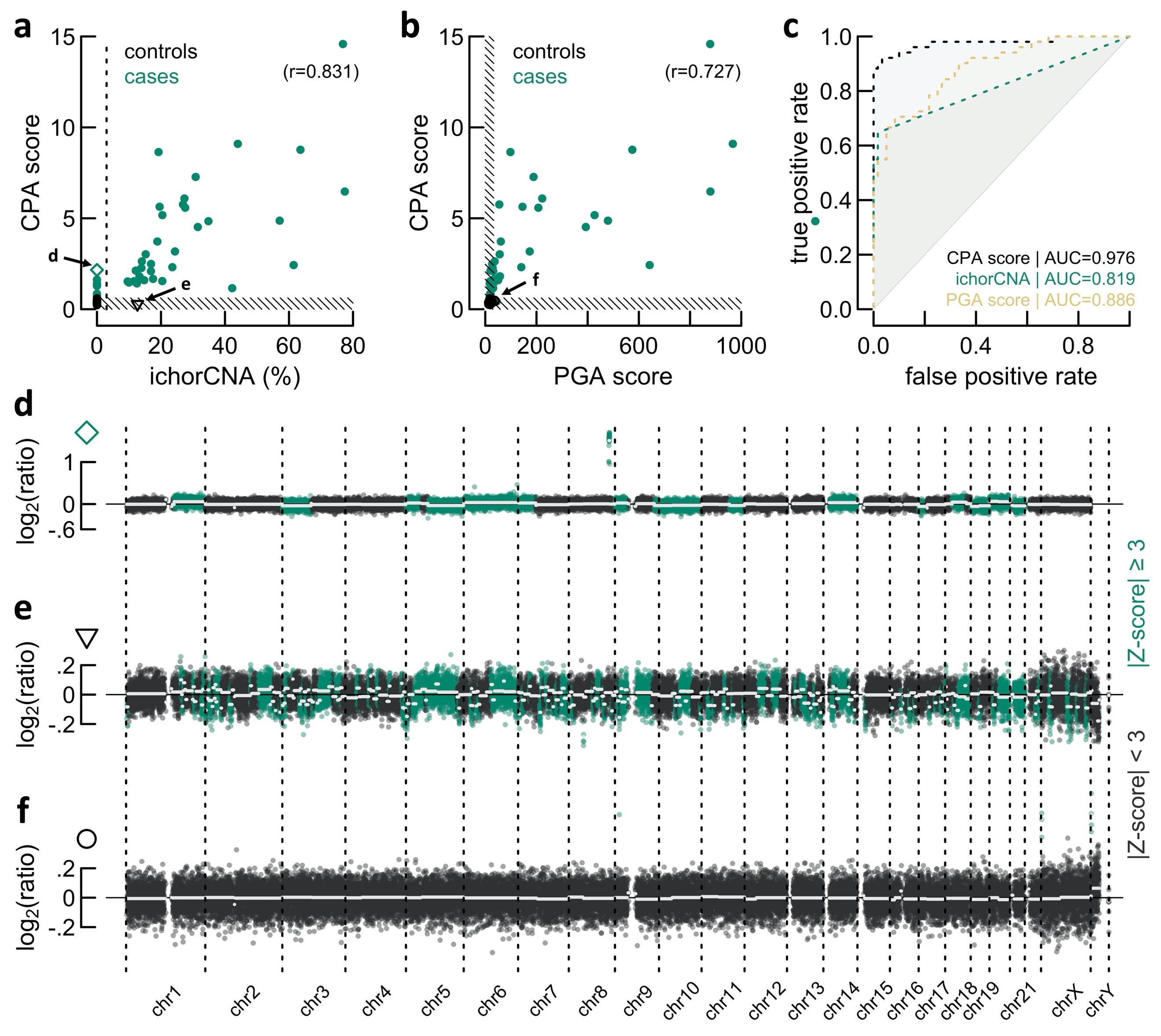


**Fig. S1** Performance comparison between the copy number profile abnormality (CPA) score and previously published alternatives, applied to all liquid biopsies. **a** Scatter plot comparison between the tumor DNA fraction inferred by ichorCNA and the CPA score. The Pearson correlation coefficient (r) is shown. Two cases, emphasized by different symbols, are detailed at **d** and **e**. The horizontal box represents the 1% false discovery rate (FDR) cutoff associated to the CPA score, whereas the vertical dotted line depicts ichorCNA’s 3% tumor fraction lower detection limit. **b** Scatter plot comparison between the profile genomic abnormality (PGA) and the CPA score. The Pearson correlation coefficient (r) is shown. One case, indicated by a different symbol, is visualized at **f**. The boxes represent the 1% FDR limits. **c** Receiver operating characteristic (ROC) analysis to measure tumor detection accuracy across the considered variables, by means of the area under the curve (AUC). **d** Copy number profile of patient 39. Green dots represent bins positioned in segments with an absolute Z-score of 3 or more. Subtle gains (e.g. 1q) and losses (e.g. 3p and 5q), and one amplification (at 8q), are visible. These detections seem reliable, given the large correlation with a paired solid biopsy (Additional file 4: patient 39). However, ichorCNA seems to be subject to inadequate sensitivity, as the tumor fraction is claimed to be 0%, whilst the CPA score does imply abnormality. **e** Copy number profile of a control subject. The highly scattered segmental pattern originates from low sample quality (presumably partly degraded cell-free DNA). Here, CPA’s penalty term (Additional file 2) ensures the overall score doesn’t skyrocket. According to ichorCNA, this control sample results from a case with >10% tumor DNA fraction, rendering it a false positive. **f** Copy number profile from a control subject sequenced at low depth (<10M reads). Despite the absence of green bins (meaning that no absolute segmental Z-score exceeds 3), the PGA approach proposes that this sample is abnormal—this because bin-wise values, which are subject to natural Gaussian noise, are used in the PGA formula. In contrast, the CPA score implements segmental values (Additional file 2), which are less influenced by read coverage bias. Moreover, the apparent deviation at chromosome Y barely alters the CPA, as the formula adopts Z-scores instead of log_2_ ratios (Additional file 2), which is justified, since the Y chromosome is naturally subject to variation.


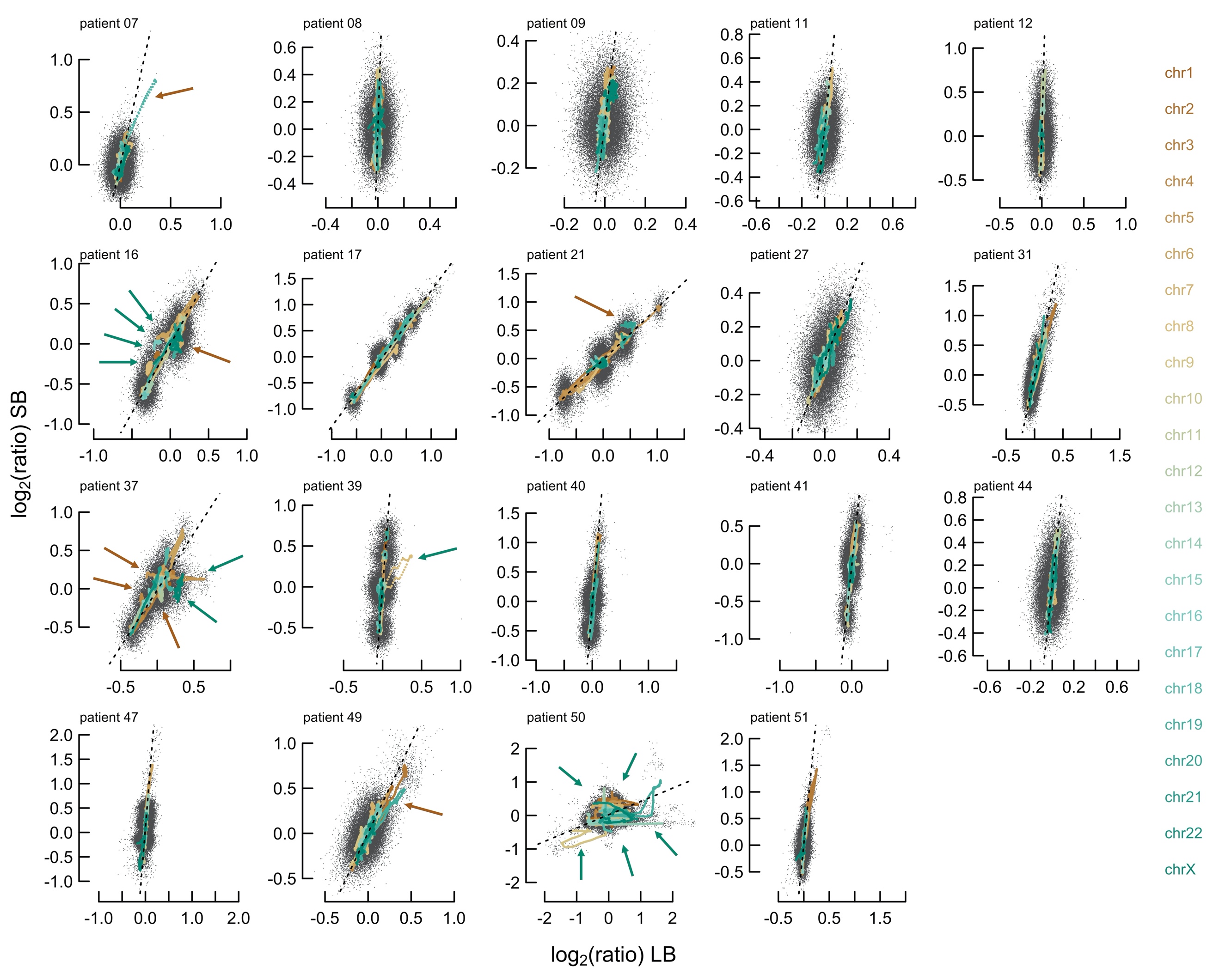


**Fig. S2** Tumor heterogeneity analysis, applied to patients with a liquid (LB) and solid biopsy (SB) taken no longer than 50 days apart. Scatter plot matrix visualizes LBs in relation to paired SBs. Black background dots represent raw log_2_ ratios. Patterns on top, colored according to chromosomal origin, show smoothened profiles, derived as in Fig. 1. Black dotted lines result from total least squares analyses. Clear same-chromosome trend deviations, caused by heterogeneity, are emphasized using green arrows. Brown arrows point towards subtle anomalies, which merely suggest heterogeneity. Patients represented by one or two ‘flat’ (copy number profile abnormality score < 0.623) profiles were excluded.


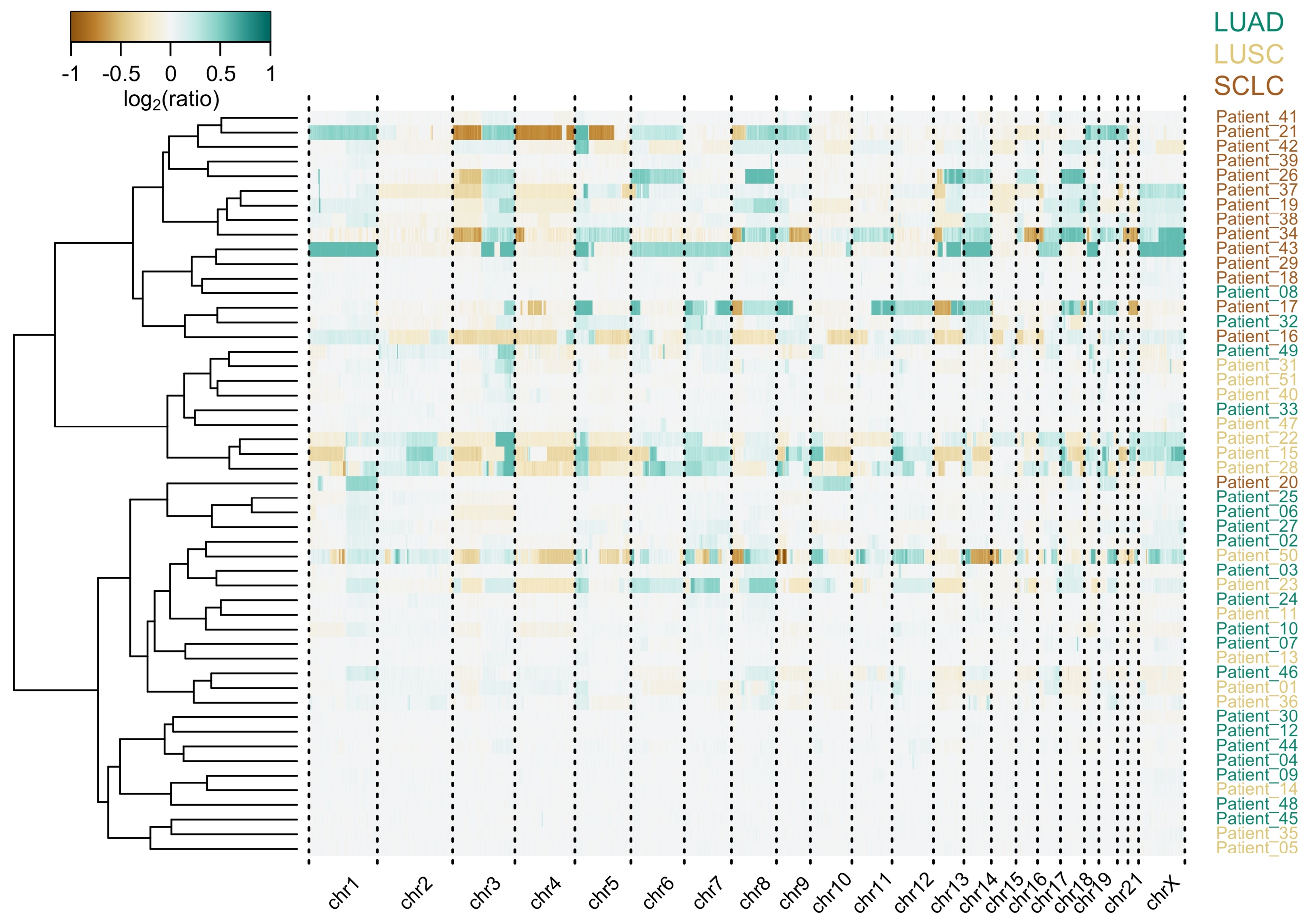


**Fig. S3** Cluster analysis, applied to all liquid biopsies. Heatmap represents bin-wise log_2_ ratios. To minimize tumor fraction bias, the Pearson correlation was used as a distance measure (i.e., $d=(1-r)/2$) between profiles. Hierarchical clustering was executed using Ward’s method.


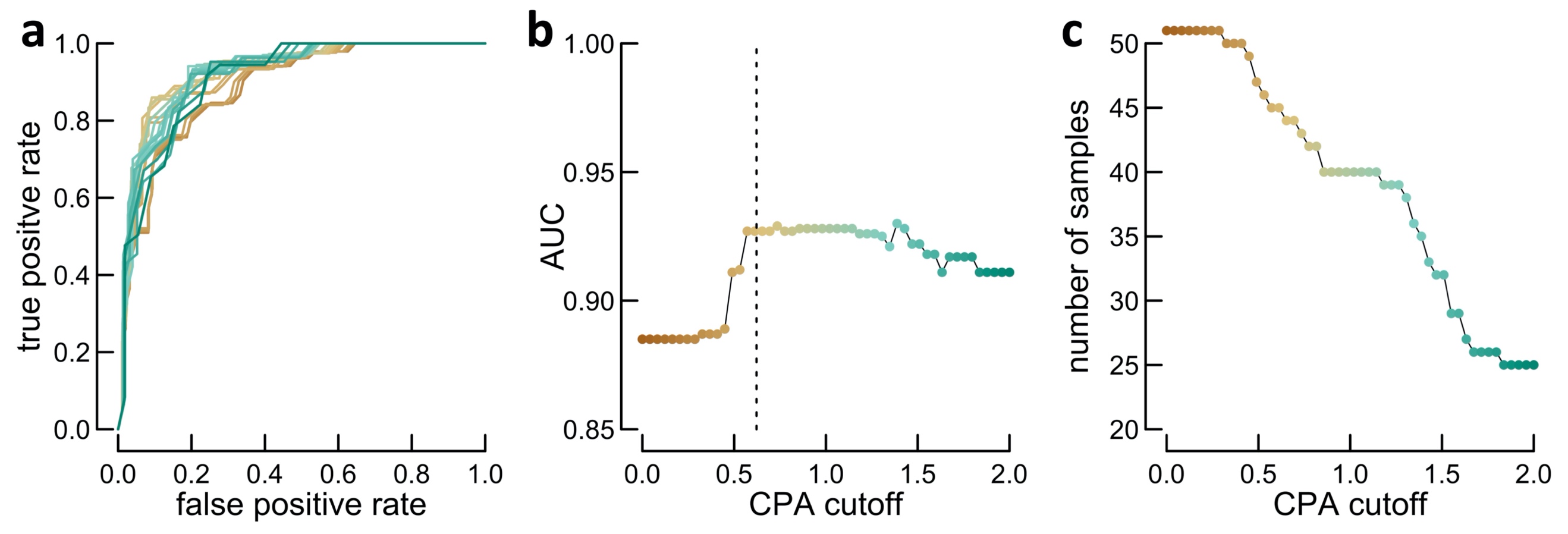


**Fig. S4** Prediction accuracy in relation to abnormality cutoff stringency. **a** Receiver operating characteristic (ROC) curves, matching different copy number profile abnormality (CPA) cutoffs (i.e., samples with a CPA lower than the limit are omitted). Colors are assigned in accordance to **b**. **b** The area under the curve (AUC), corresponding to ROC curves from **a**, in relation to the CPA cutoff. The vertical dotted line indicates the CPA cutoff for abnormality calling. Dots are colored using a linear gradient across 50 linearly defined cutoffs. **c** The number of samples remaining at each CPA cutoff.


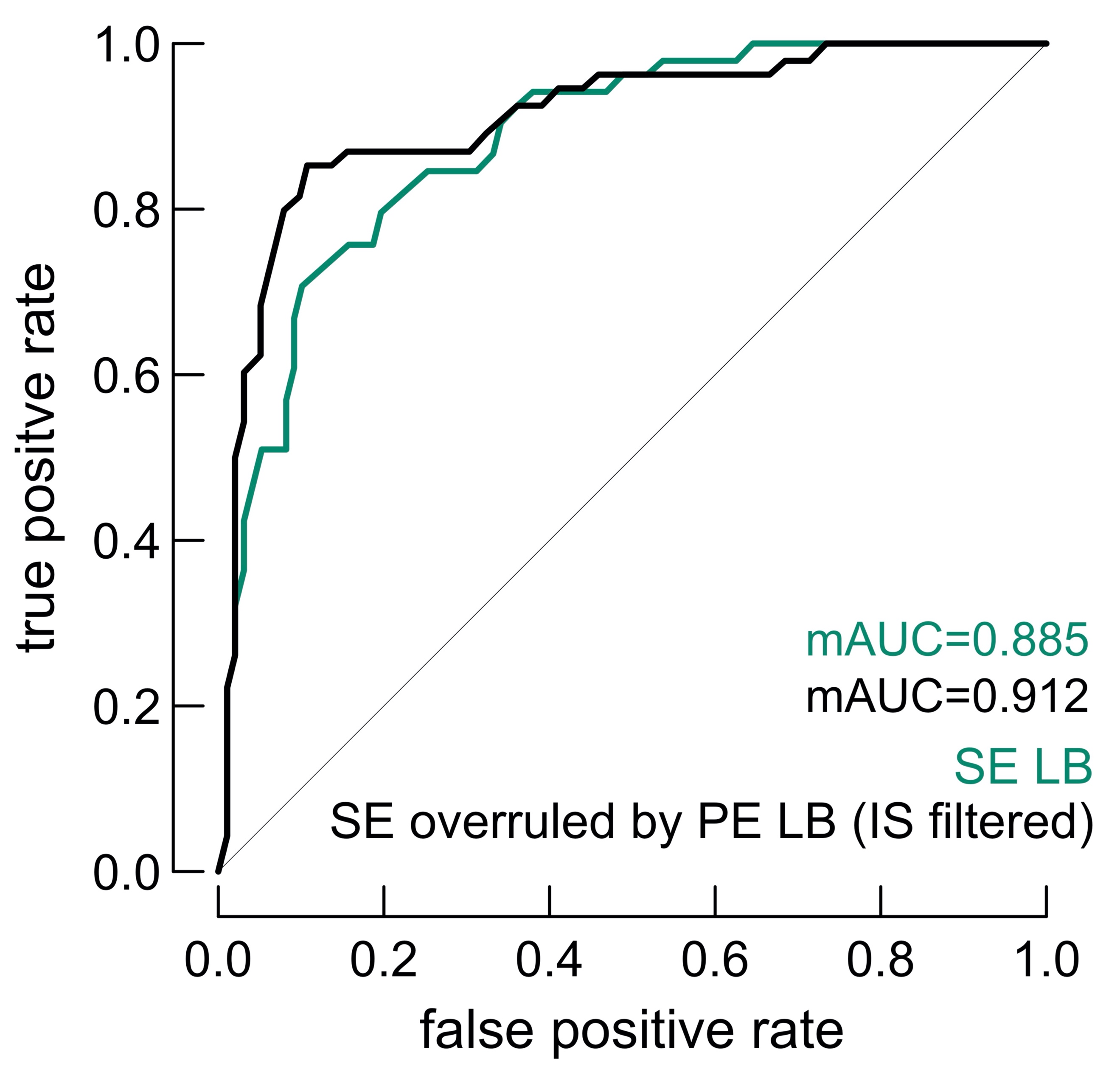


**Fig. S5** Performance of predicting histology by single-end (SE) versus paired-end (PE) sequencing. Receiver operating characteristic (ROC) analysis to evaluate predicting lung cancer histology, based on copy number profiles derived from liquid biopsies (LBs). The mean area under the curve (mAUC) was preferred as a performance statistic.


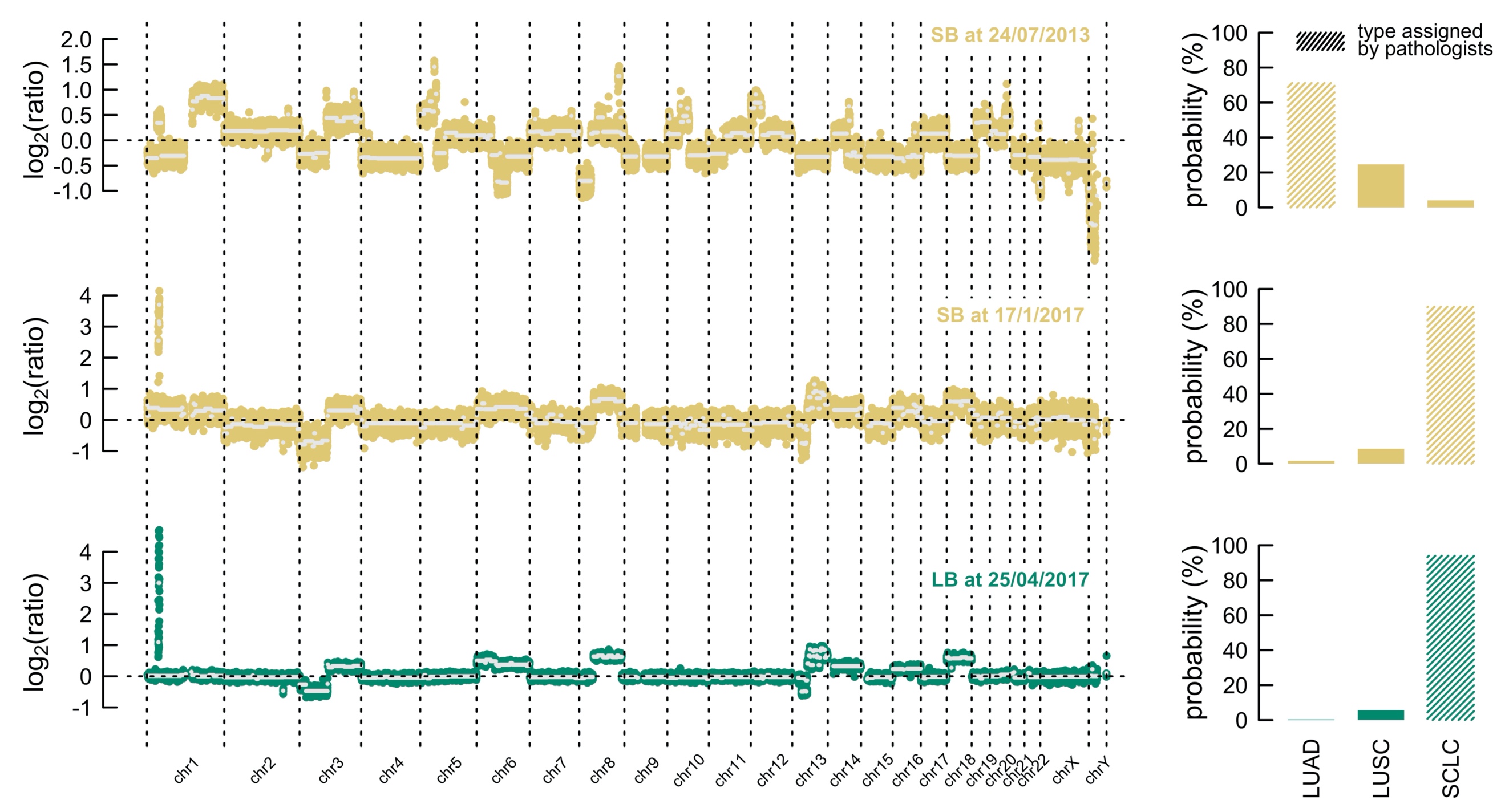


**Fig. S6**. Copy number profiles of a relapsed patient with small cell transformation/a second primary tumor. The top solid biopsy (SB) profile is not part of the overall cohort, as it was sequenced in addition. The SB (middle) and liquid biopsy (LB; bottom) below are taken at relapse and are part of the main cohort (patient 26). At the right-hand side, corresponding prediction probabilities are given by bar plots. This patient was treated in 2013 with sequential chemoradiotherapy after stage III LUAD was diagnosed in the lower left lobe, reaching complete remission. In September 2016, relapse at the same site was identified and accordingly treated with cisplatin pemetrexed. However, progression could not be halted after four cycles; after which a new biopsy indicated SCLC (January 2017). A LB could thus have anticipated SCLC sooner.
