# Supplementary material for: Shallow whole-genome sequencing of plasma cell-free DNA accurately differentiates small from non-small cell lung carcinoma
Source: Genome Med. 2020 Apr 21;12:35. doi: 10.1186/s13073-020-00735-4 (PMC7175544; doi:10.1186/s13073-020-00735-4)

[Copy number profile(s) of patient 1 1](#_Toc34144553)

[Copy number profile(s) of patient 2 1](#_Toc34144554)

[Copy number profile(s) of patient 3 1](#_Toc34144555)

[Copy number profile(s) of patient 4 2](#_Toc34144556)

[Copy number profile(s) of patient 5 2](#_Toc34144557)

[Copy number profile(s) of patient 6 2](#_Toc34144558)

[Copy number profile(s) of patient 7 3](#_Toc34144559)

[Copy number profile(s) of patient 8 3](#_Toc34144560)

[Copy number profile(s) of patient 9 4](#_Toc34144561)

[Copy number profile(s) of patient 10 4](#_Toc34144562)

[Copy number profile(s) of patient 11 5](#_Toc34144563)

[Copy number profile(s) of patient 12 5](#_Toc34144564)

[Copy number profile(s) of patient 13 6](#_Toc34144565)

[Copy number profile(s) of patient 14 6](#_Toc34144566)

[Copy number profile(s) of patient 15 6](#_Toc34144567)

[Copy number profile(s) of patient 16 7](#_Toc34144568)

[Copy number profile(s) of patient 17 7](#_Toc34144569)

[Copy number profile(s) of patient 18 8](#_Toc34144570)

[Copy number profile(s) of patient 19 8](#_Toc34144571)

[Copy number profile(s) of patient 20 8](#_Toc34144572)

[Copy number profile(s) of patient 21 9](#_Toc34144573)

[Copy number profile(s) of patient 22 9](#_Toc34144574)

[Copy number profile(s) of patient 23 10](#_Toc34144575)

[Copy number profile(s) of patient 24 10](#_Toc34144576)

[Copy number profile(s) of patient 25 11](#_Toc34144577)

[Copy number profile(s) of patient 26 11](#_Toc34144578)

[Copy number profile(s) of patient 27 12](#_Toc34144579)

[Copy number profile(s) of patient 28 12](#_Toc34144580)

[Copy number profile(s) of patient 29 12](#_Toc34144581)

[Copy number profile(s) of patient 30 13](#_Toc34144582)

[Copy number profile(s) of patient 31 13](#_Toc34144583)

[Copy number profile(s) of patient 32 14](#_Toc34144584)

[Copy number profile(s) of patient 33 14](#_Toc34144585)

[Copy number profile(s) of patient 34 14](#_Toc34144586)

[Copy number profile(s) of patient 35 15](#_Toc34144587)

[Copy number profile(s) of patient 36 15](#_Toc34144588)

[Copy number profile(s) of patient 37 15](#_Toc34144589)

[Copy number profile(s) of patient 38 16](#_Toc34144590)

[Copy number profile(s) of patient 39 16](#_Toc34144591)

[Copy number profile(s) of patient 40 17](#_Toc34144592)

[Copy number profile(s) of patient 41 17](#_Toc34144593)

[Copy number profile(s) of patient 42 17](#_Toc34144594)

[Copy number profile(s) of patient 43 18](#_Toc34144595)

[Copy number profile(s) of patient 44 18](#_Toc34144596)

[Copy number profile(s) of patient 45 18](#_Toc34144597)

[Copy number profile(s) of patient 46 19](#_Toc34144598)

[Copy number profile(s) of patient 47 19](#_Toc34144599)

[Copy number profile(s) of patient 48 20](#_Toc34144600)

[Copy number profile(s) of patient 49 20](#_Toc34144601)

[Copy number profile(s) of patient 50 21](#_Toc34144602)

[Copy number profile(s) of patient 51 21](#_Toc34144603)

# Copy number profile(s) of patient 1


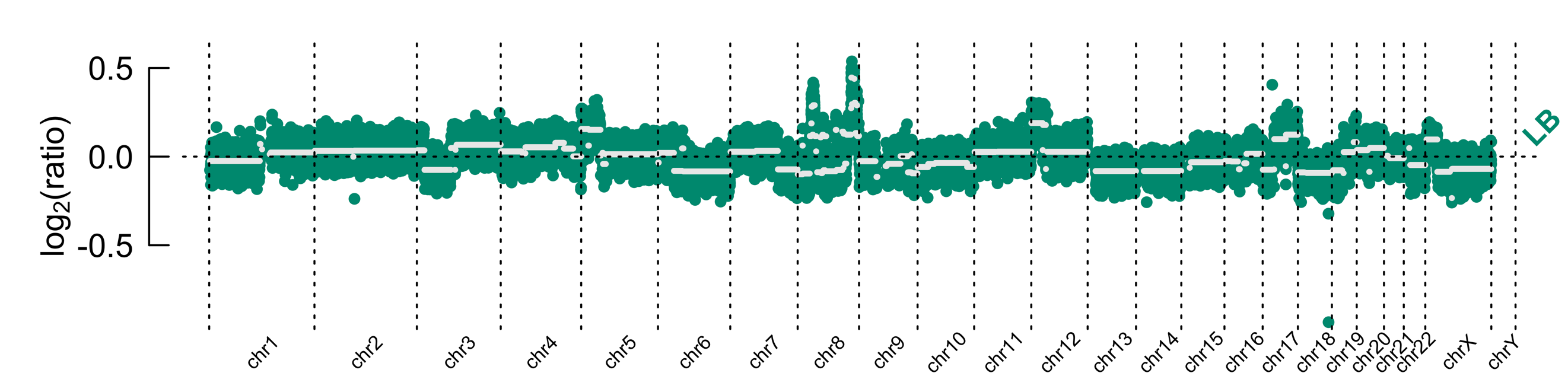


# Copy number profile(s) of patient 2


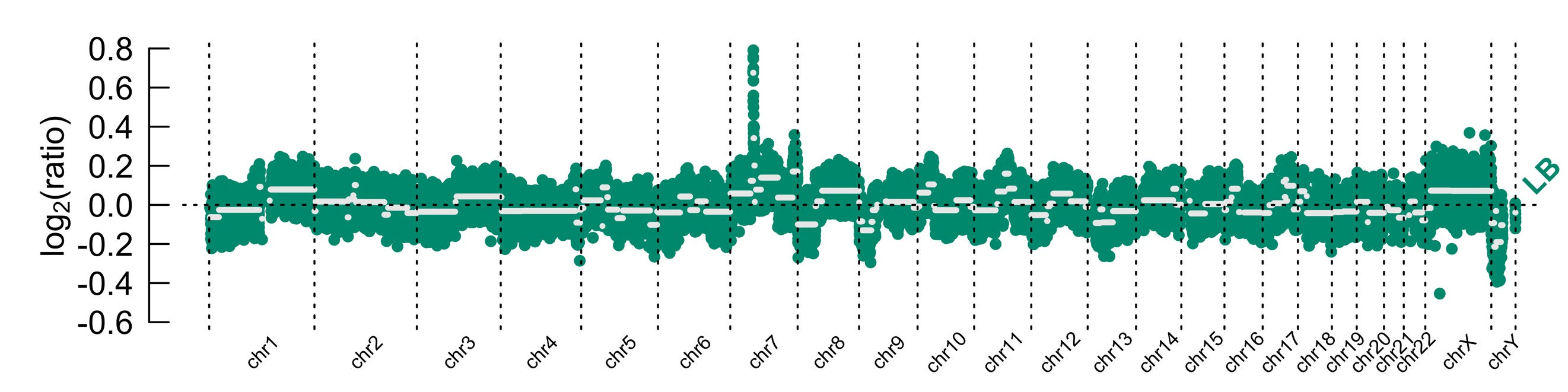


# Copy number profile(s) of patient 3


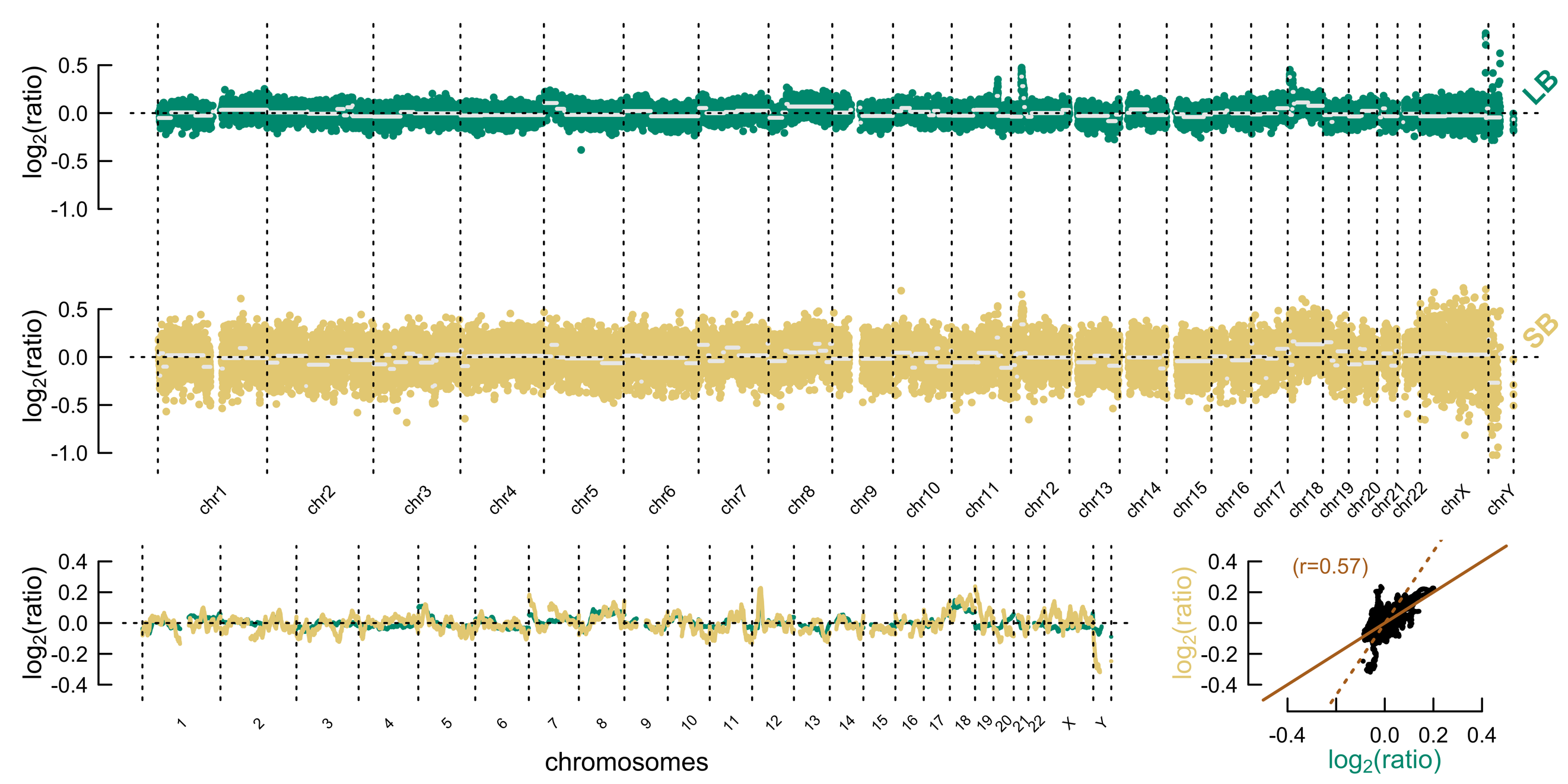


# Copy number profile(s) of patient 4


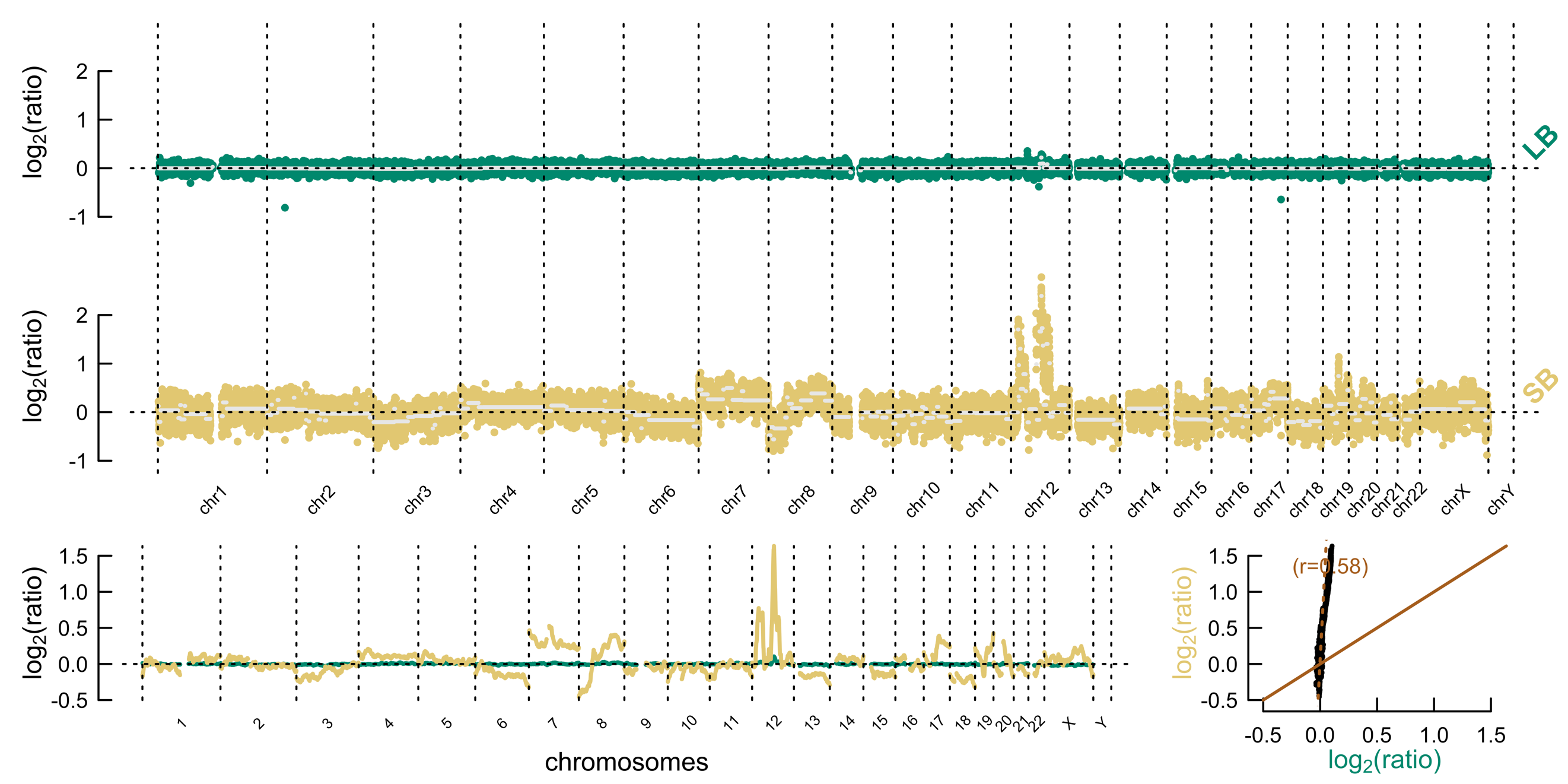


# Copy number profile(s) of patient 5


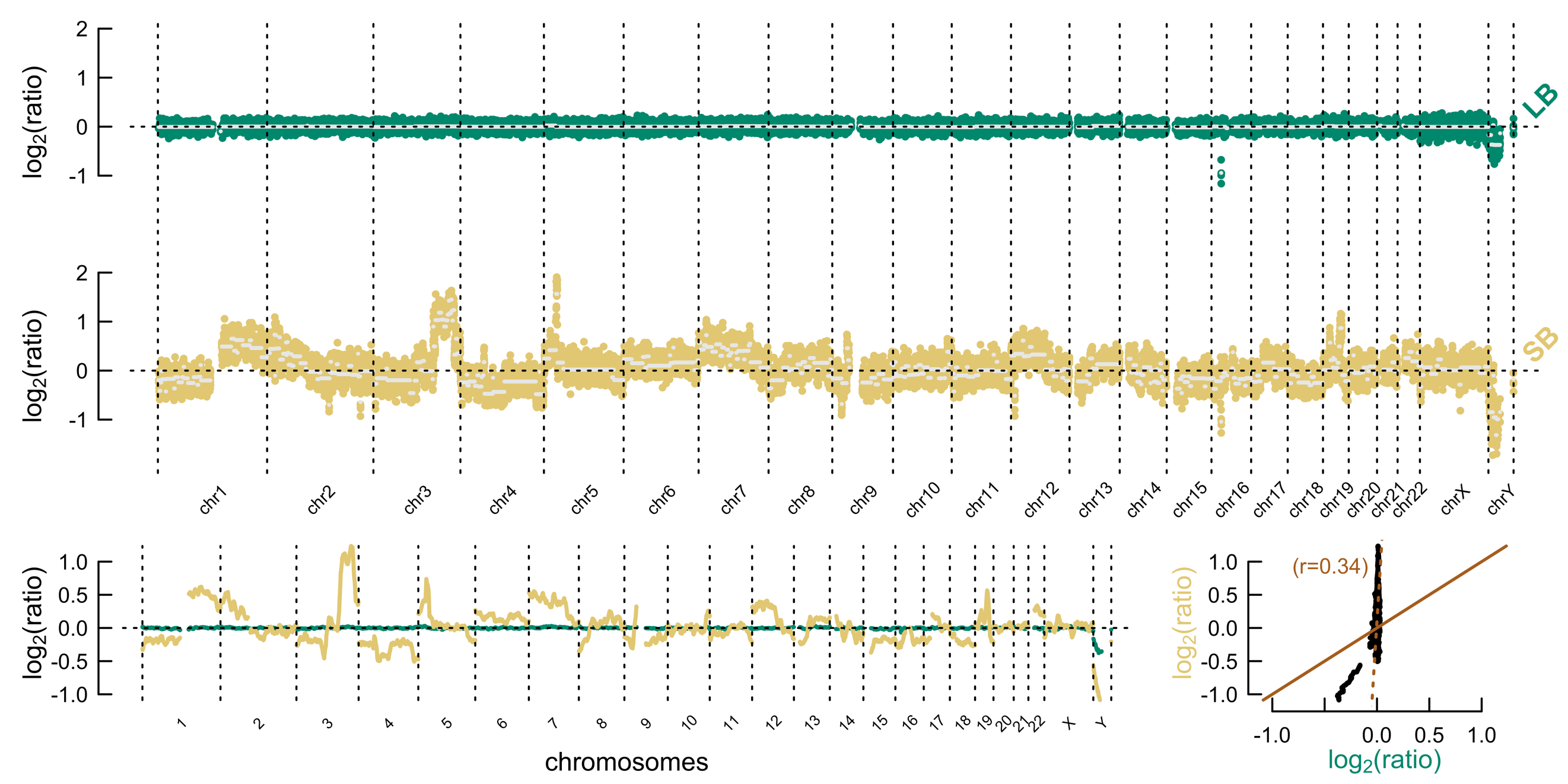


# Copy number profile(s) of patient 6


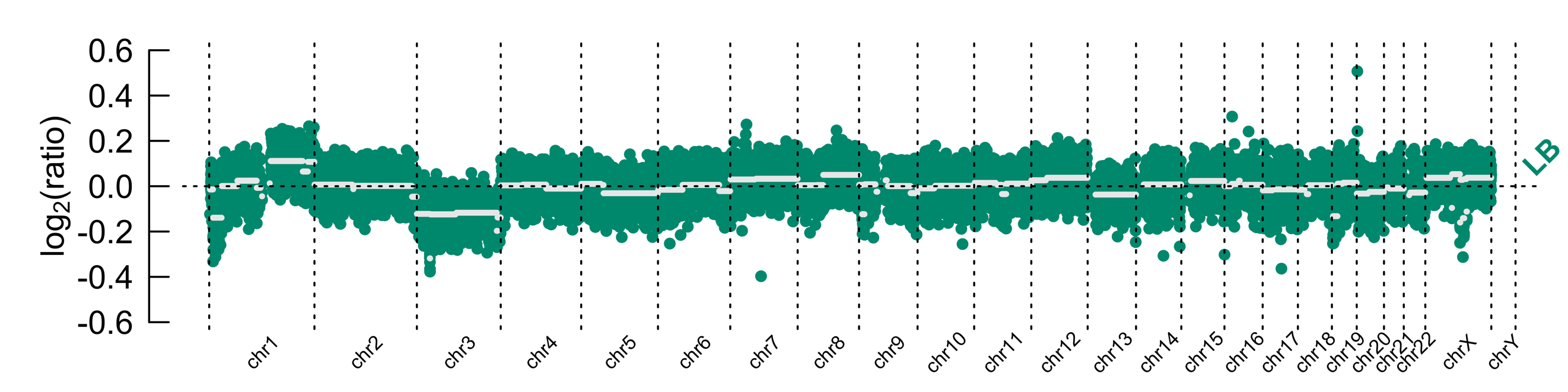


# Copy number profile(s) of patient 7


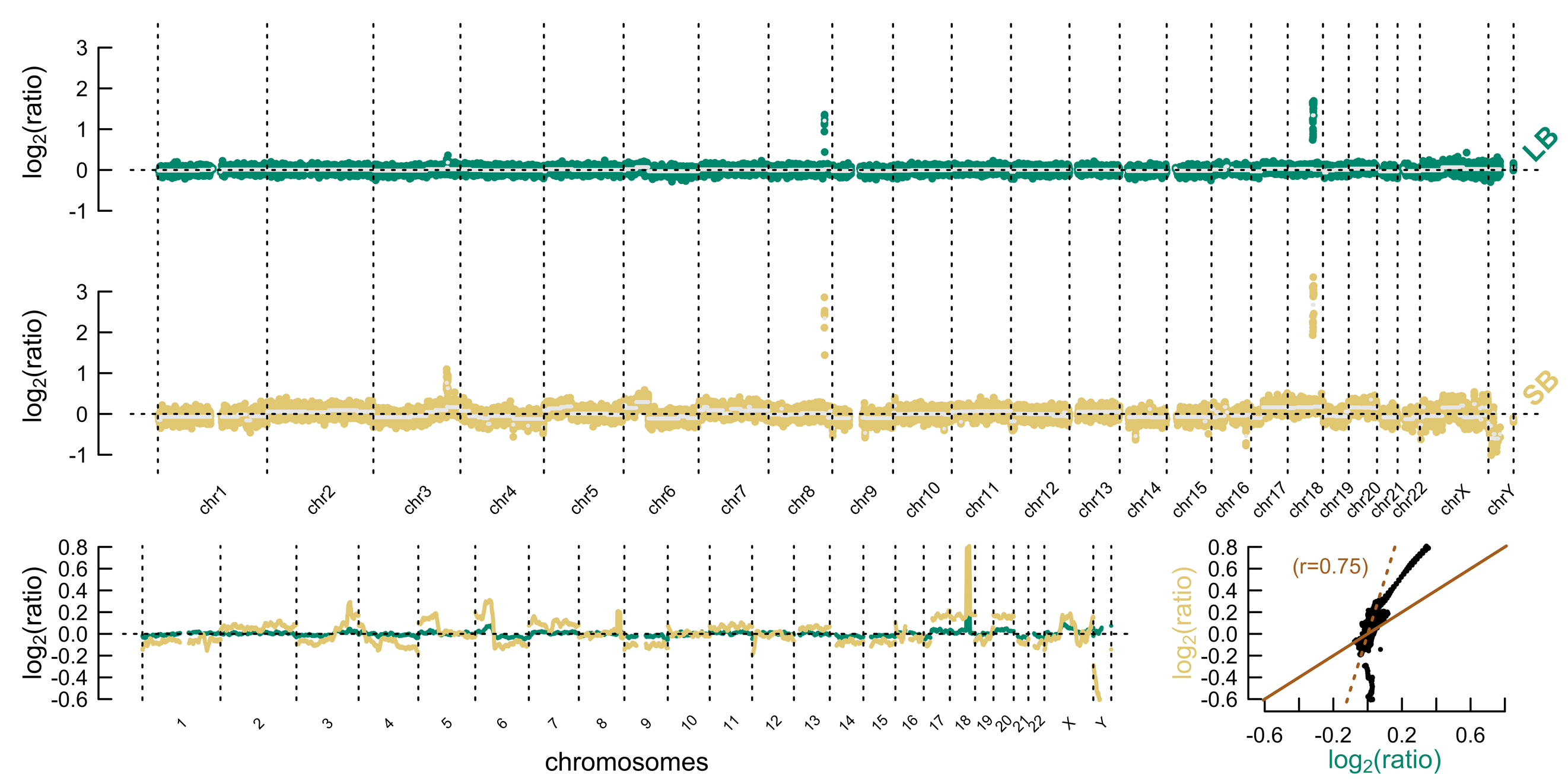


# Copy number profile(s) of patient 8


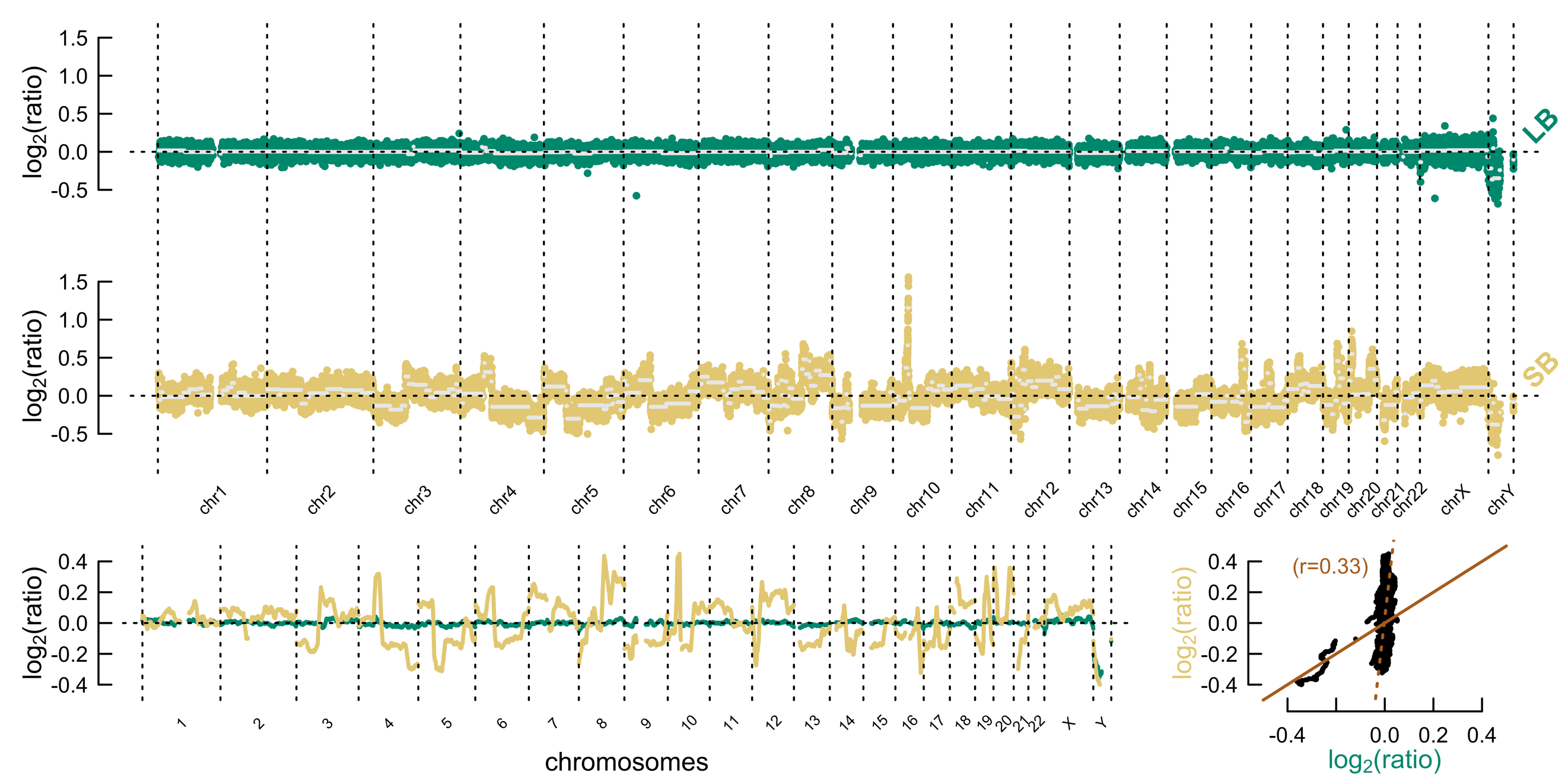


# Copy number profile(s) of patient 9


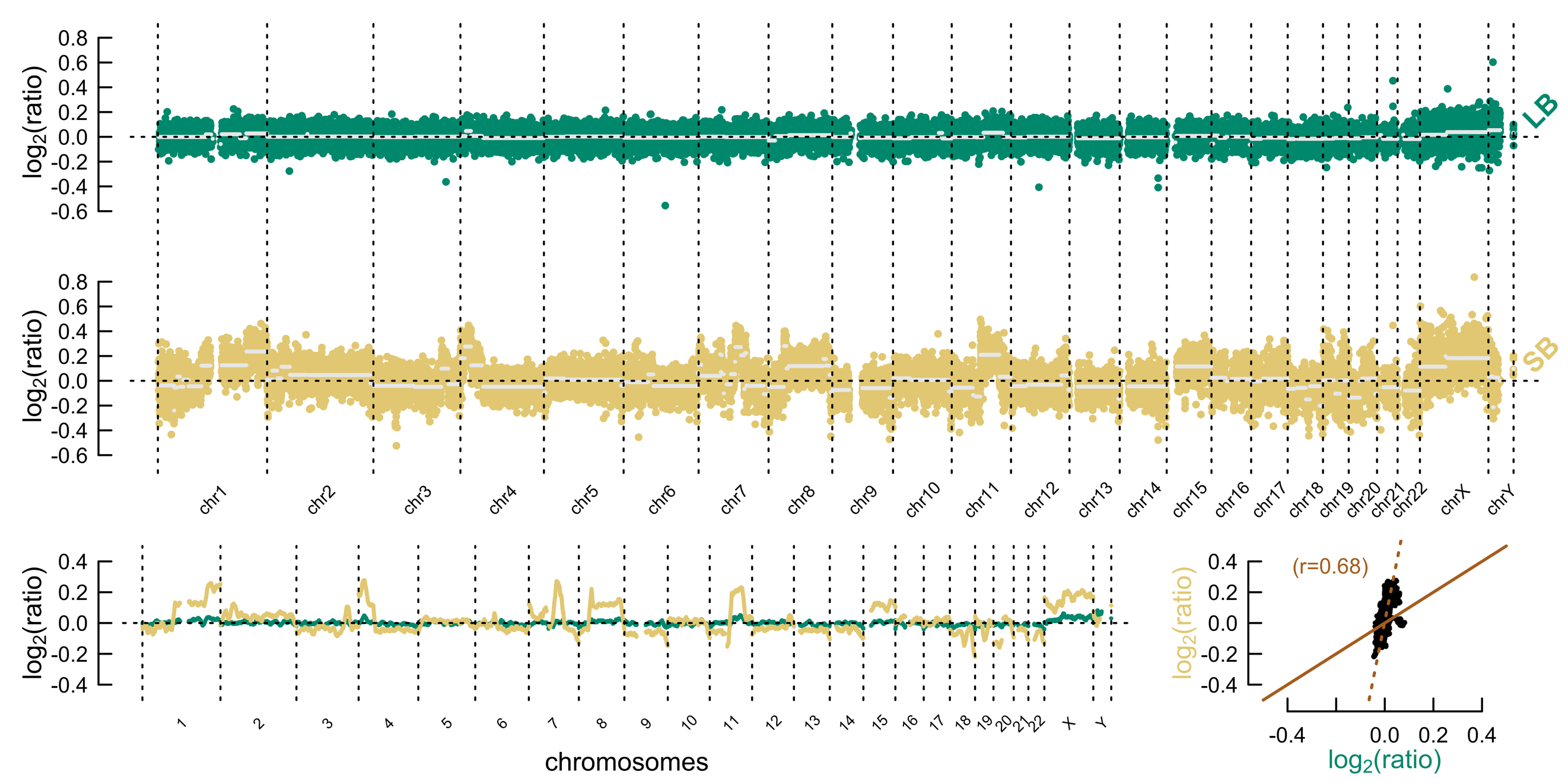


# Copy number profile(s) of patient 10


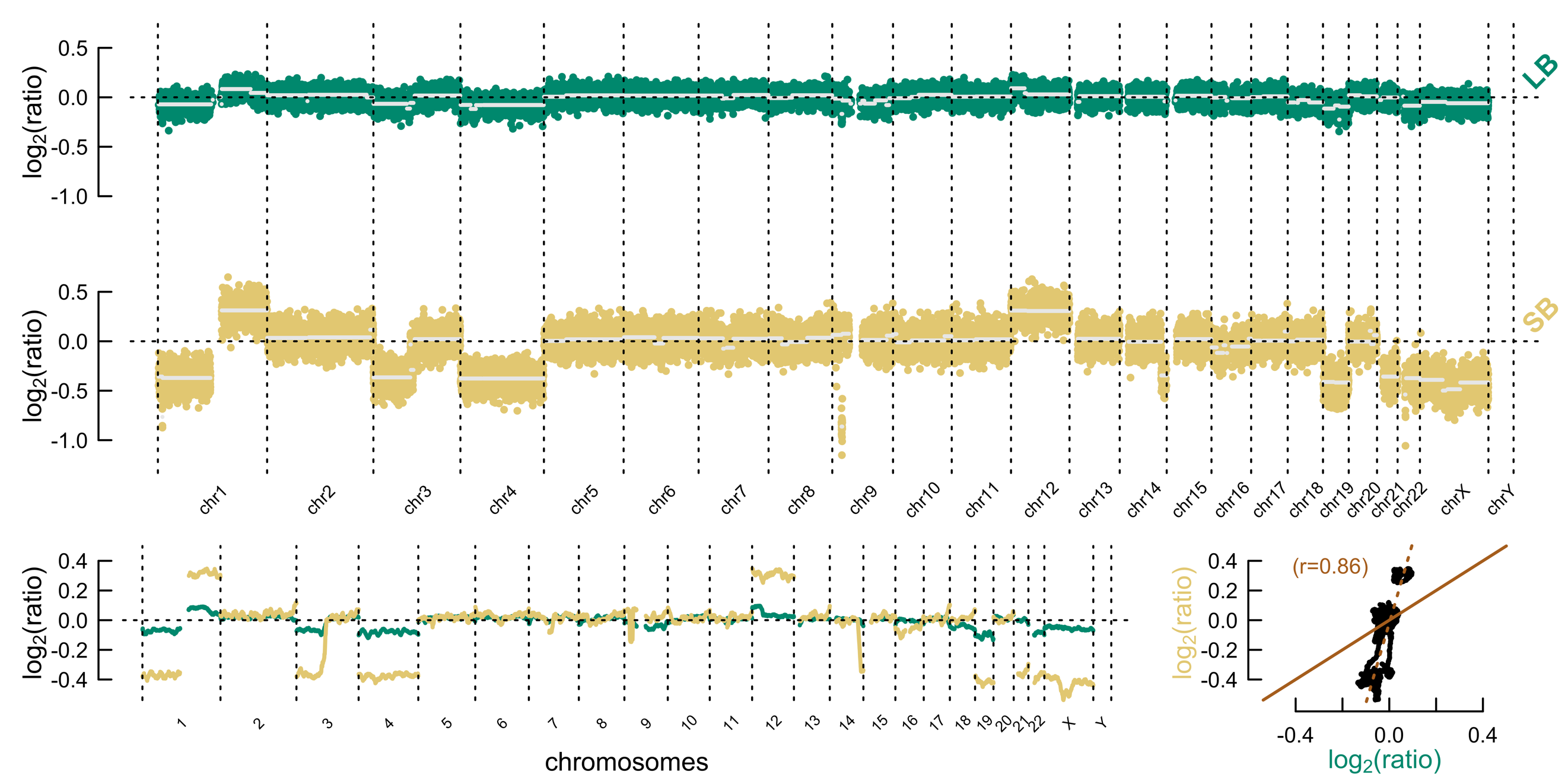


# Copy number profile(s) of patient 11


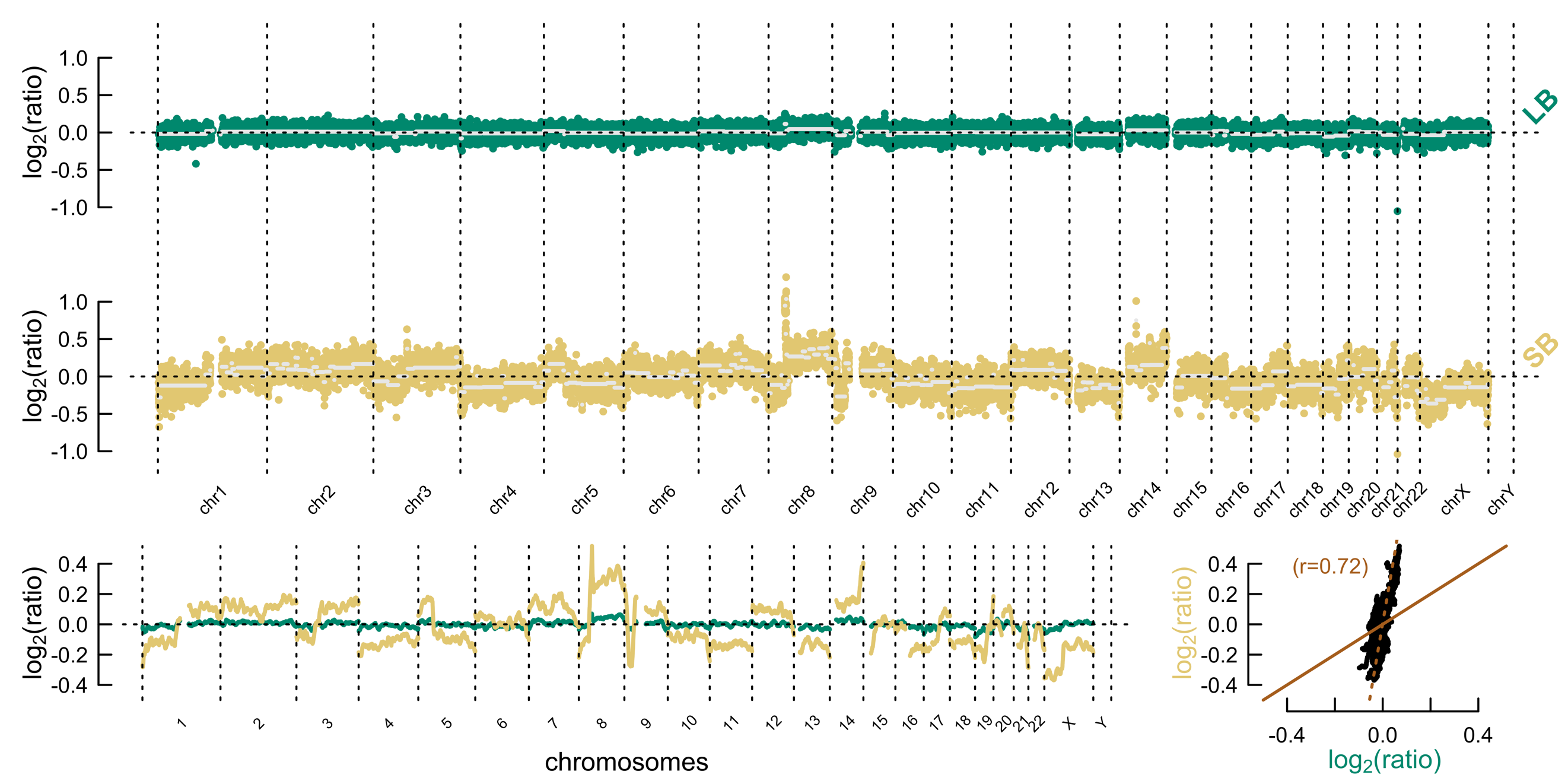


# Copy number profile(s) of patient 12


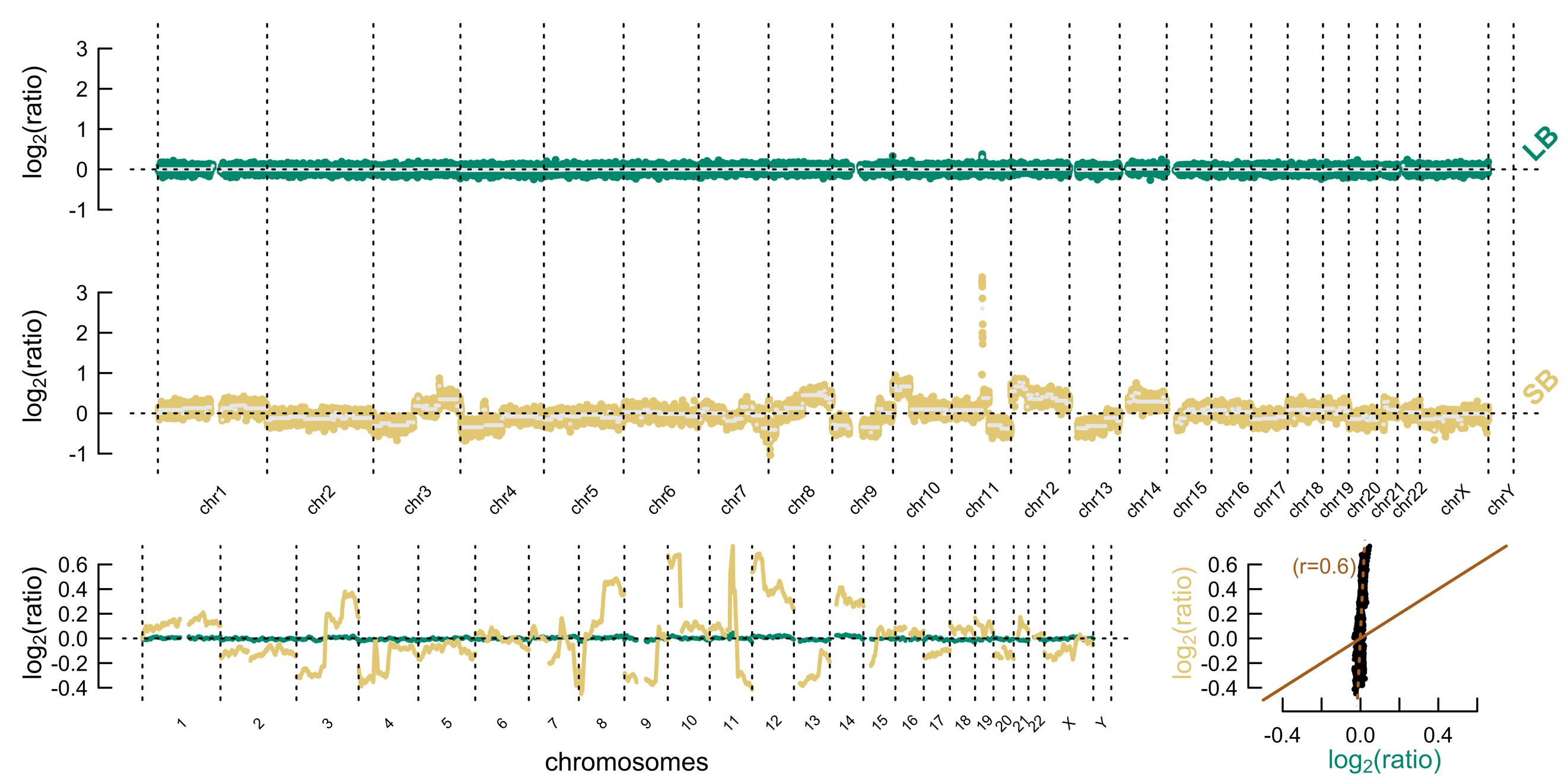


# Copy number profile(s) of patient 13


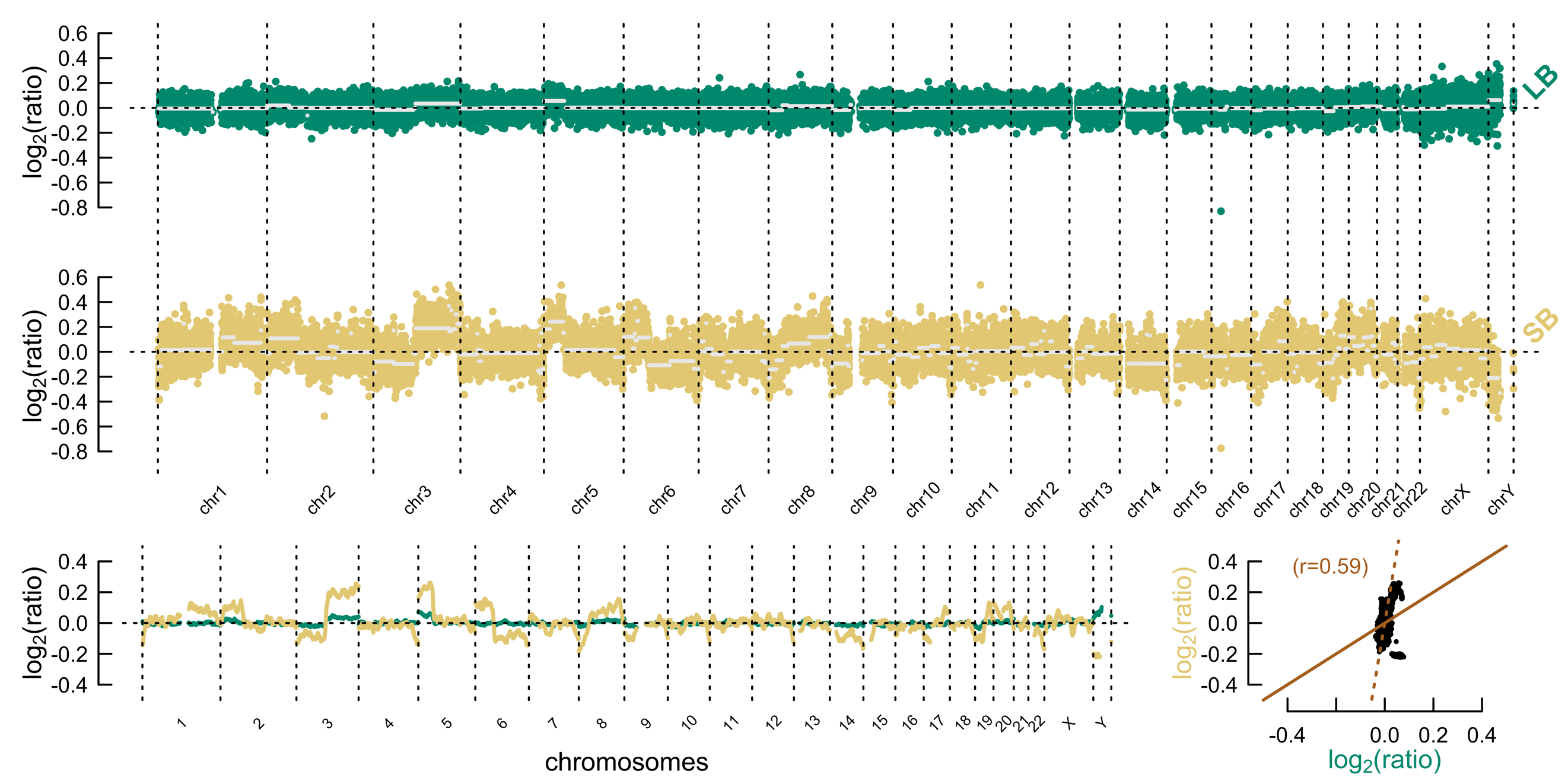


# Copy number profile(s) of patient 14


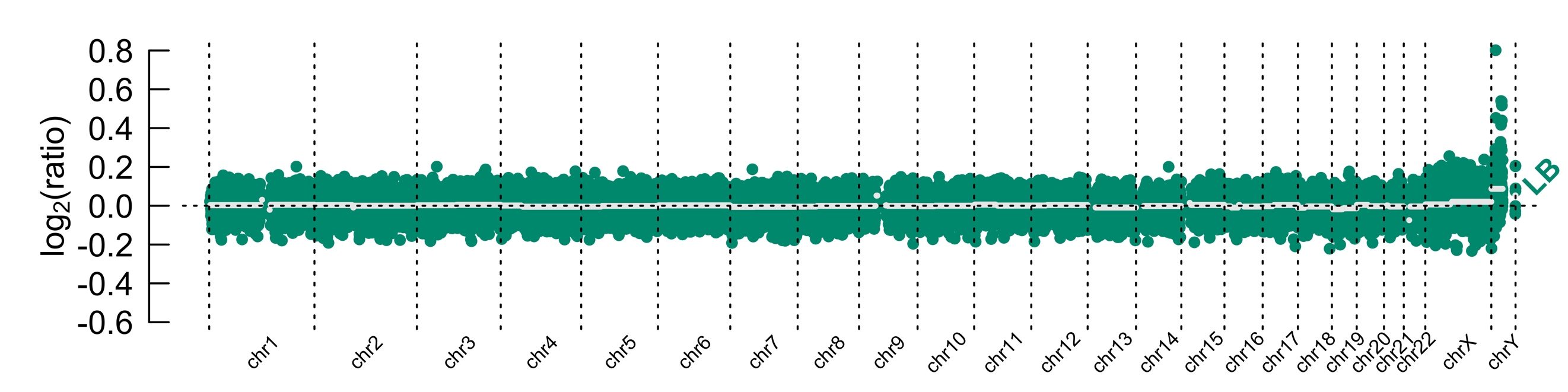


# Copy number profile(s) of patient 15


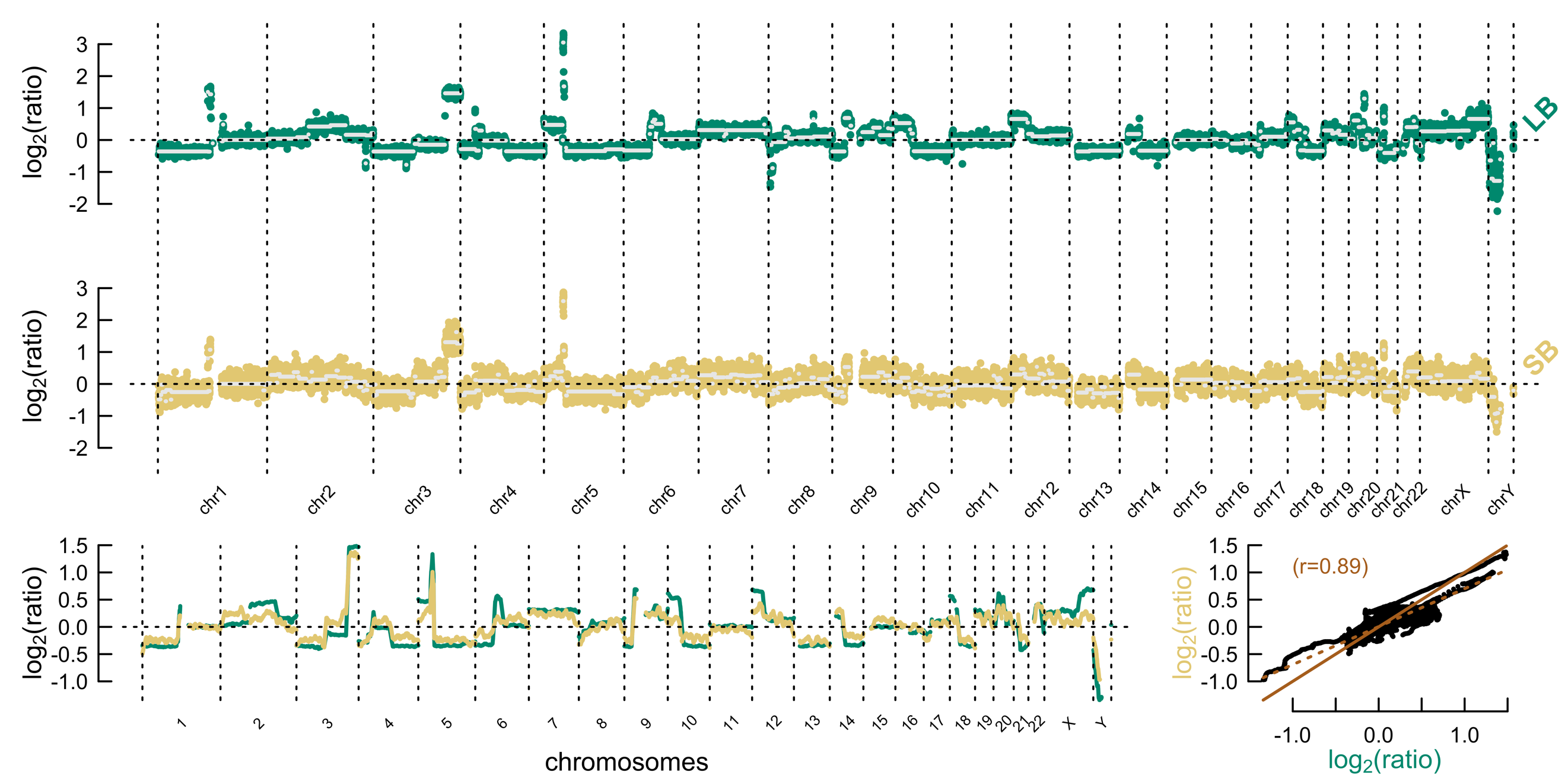


# Copy number profile(s) of patient 16


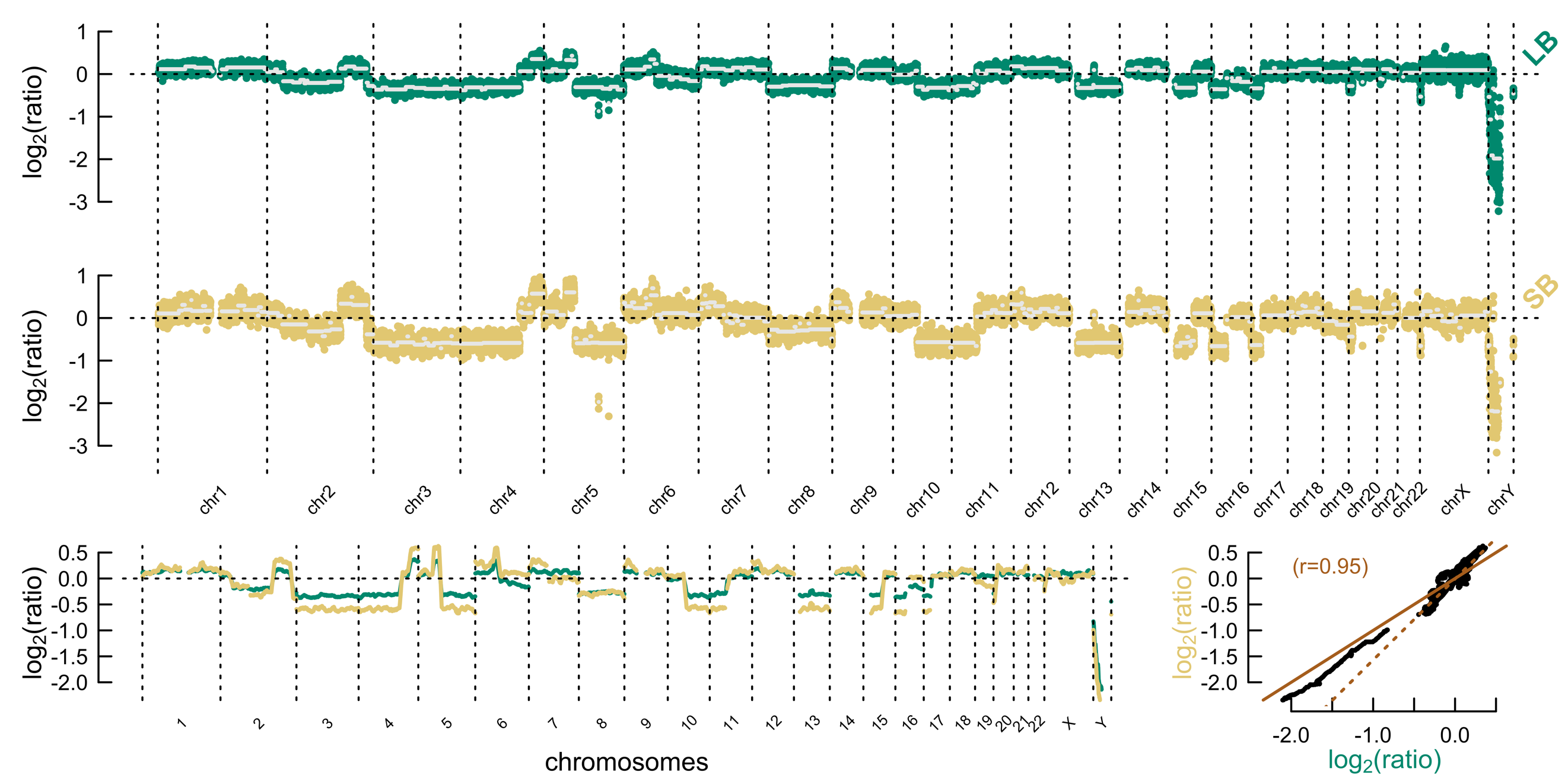


# Copy number profile(s) of patient 17


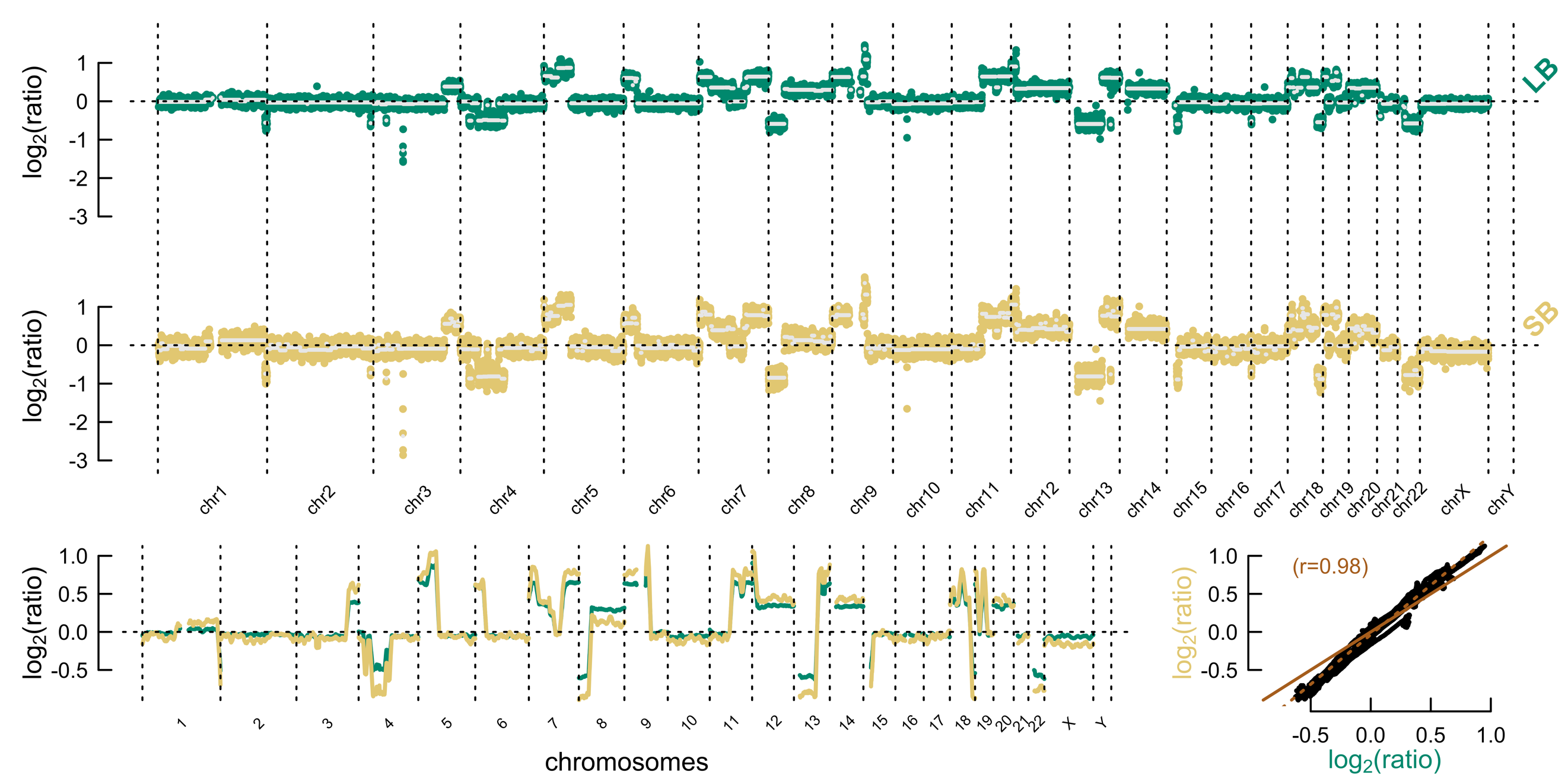


# Copy number profile(s) of patient 18


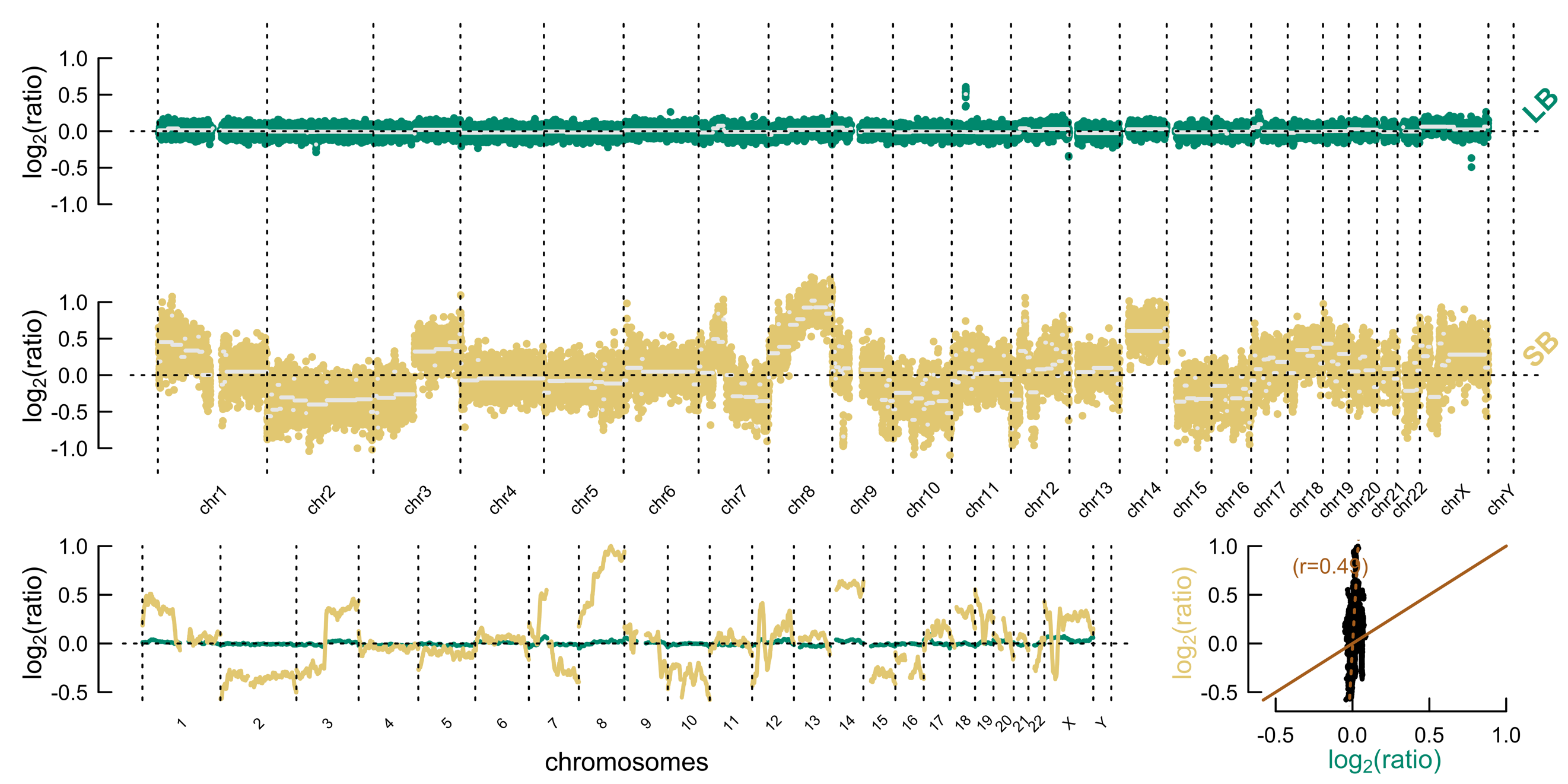


# Copy number profile(s) of patient 19


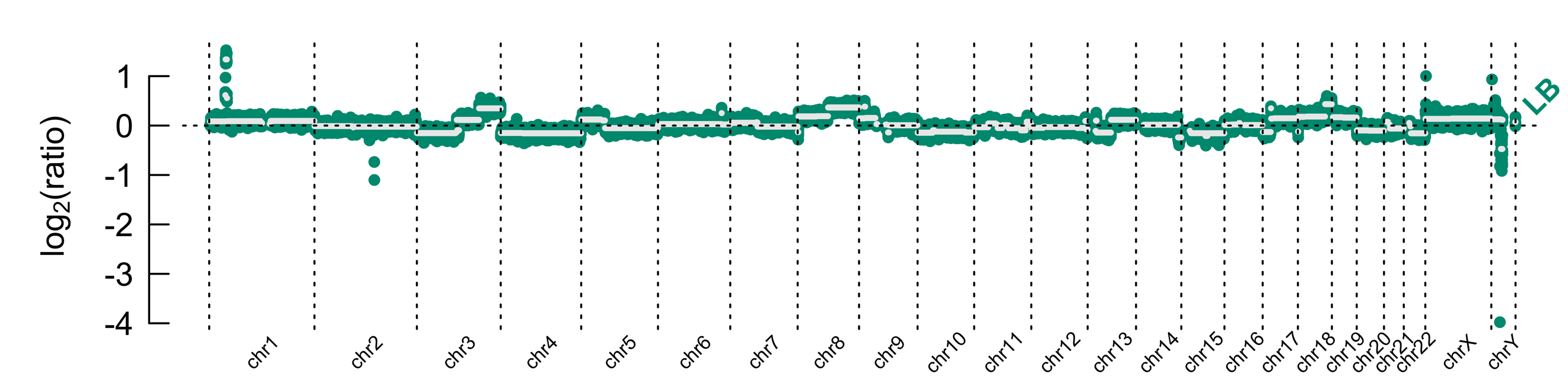


# Copy number profile(s) of patient 20


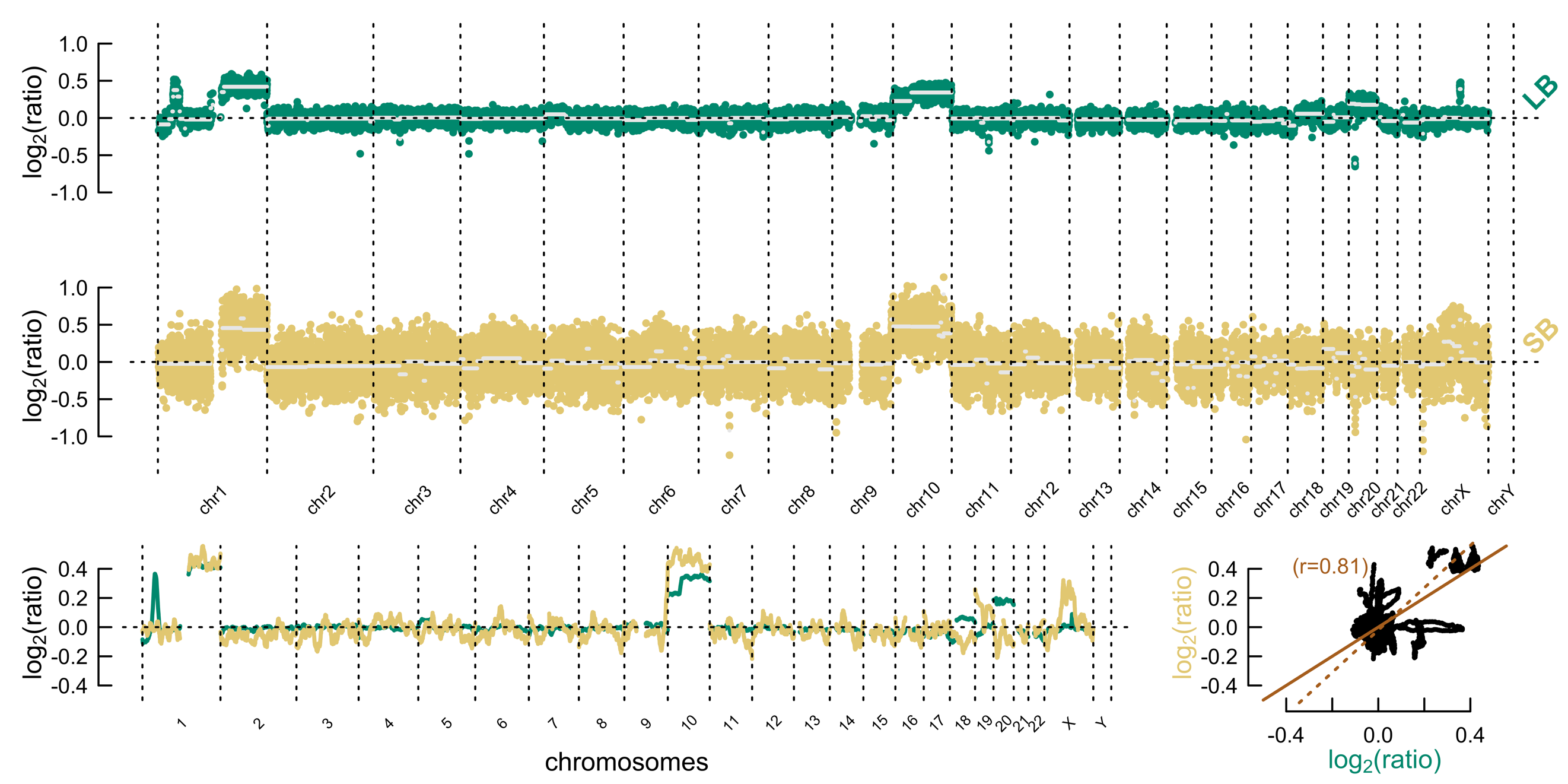


# Copy number profile(s) of patient 21


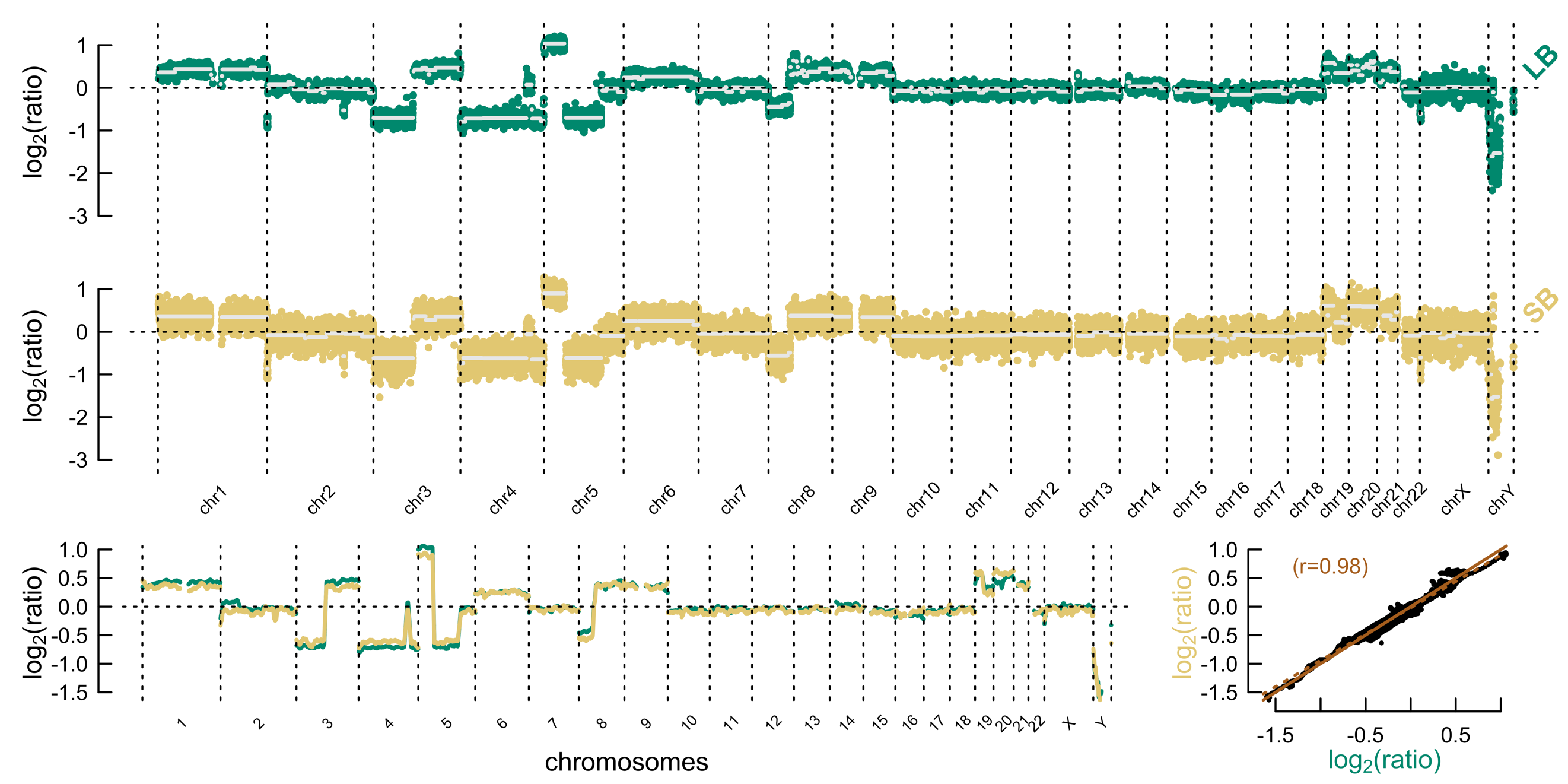


# Copy number profile(s) of patient 22


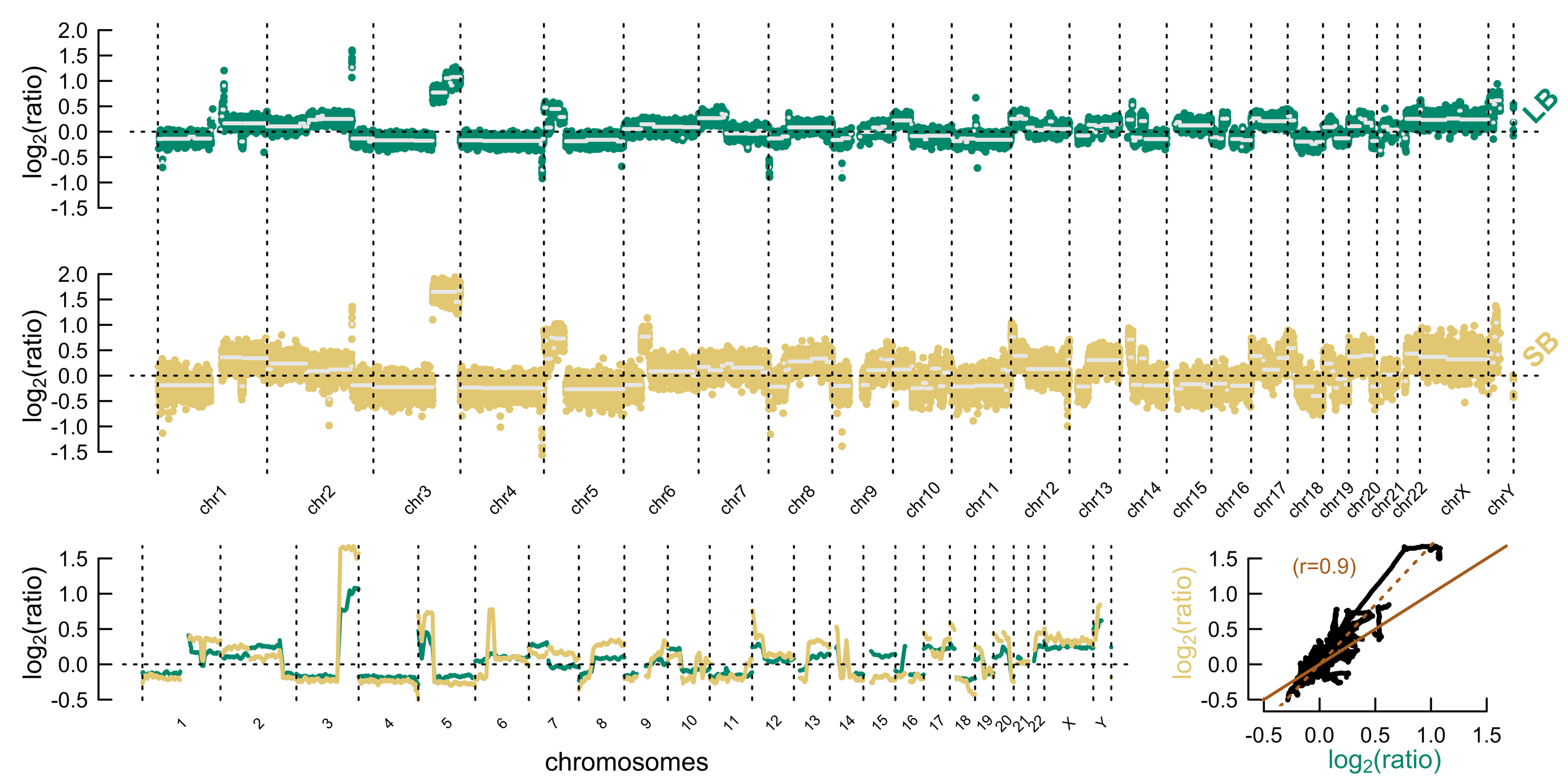


# Copy number profile(s) of patient 23


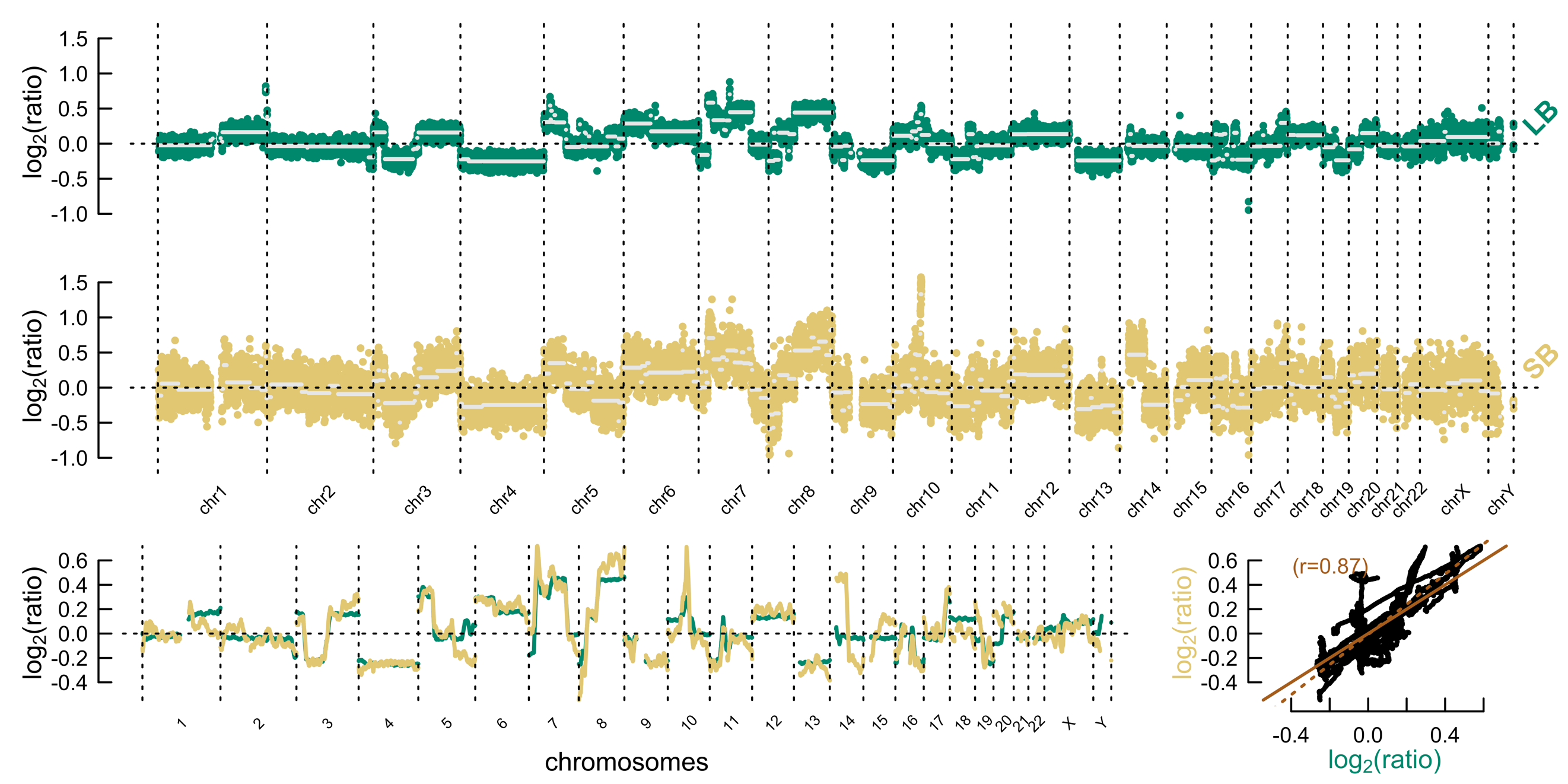


# Copy number profile(s) of patient 24


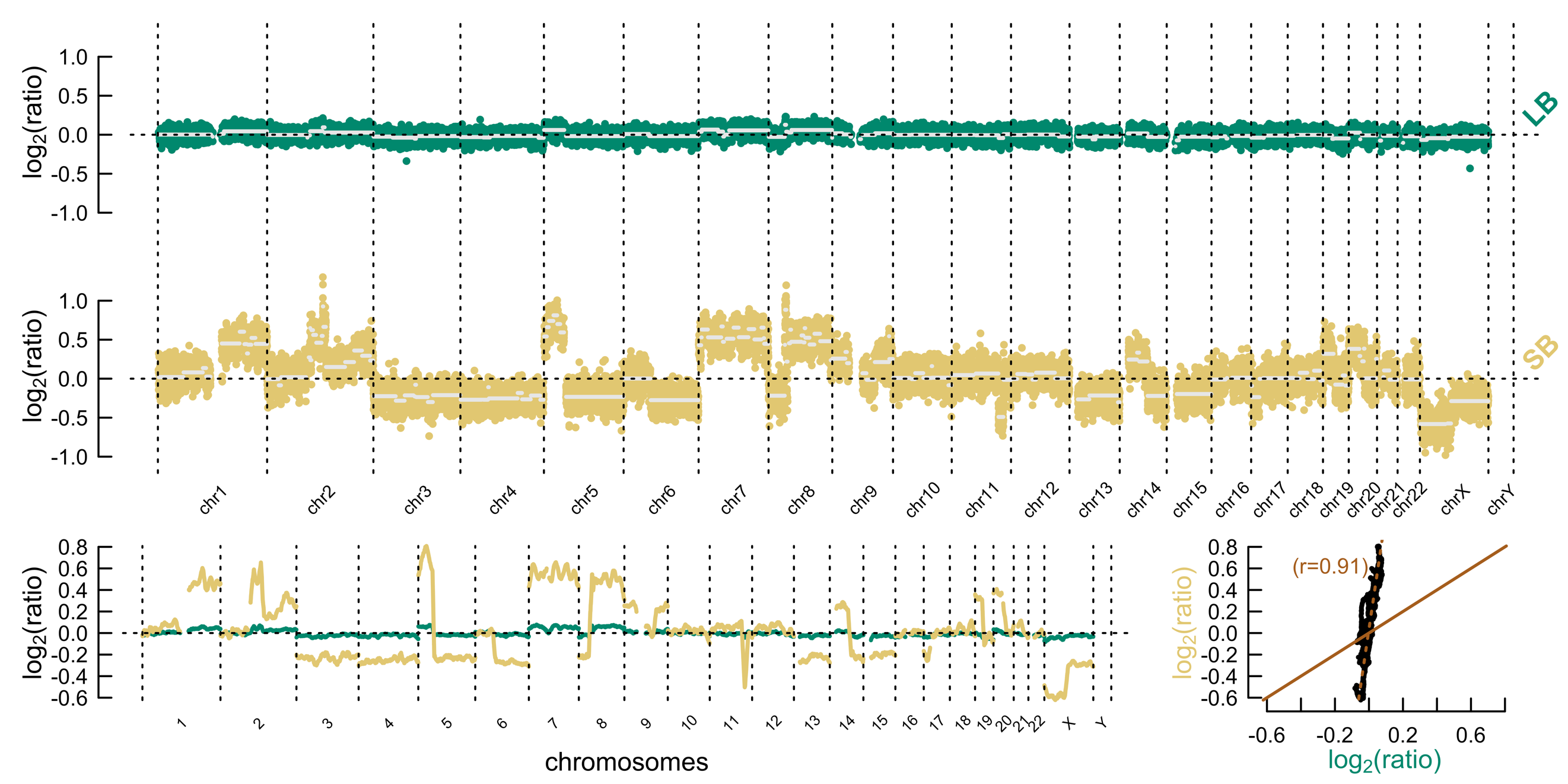


# Copy number profile(s) of patient 25


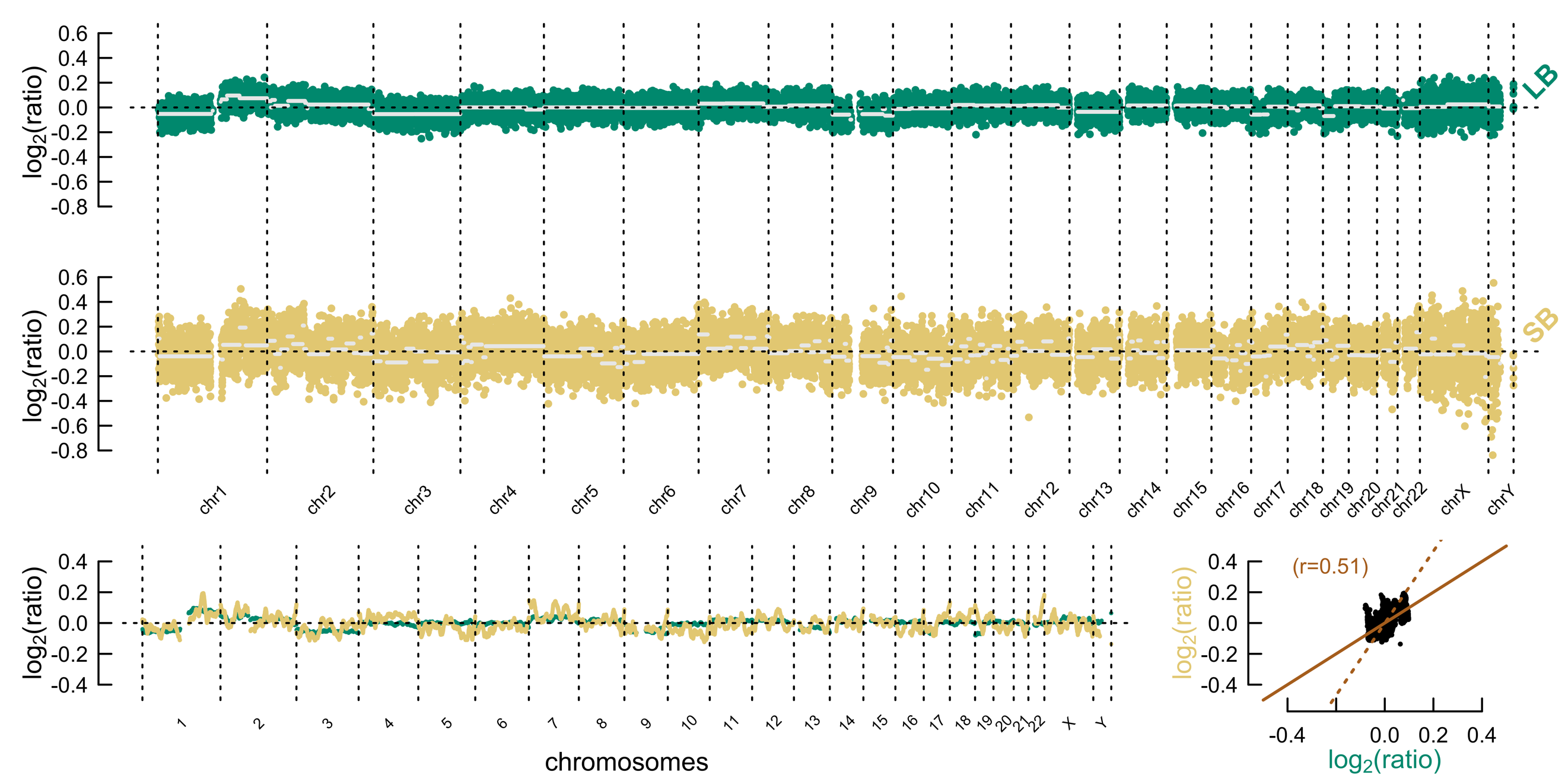


# Copy number profile(s) of patient 26


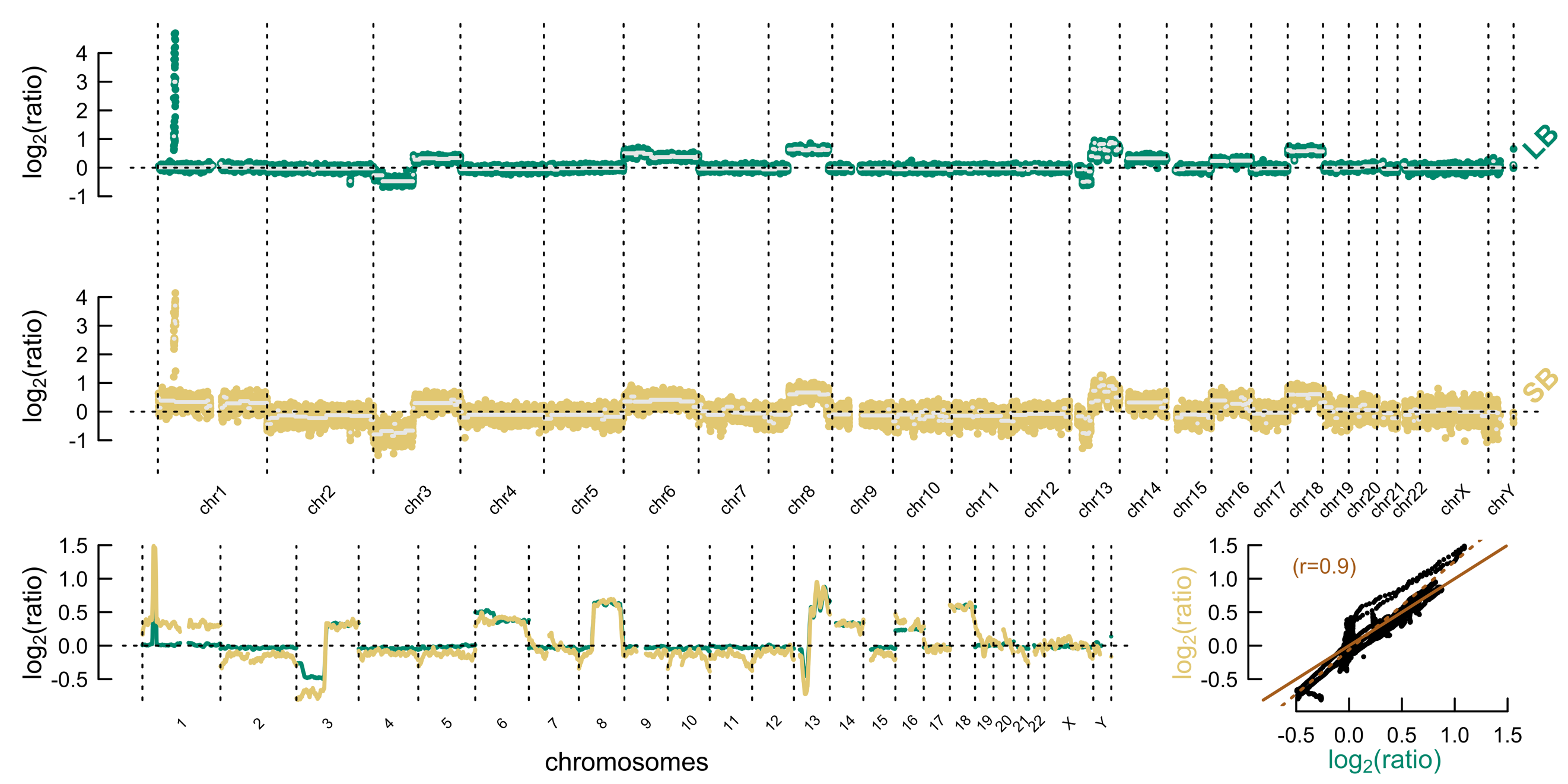


# Copy number profile(s) of patient 27


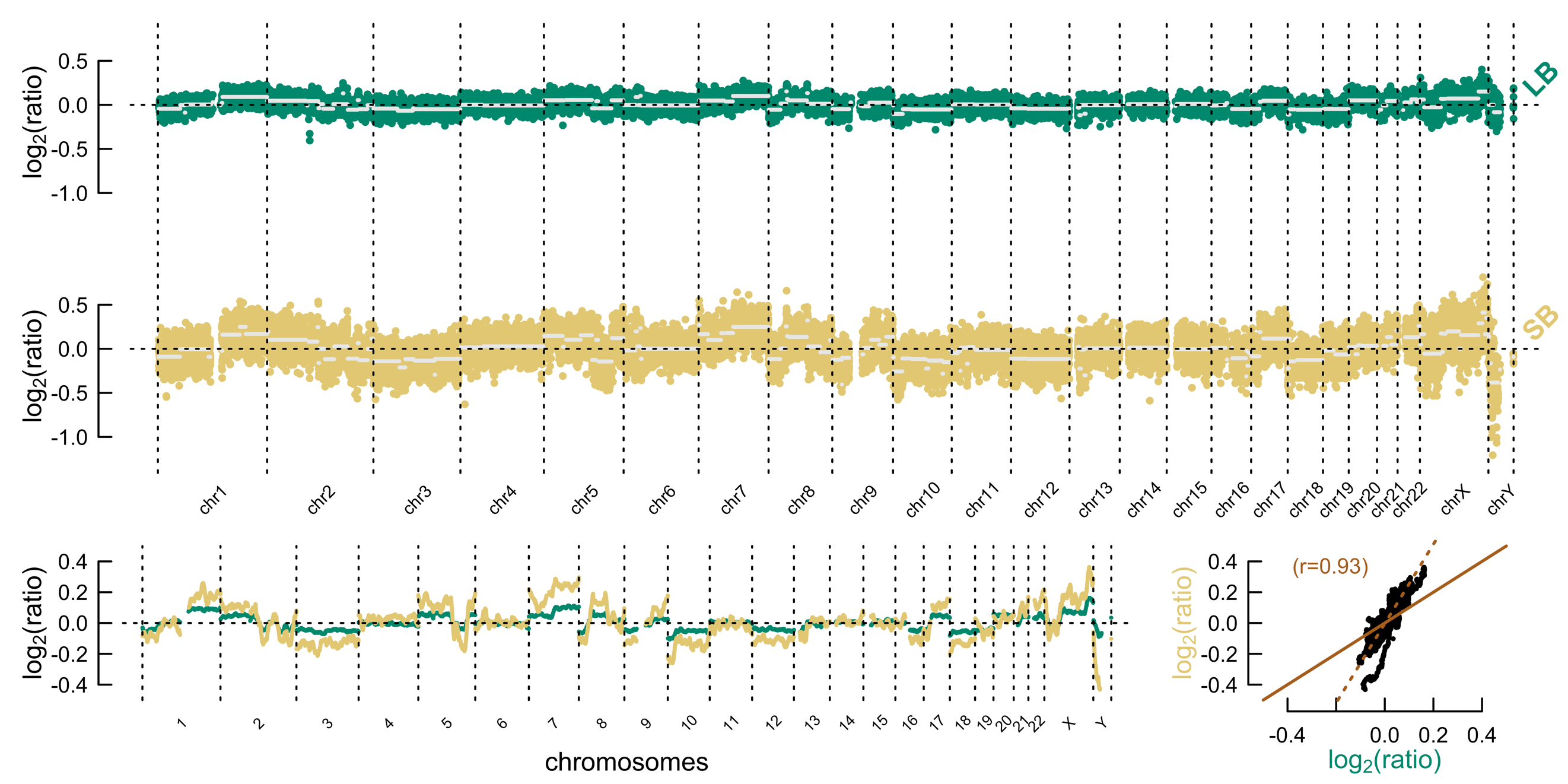


# Copy number profile(s) of patient 28


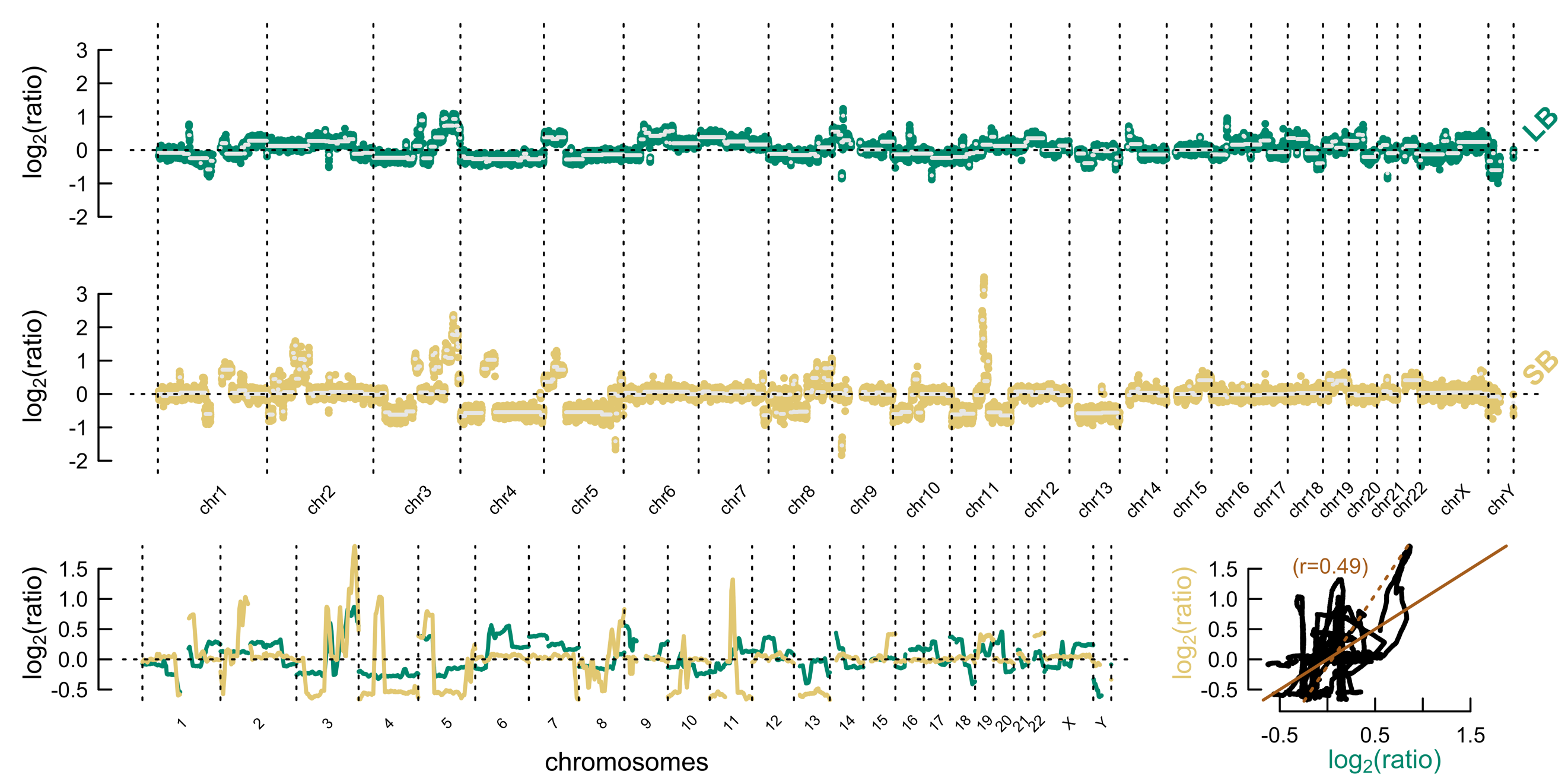


# Copy number profile(s) of patient 29


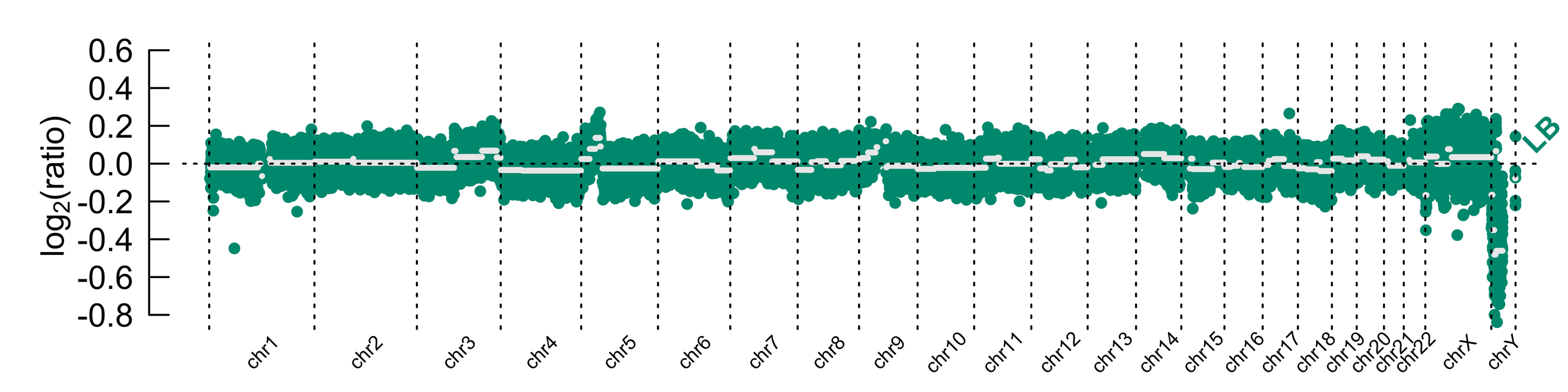


# Copy number profile(s) of patient 30


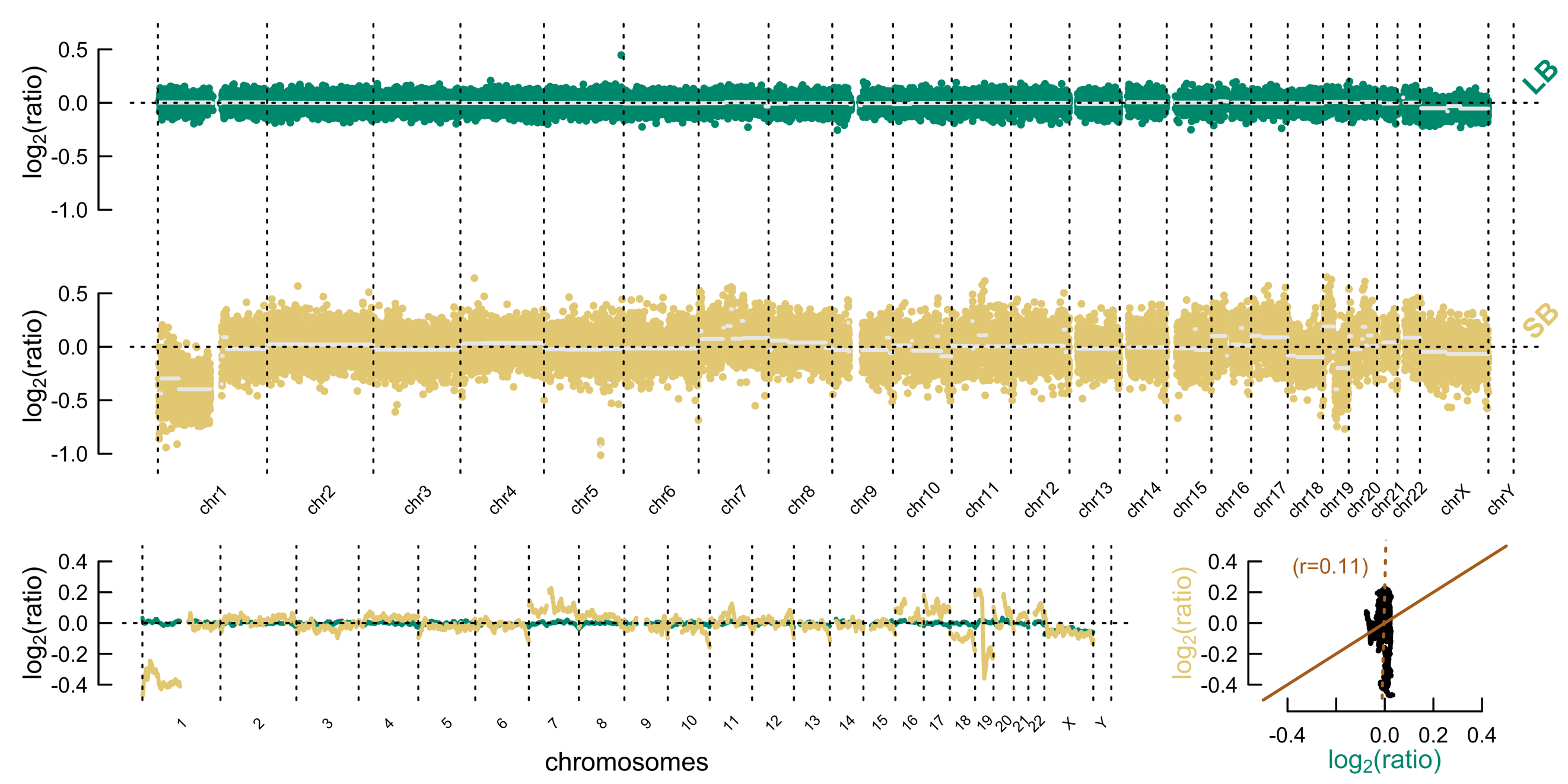


# Copy number profile(s) of patient 31


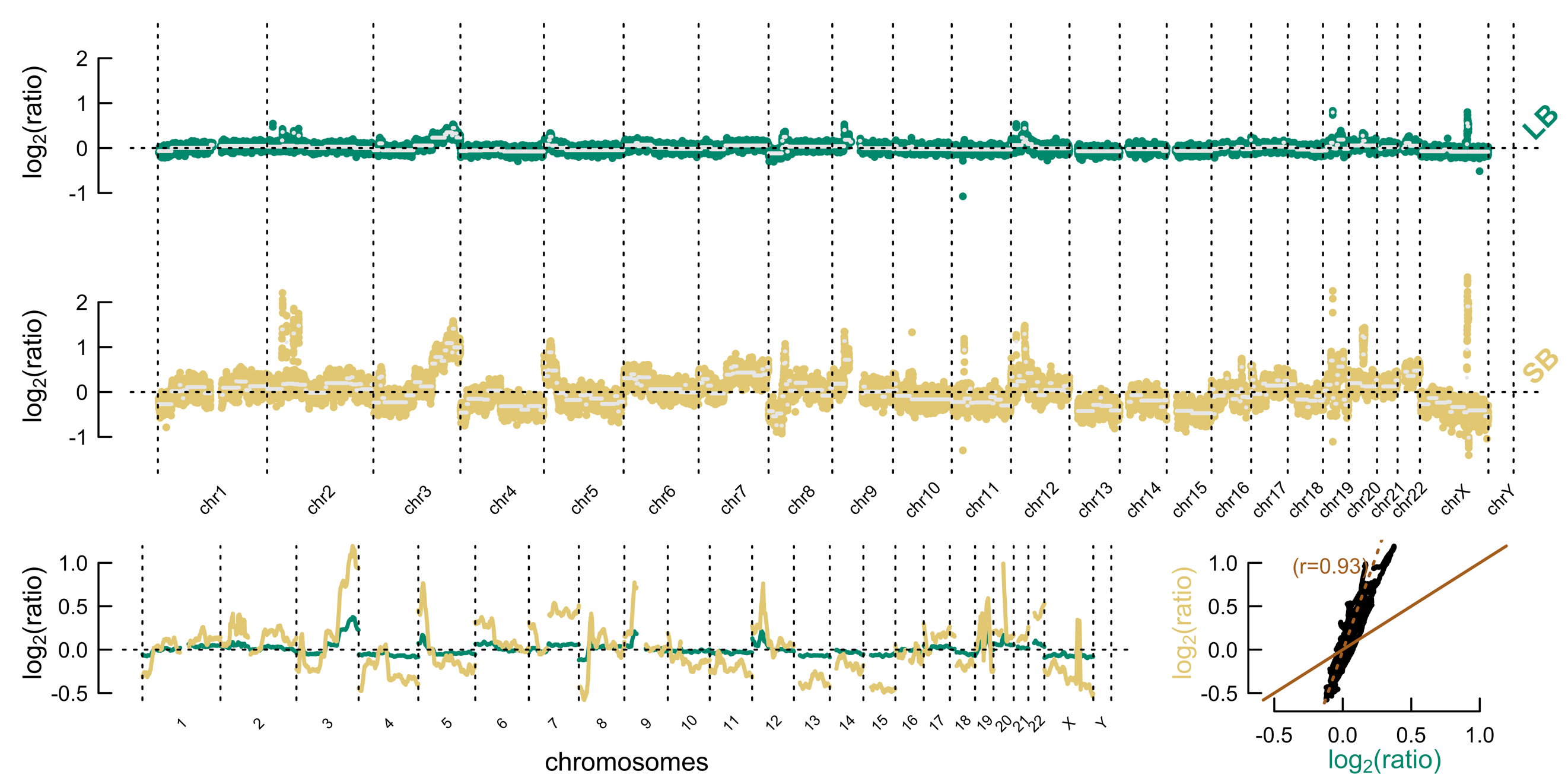


# Copy number profile(s) of patient 32


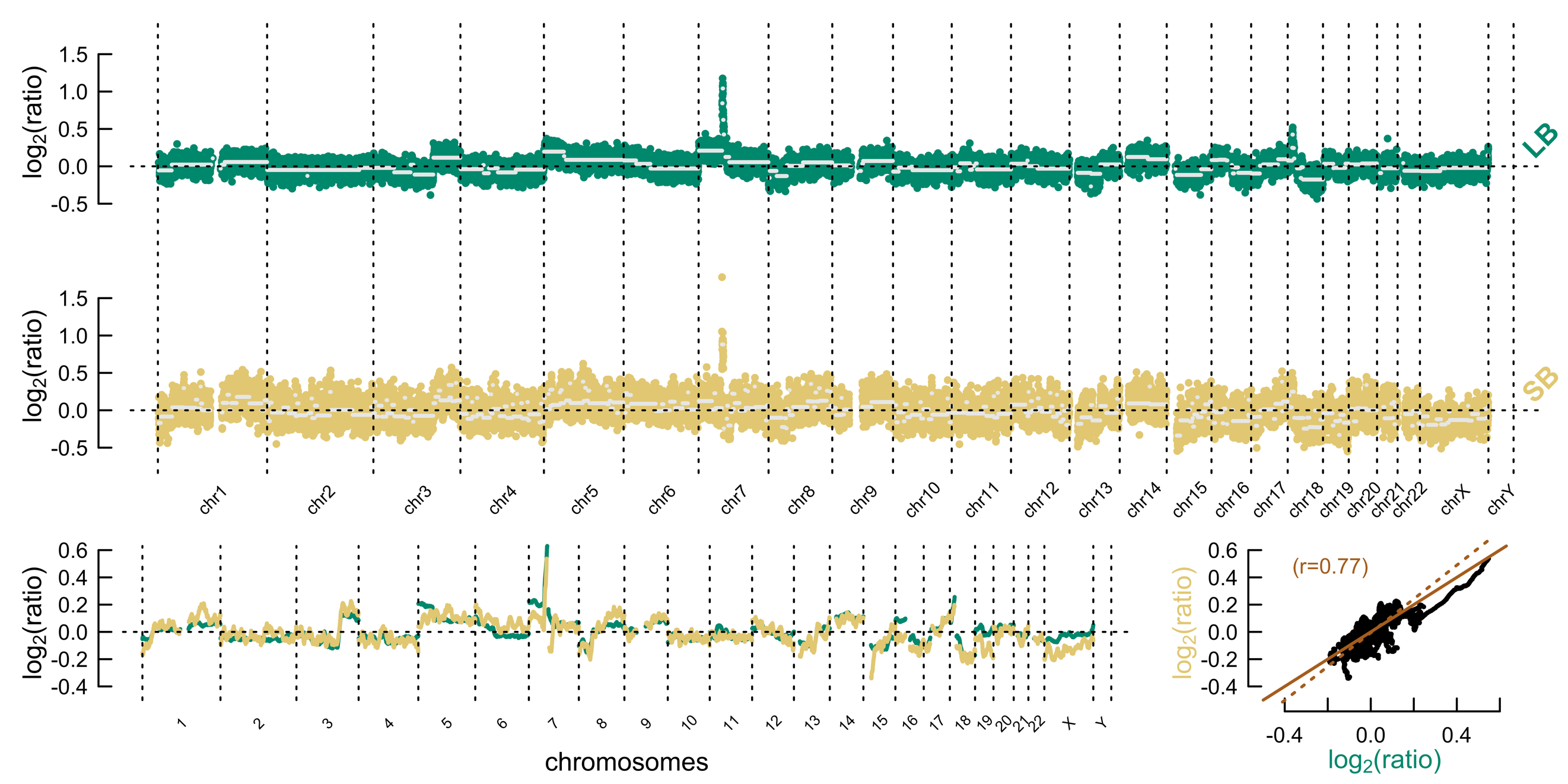


# Copy number profile(s) of patient 33


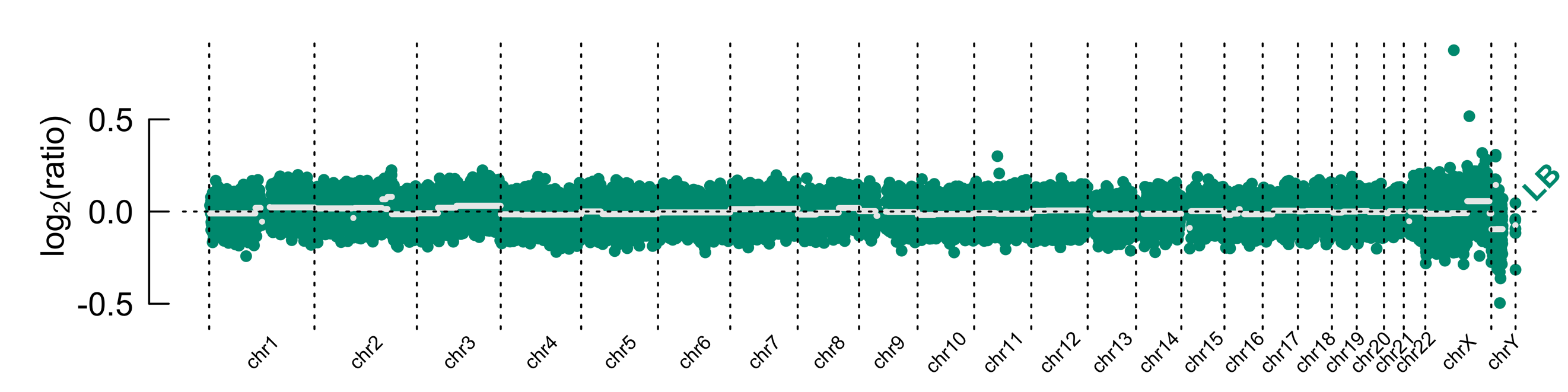


# Copy number profile(s) of patient 34


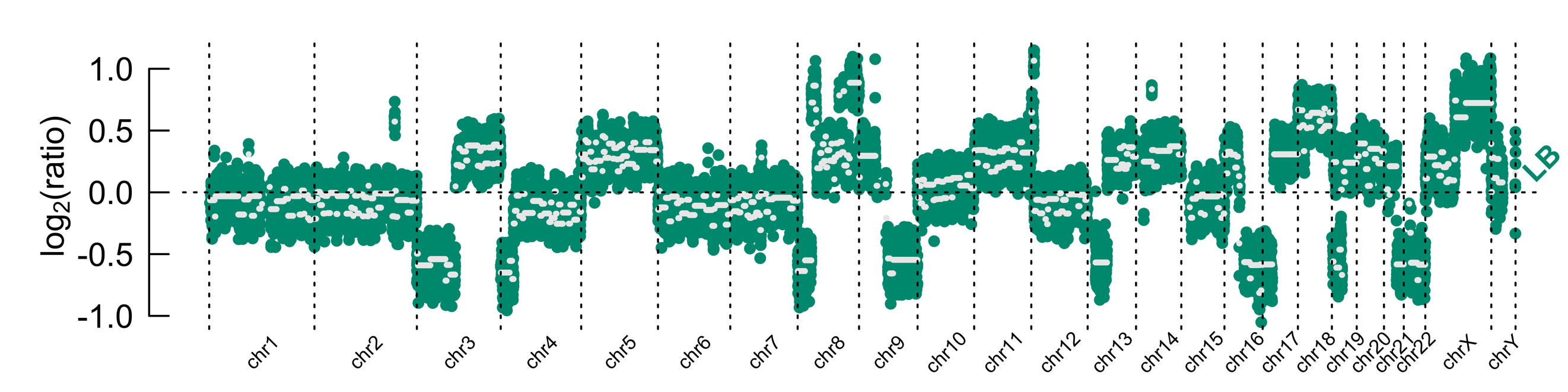


# Copy number profile(s) of patient 35


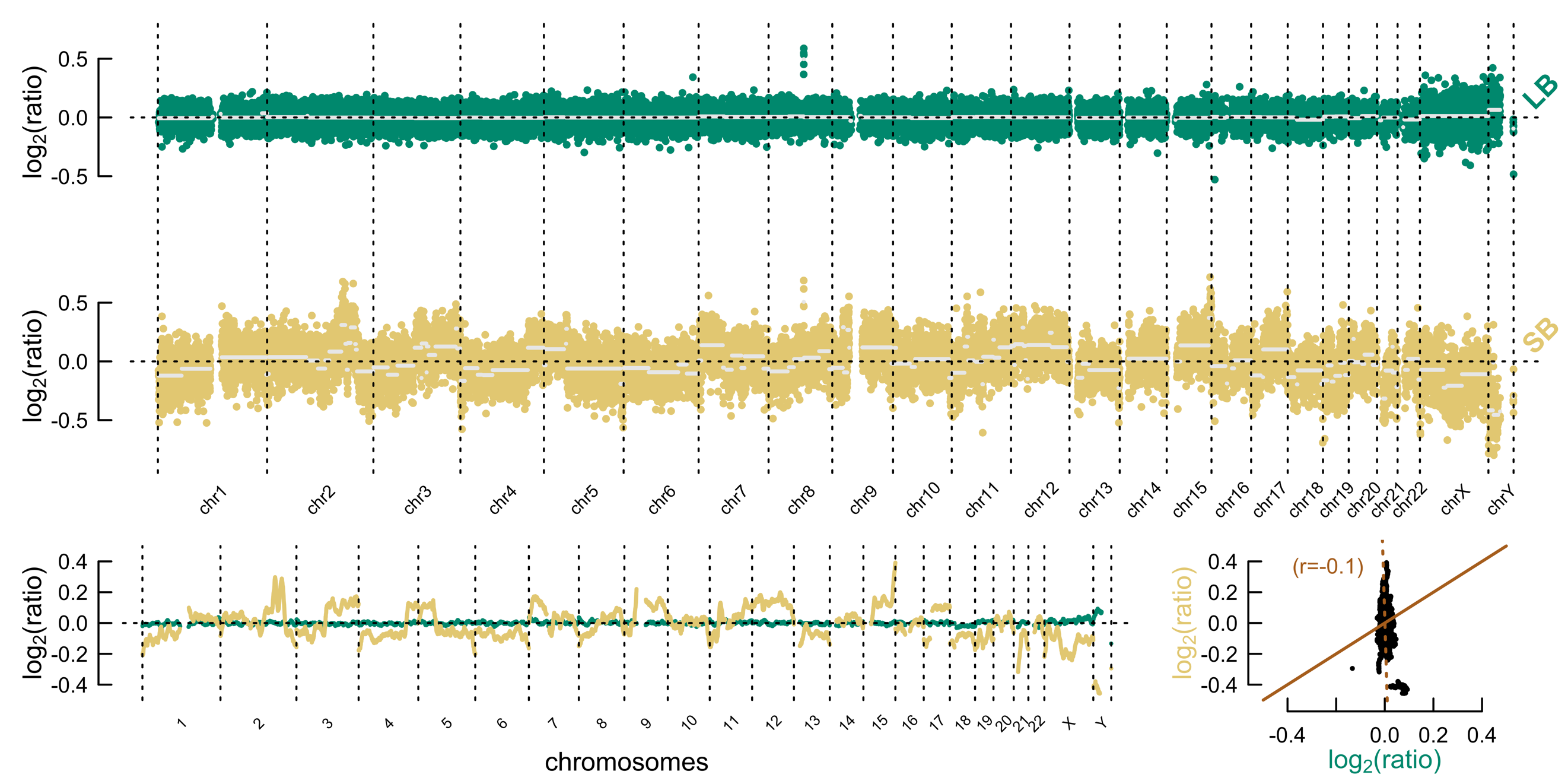


# Copy number profile(s) of patient 36


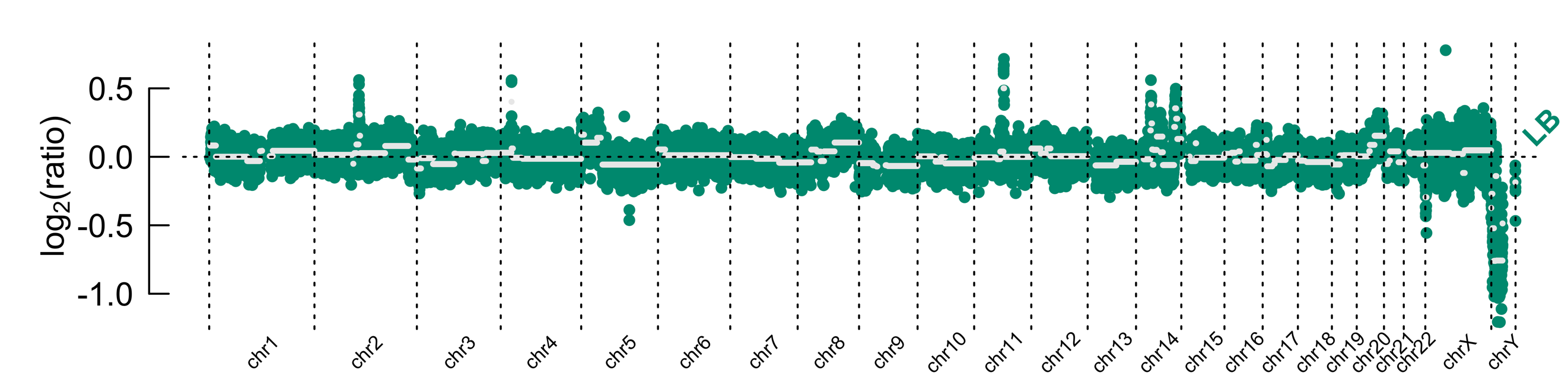


# Copy number profile(s) of patient 37


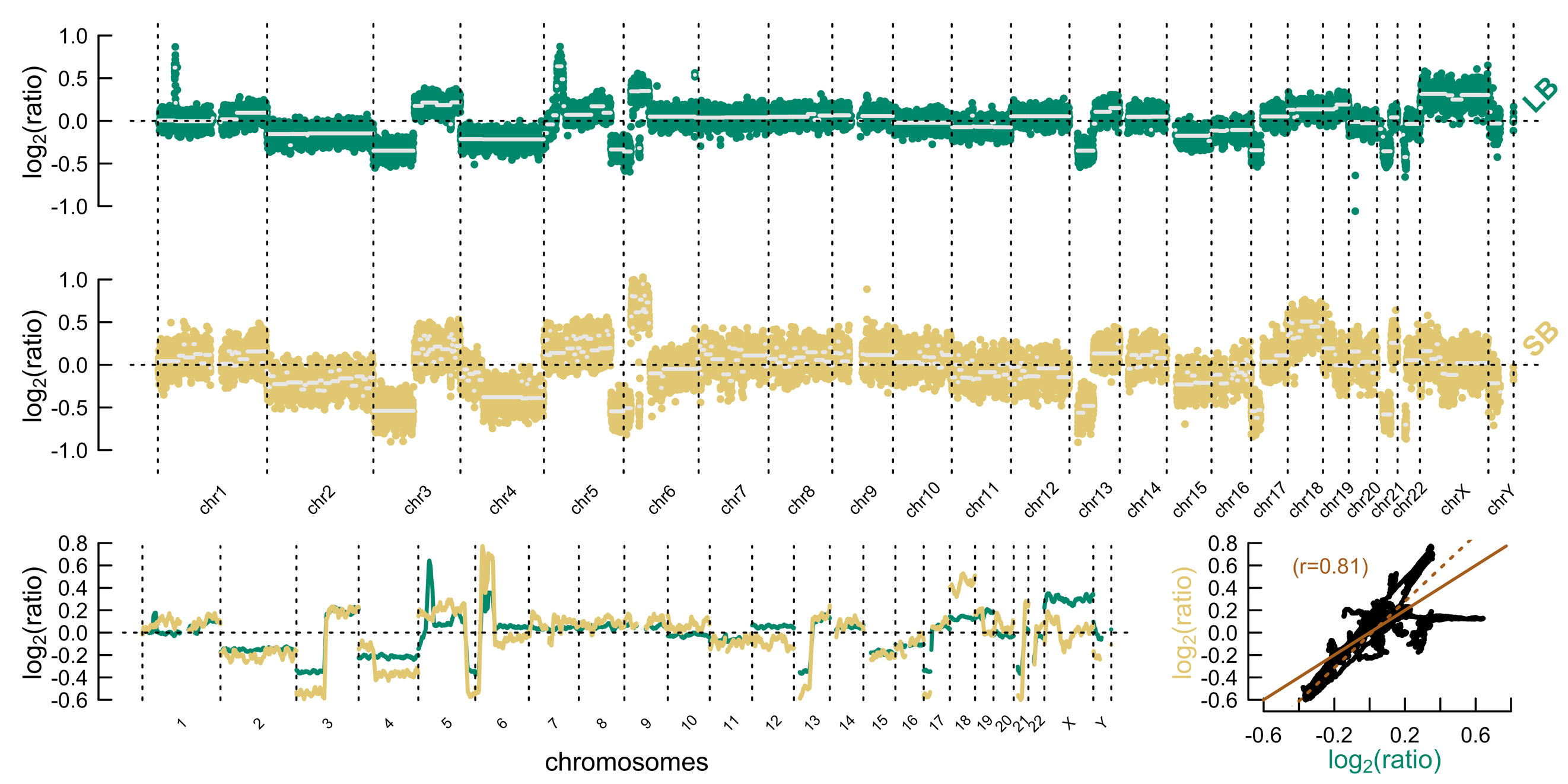


# Copy number profile(s) of patient 38


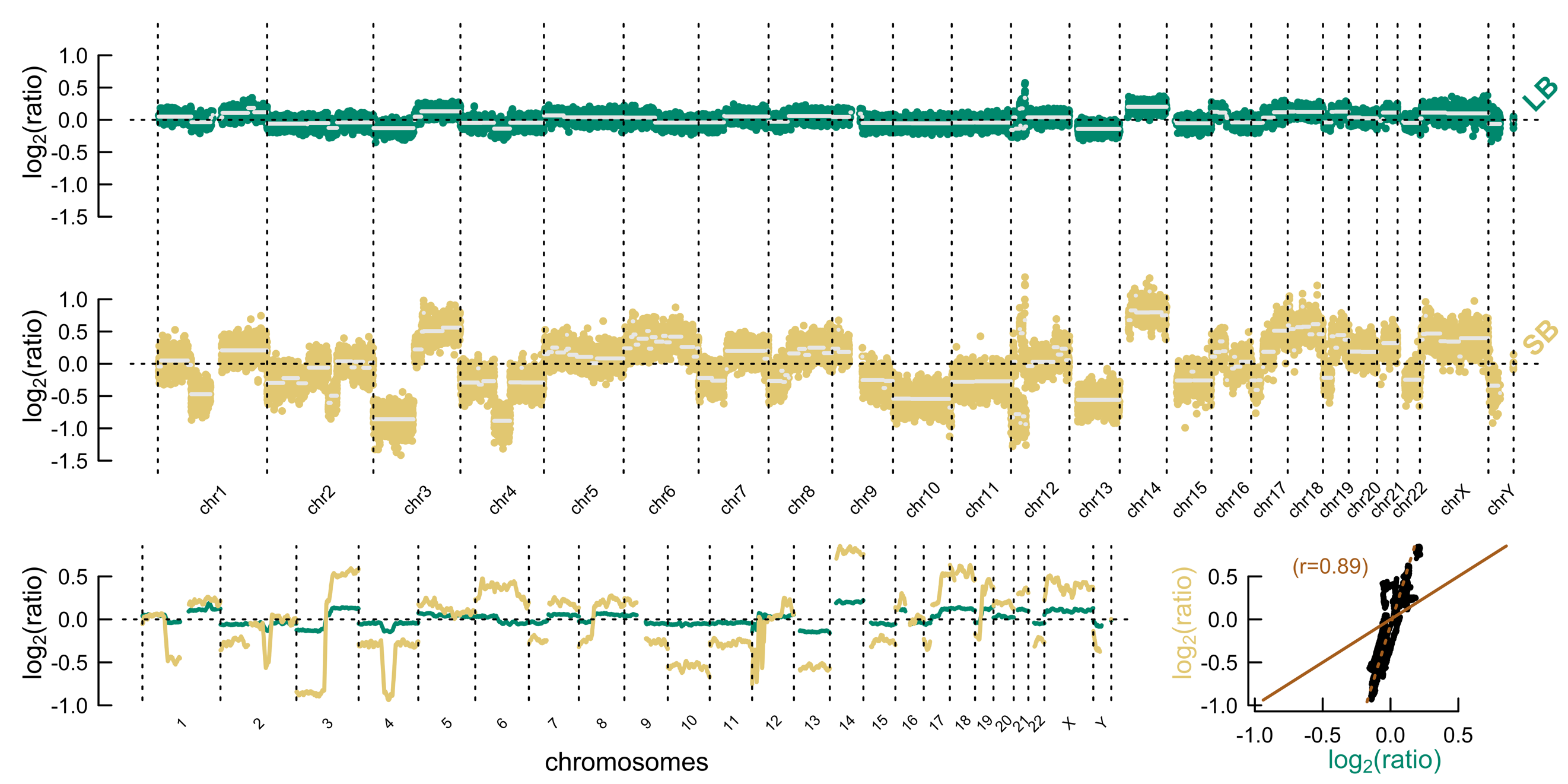


# Copy number profile(s) of patient 39


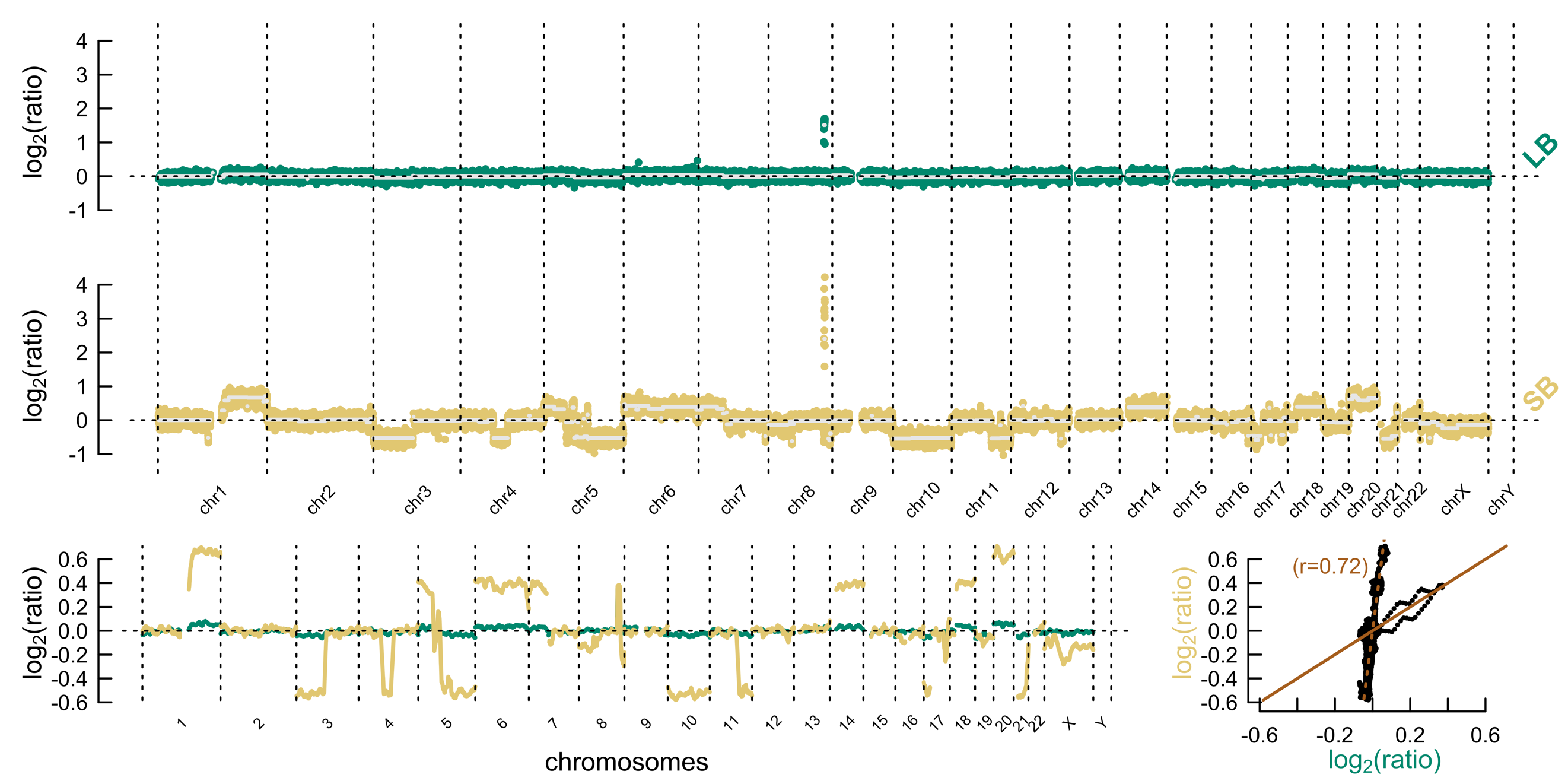


# Copy number profile(s) of patient 40


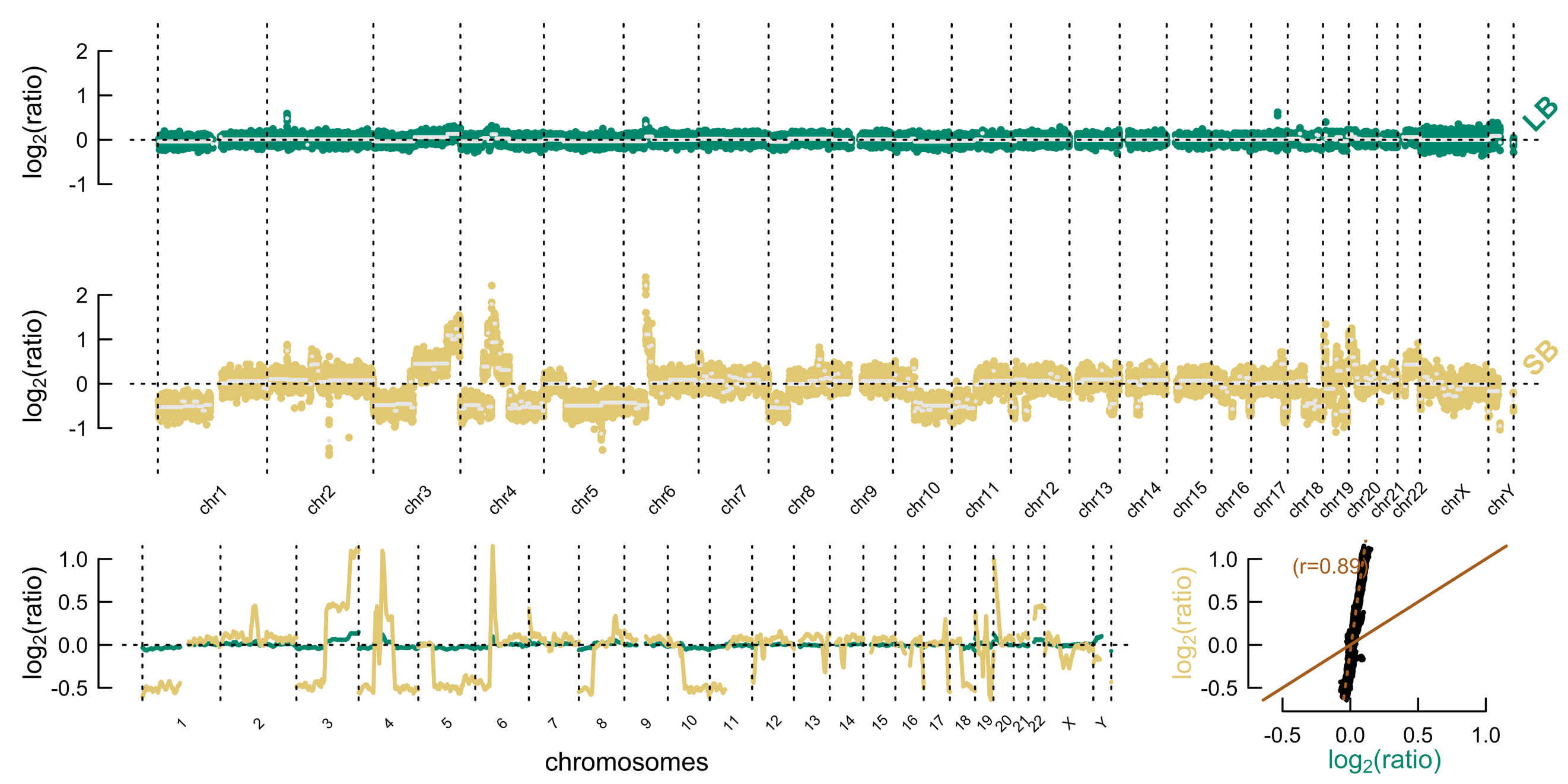


# Copy number profile(s) of patient 41


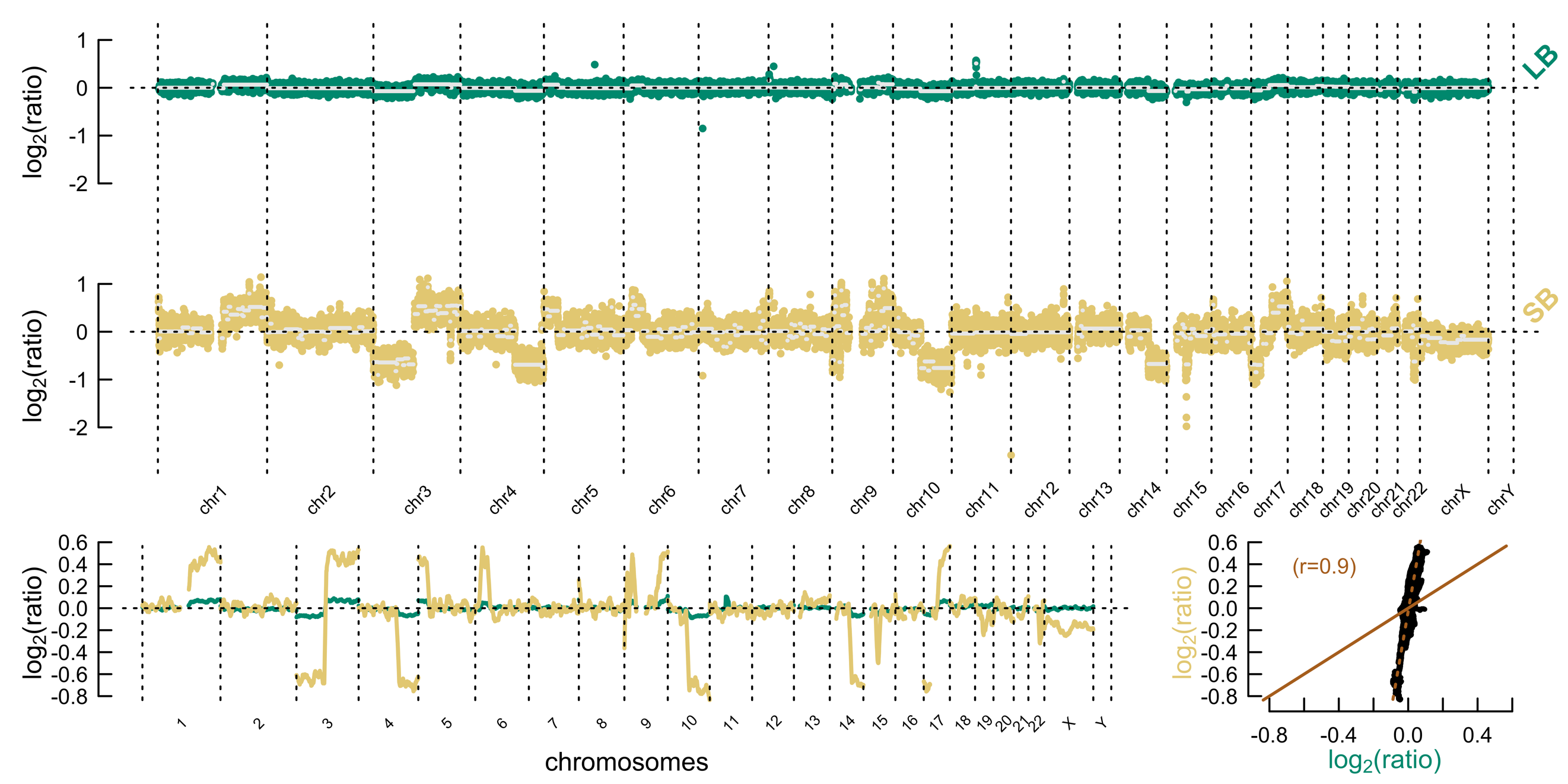


# Copy number profile(s) of patient 42


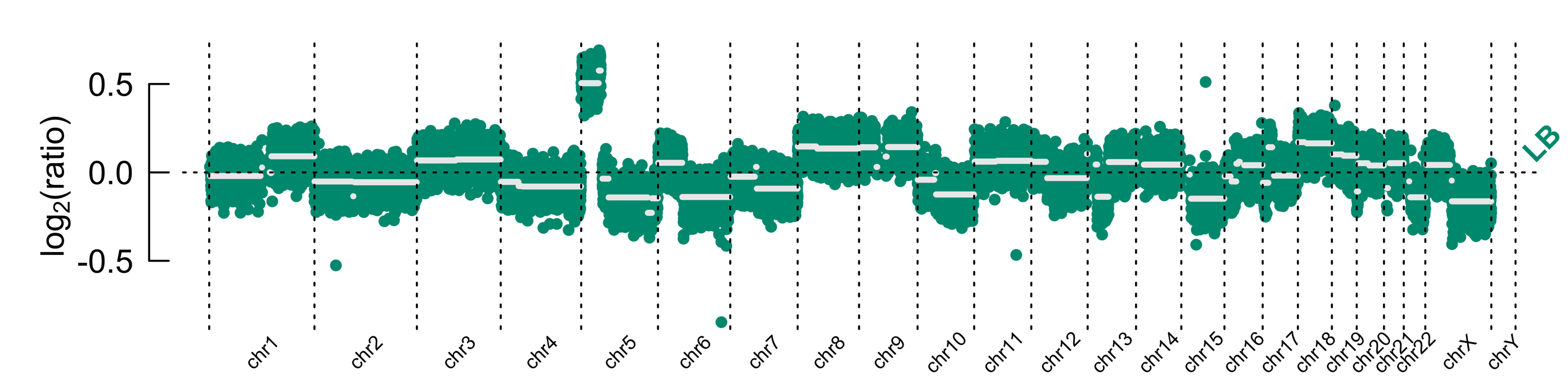


# Copy number profile(s) of patient 43


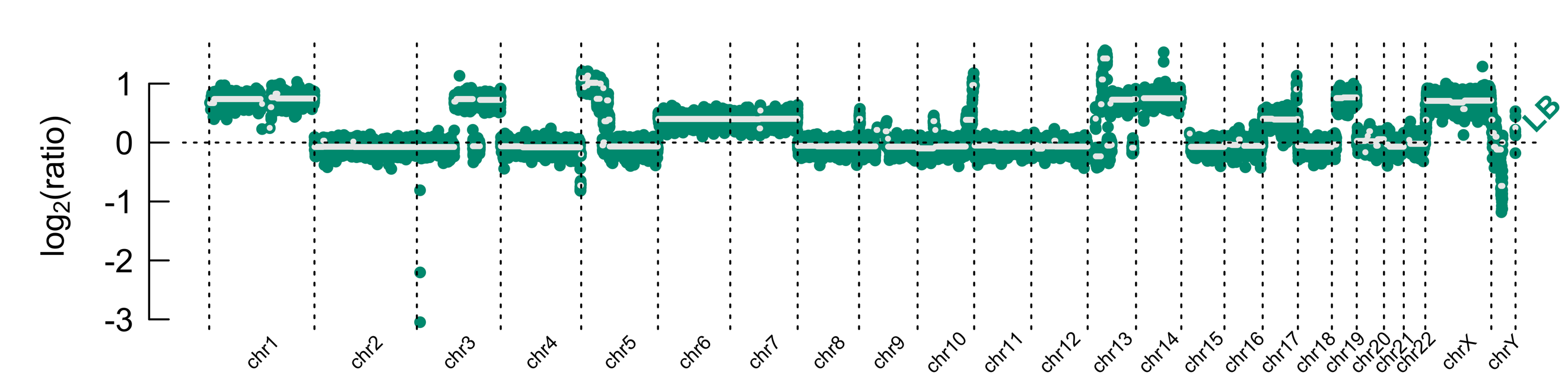


# Copy number profile(s) of patient 44


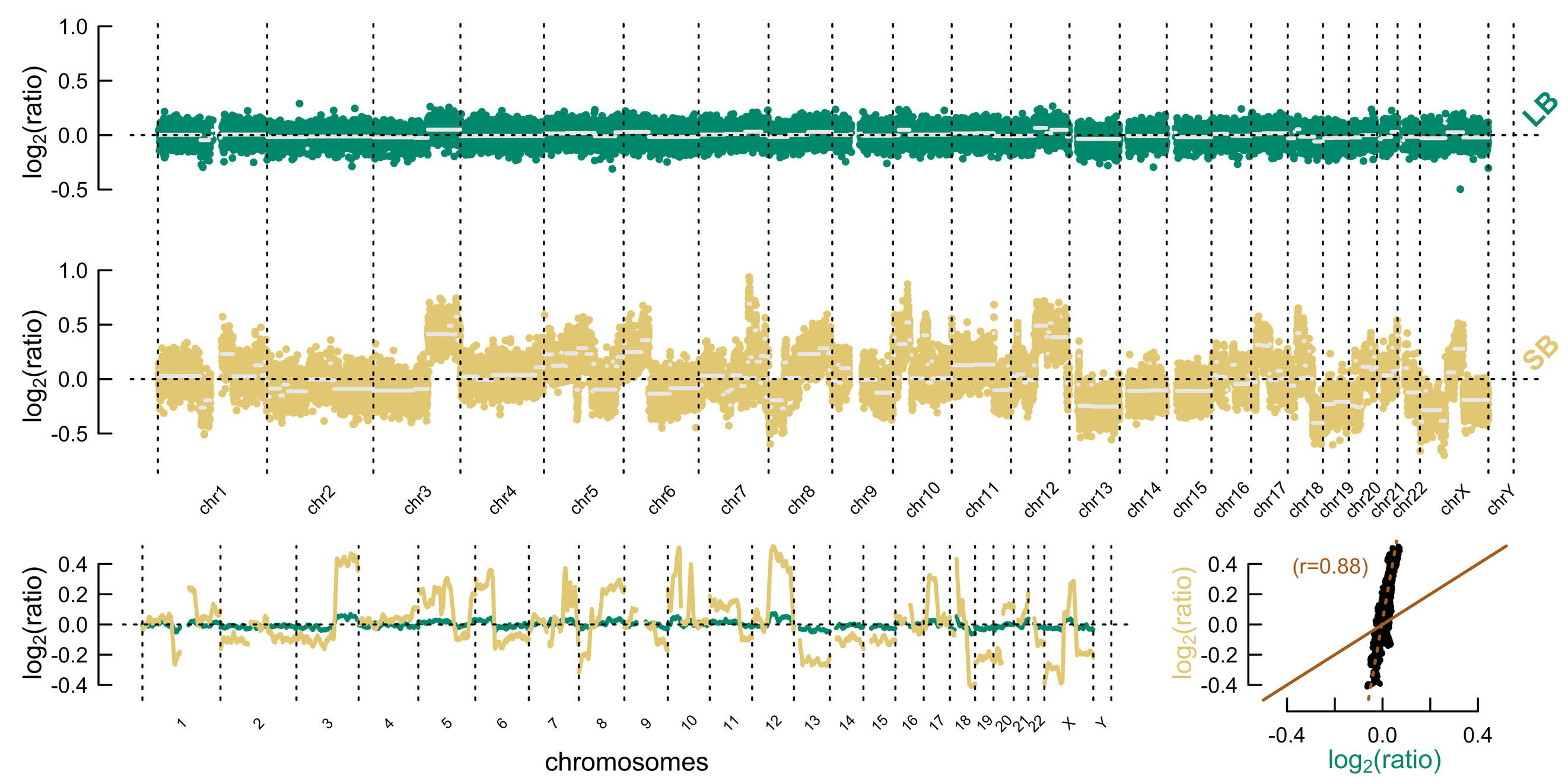


# Copy number profile(s) of patient 45


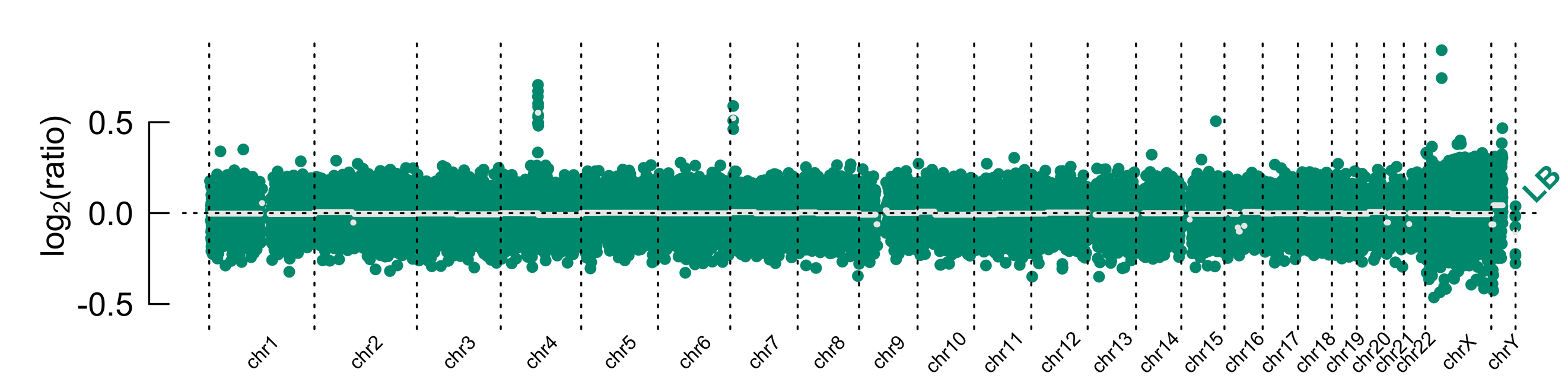


# Copy number profile(s) of patient 46


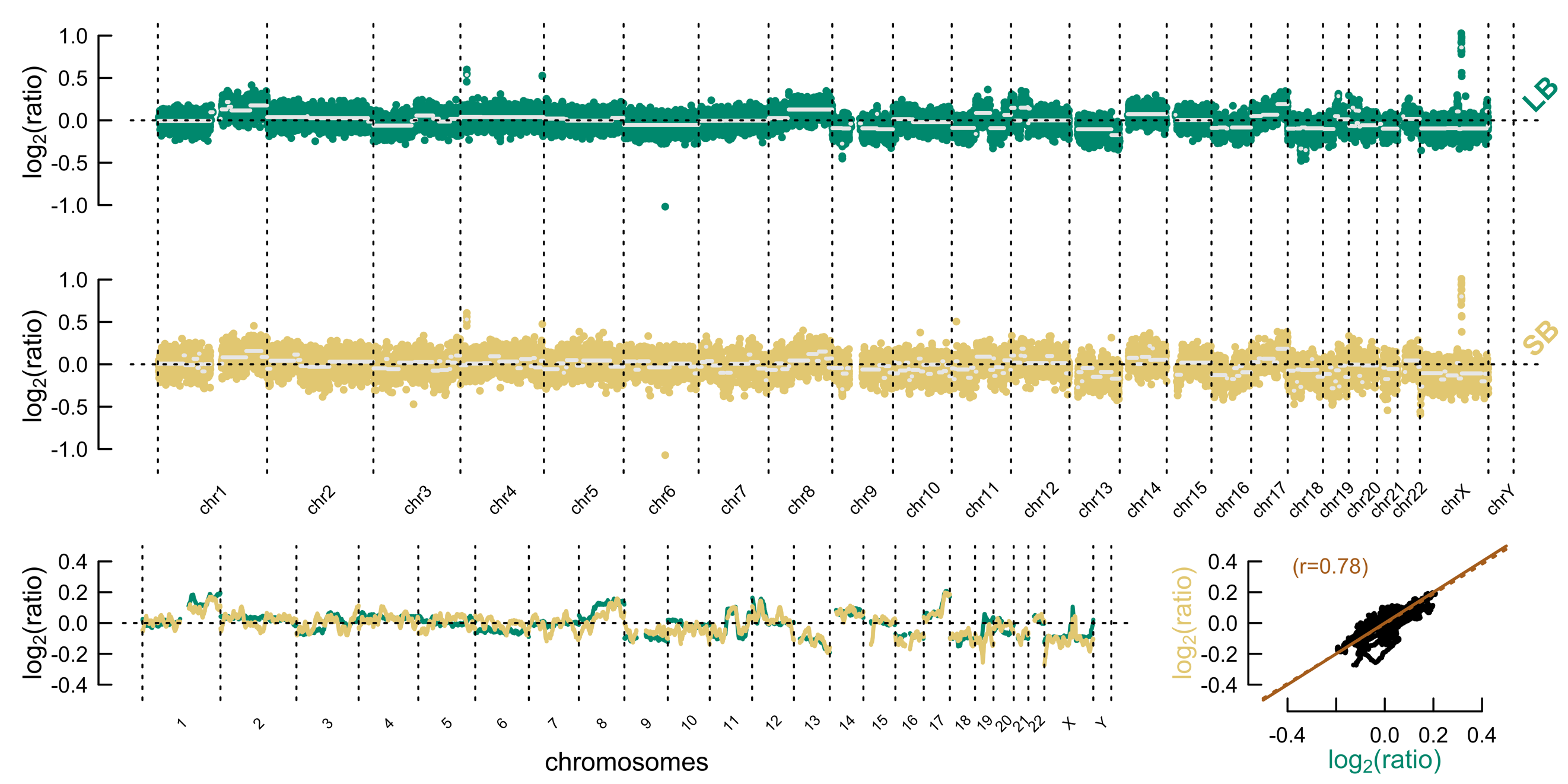


# Copy number profile(s) of patient 47


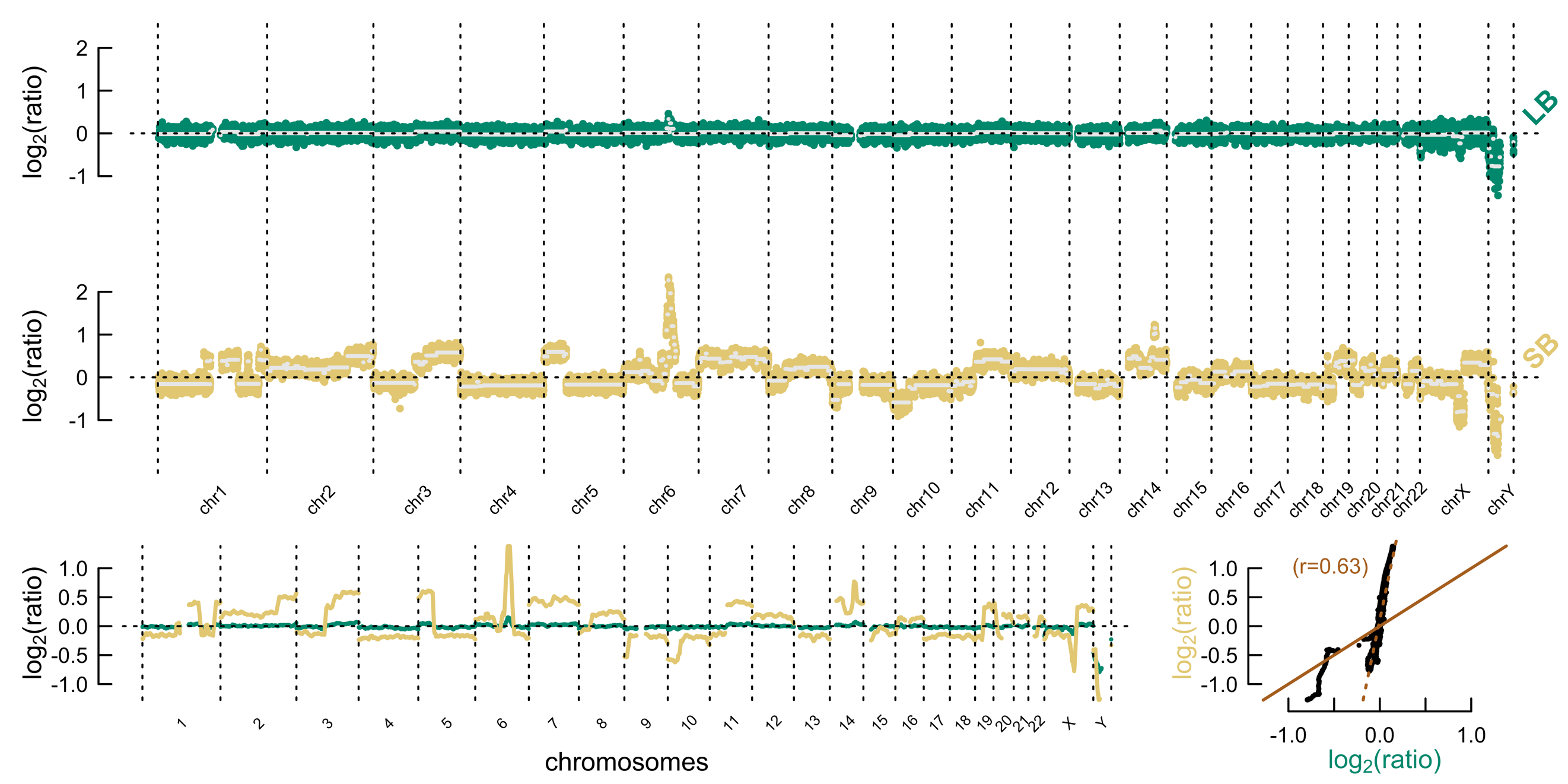


# Copy number profile(s) of patient 48


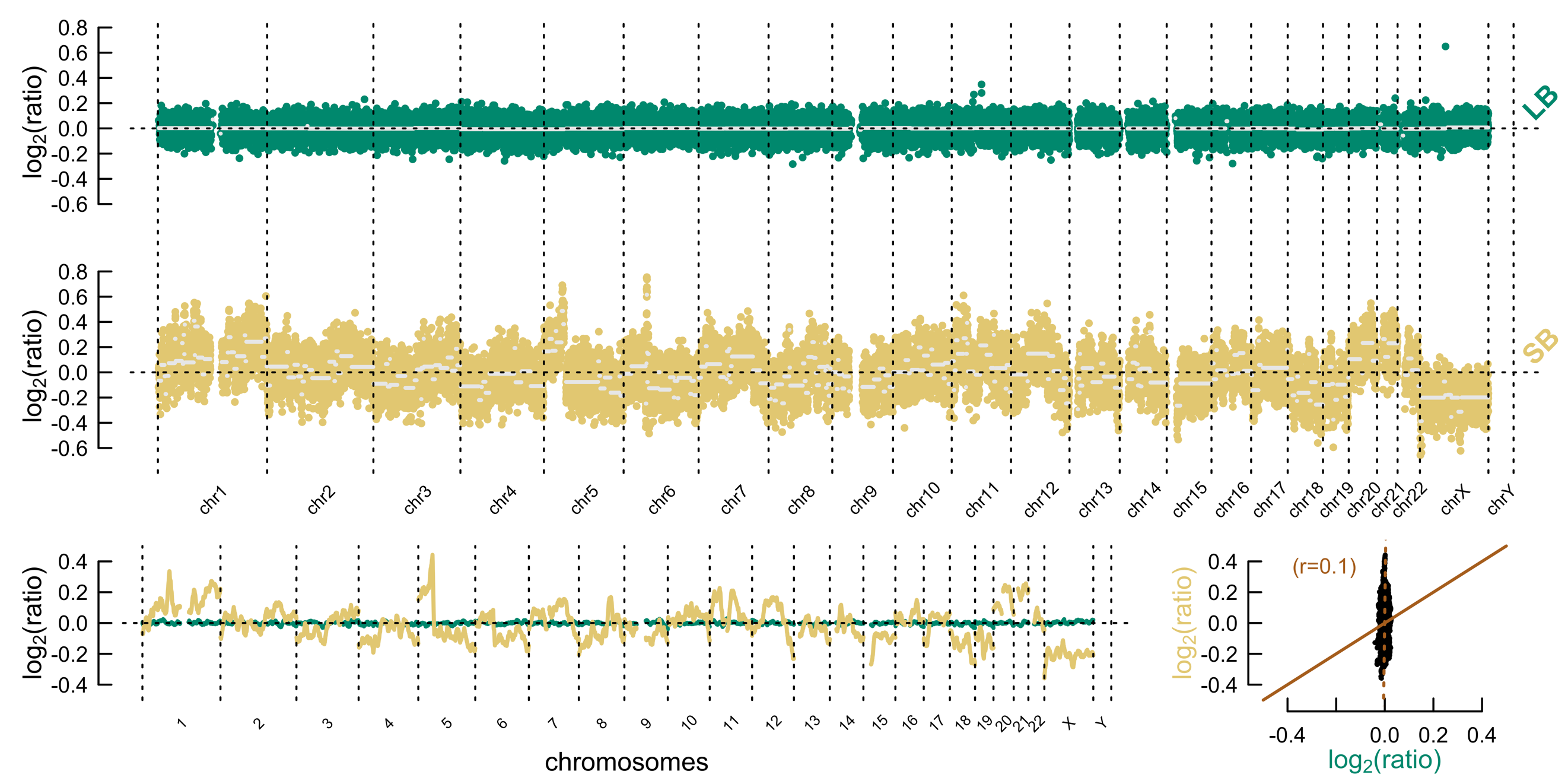


# Copy number profile(s) of patient 49


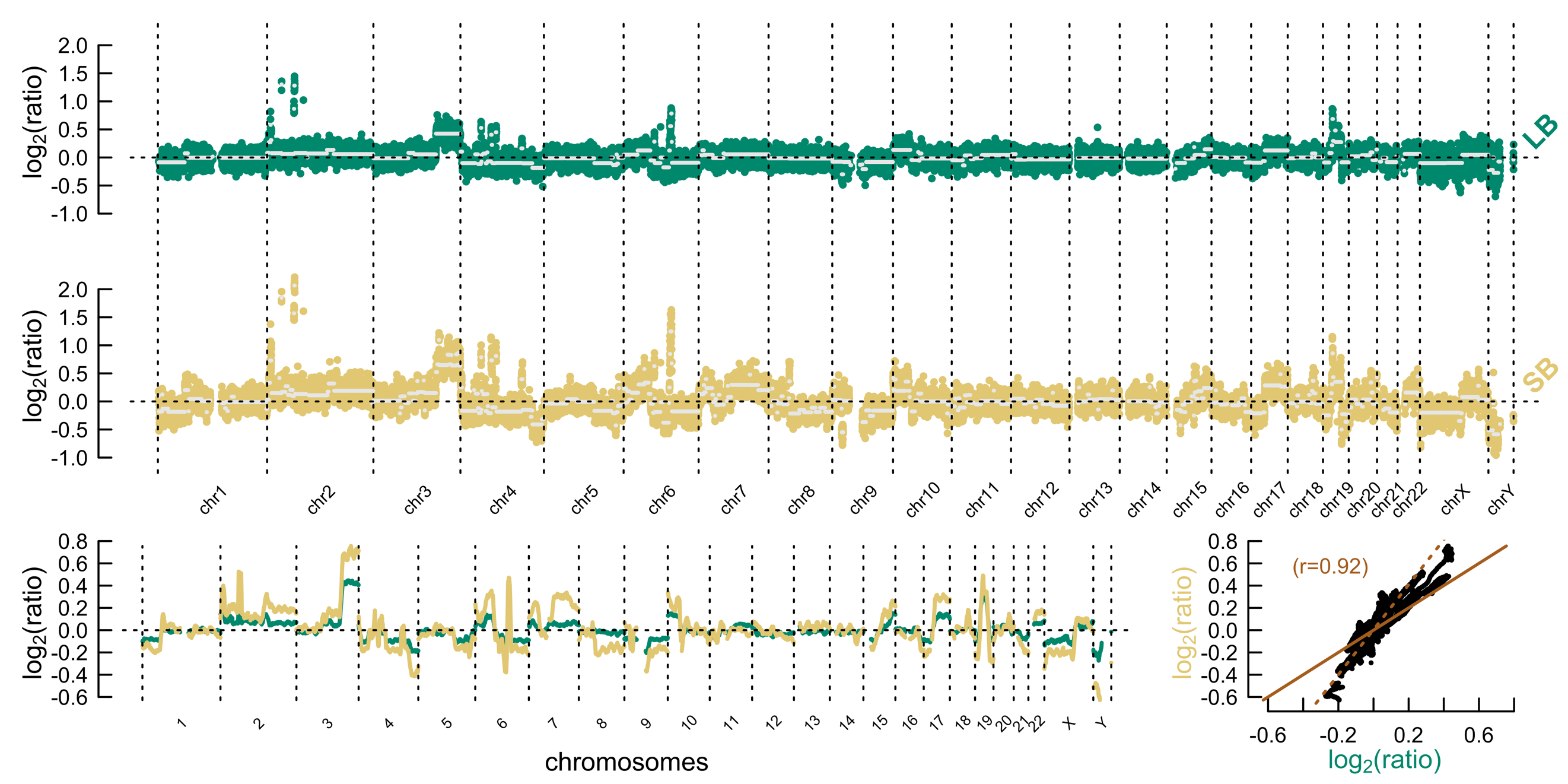


# Copy number profile(s) of patient 50


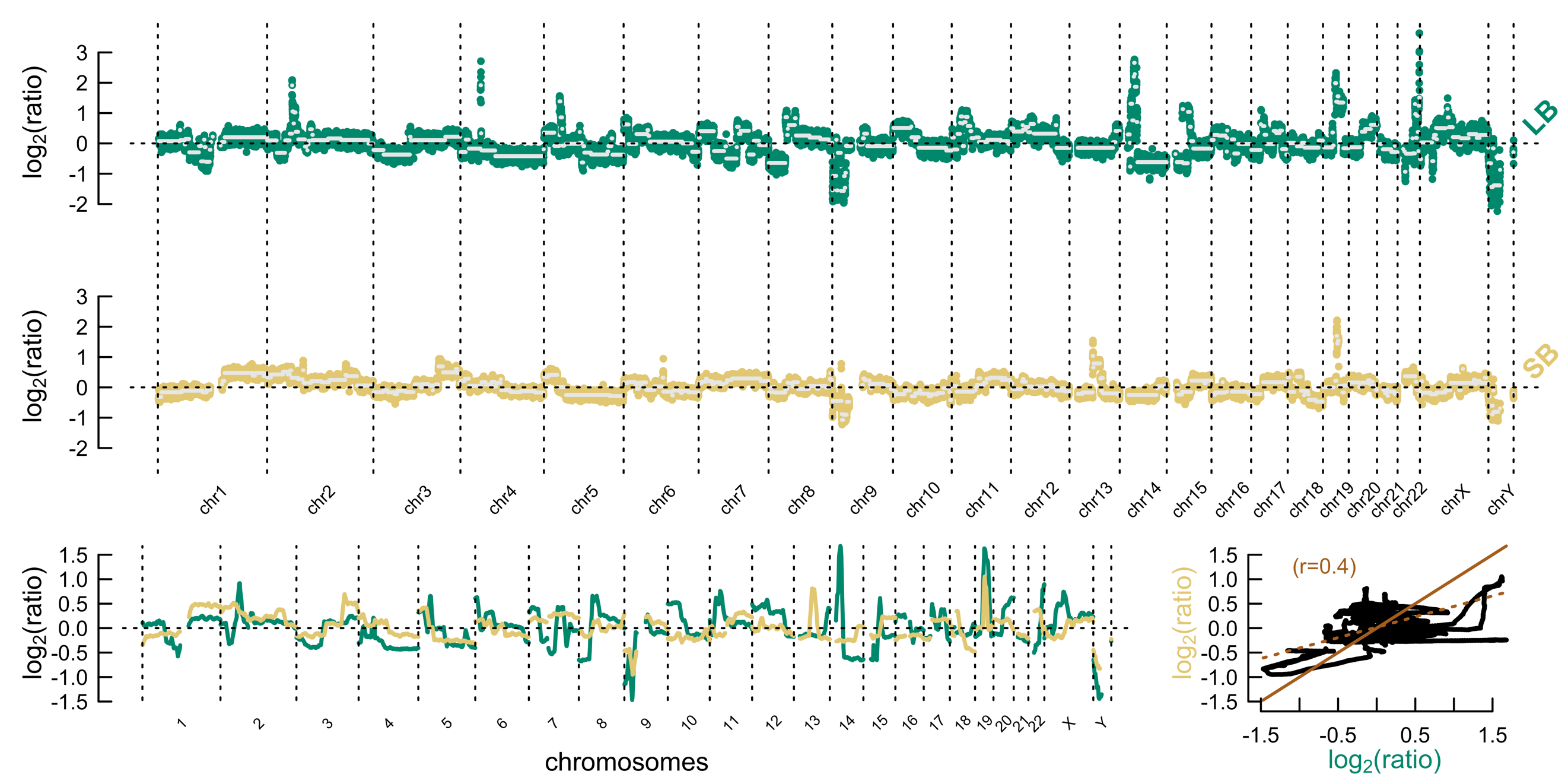


# Copy number profile(s) of patient 51


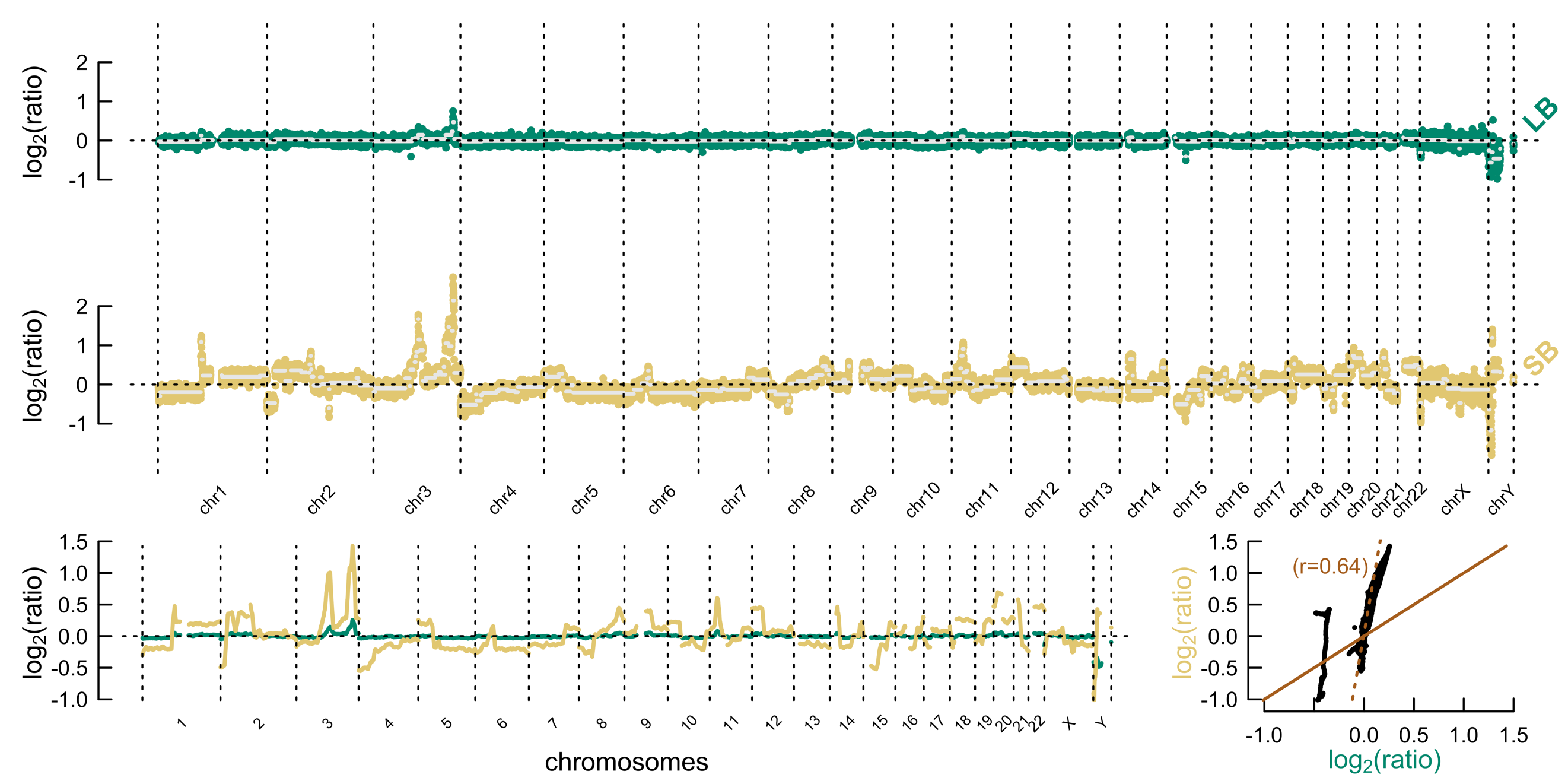

Supplement: Supplementary file 4 — Additional file 4. Supplementary single-end copy number profiles, containing all in-house lung cancer copy number profiles. [file 13073_2020_735_MOESM4_ESM.docx]
