# Supplementary material for: Shallow whole-genome sequencing of plasma cell-free DNA accurately differentiates small from non-small cell lung carcinoma
Source: Genome Med. 2020 Apr 21;12:35. doi: 10.1186/s13073-020-00735-4 (PMC7175544; doi:10.1186/s13073-020-00735-4)

[Copy number profiles of patient 1 1](#_Toc34144606)

[Copy number profiles of patient 2 1](#_Toc34144607)

[Copy number profiles of patient 3 2](#_Toc34144608)

[Copy number profiles of patient 4 2](#_Toc34144609)

[Copy number profiles of patient 5 3](#_Toc34144610)

[Copy number profiles of patient 8 3](#_Toc34144611)

[Copy number profiles of patient 10 4](#_Toc34144612)

[Copy number profiles of patient 11 4](#_Toc34144613)

[Copy number profiles of patient 13 5](#_Toc34144614)

[Copy number profiles of patient 14 5](#_Toc34144615)

[Copy number profiles of patient 15 6](#_Toc34144616)

[Copy number profiles of patient 17 6](#_Toc34144617)

[Copy number profiles of patient 18 7](#_Toc34144618)

[Copy number profiles of patient 20 7](#_Toc34144619)

[Copy number profiles of patient 27 8](#_Toc34144620)

[Copy number profiles of patient 30 8](#_Toc34144621)

[Copy number profiles of patient 32 9](#_Toc34144622)

[Copy number profiles of patient 33 9](#_Toc34144623)

[Copy number profiles of patient 34 10](#_Toc34144624)

[Copy number profiles of patient 35 10](#_Toc34144625)

[Copy number profiles of patient 36 11](#_Toc34144626)

[Copy number profiles of patient 37 11](#_Toc34144627)

[Copy number profiles of patient 38 12](#_Toc34144628)

[Copy number profiles of patient 39 12](#_Toc34144629)

[Copy number profiles of patient 40 13](#_Toc34144630)

[Copy number profiles of patient 41 13](#_Toc34144631)

[Copy number profiles of patient 42 14](#_Toc34144632)

[Copy number profiles of patient 43 14](#_Toc34144633)

[Copy number profiles of patient 44 15](#_Toc34144634)

# Copy number profiles of patient 1


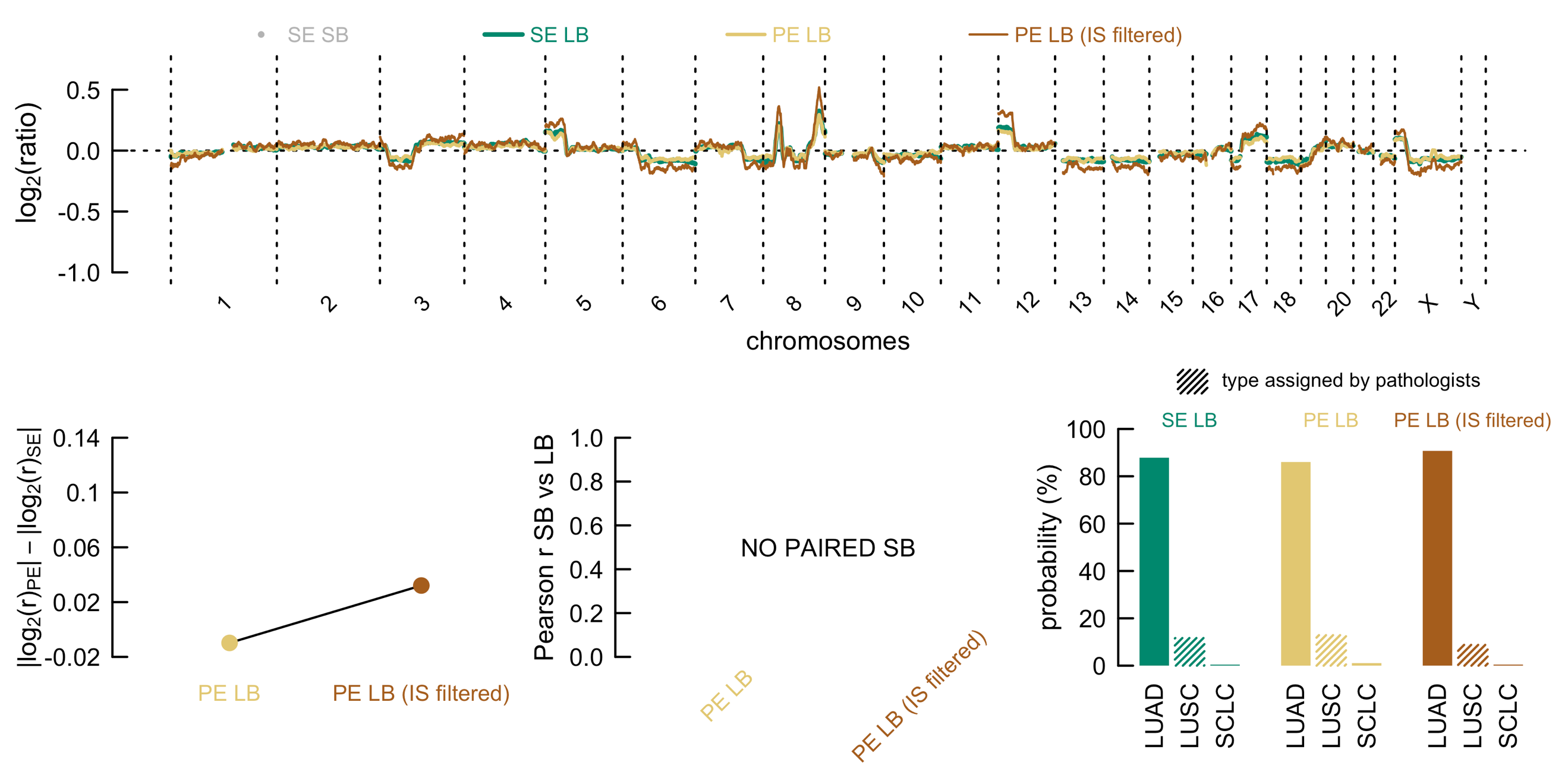


# Copy number profiles of patient 2


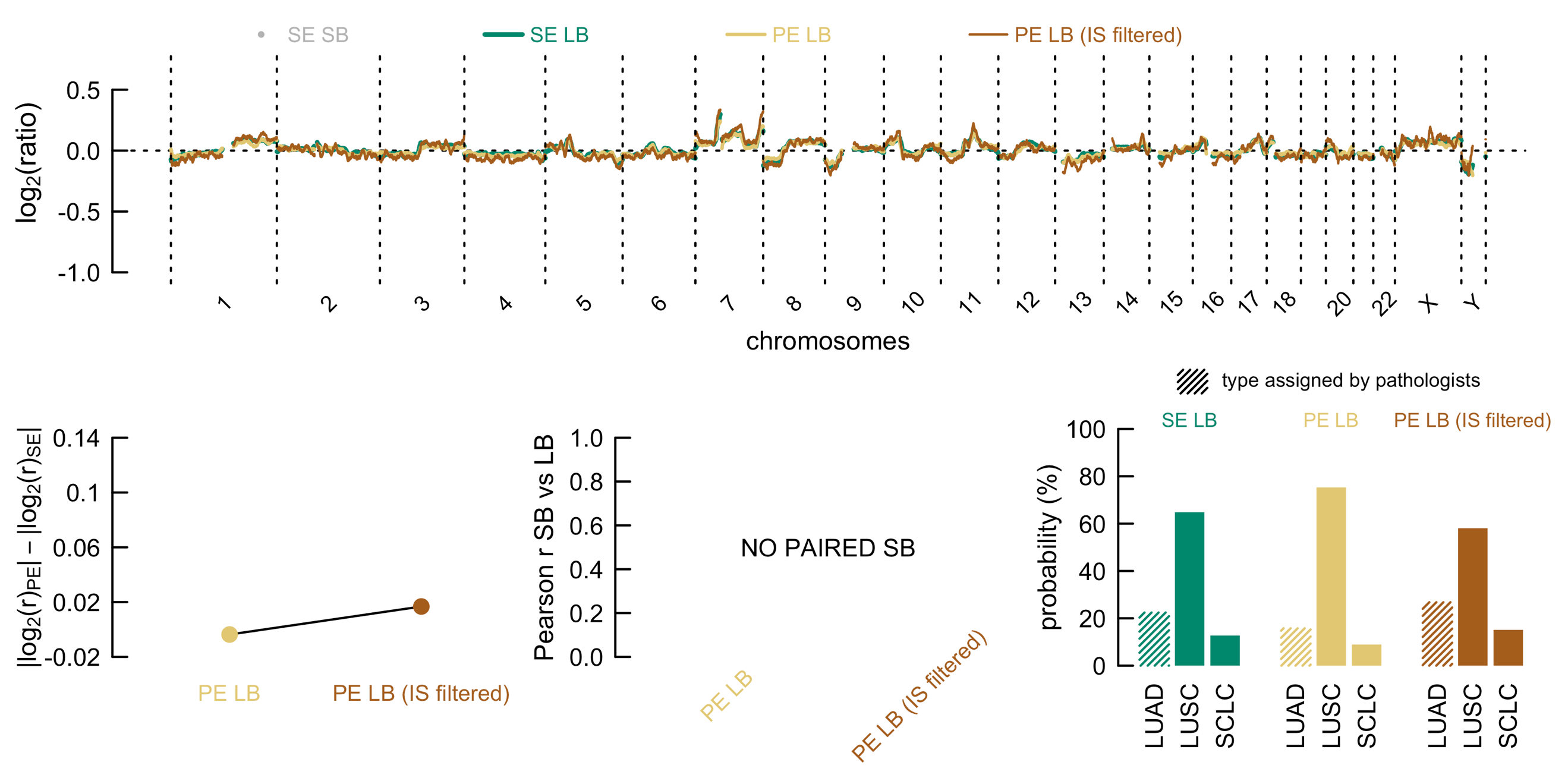


# Copy number profiles of patient 3


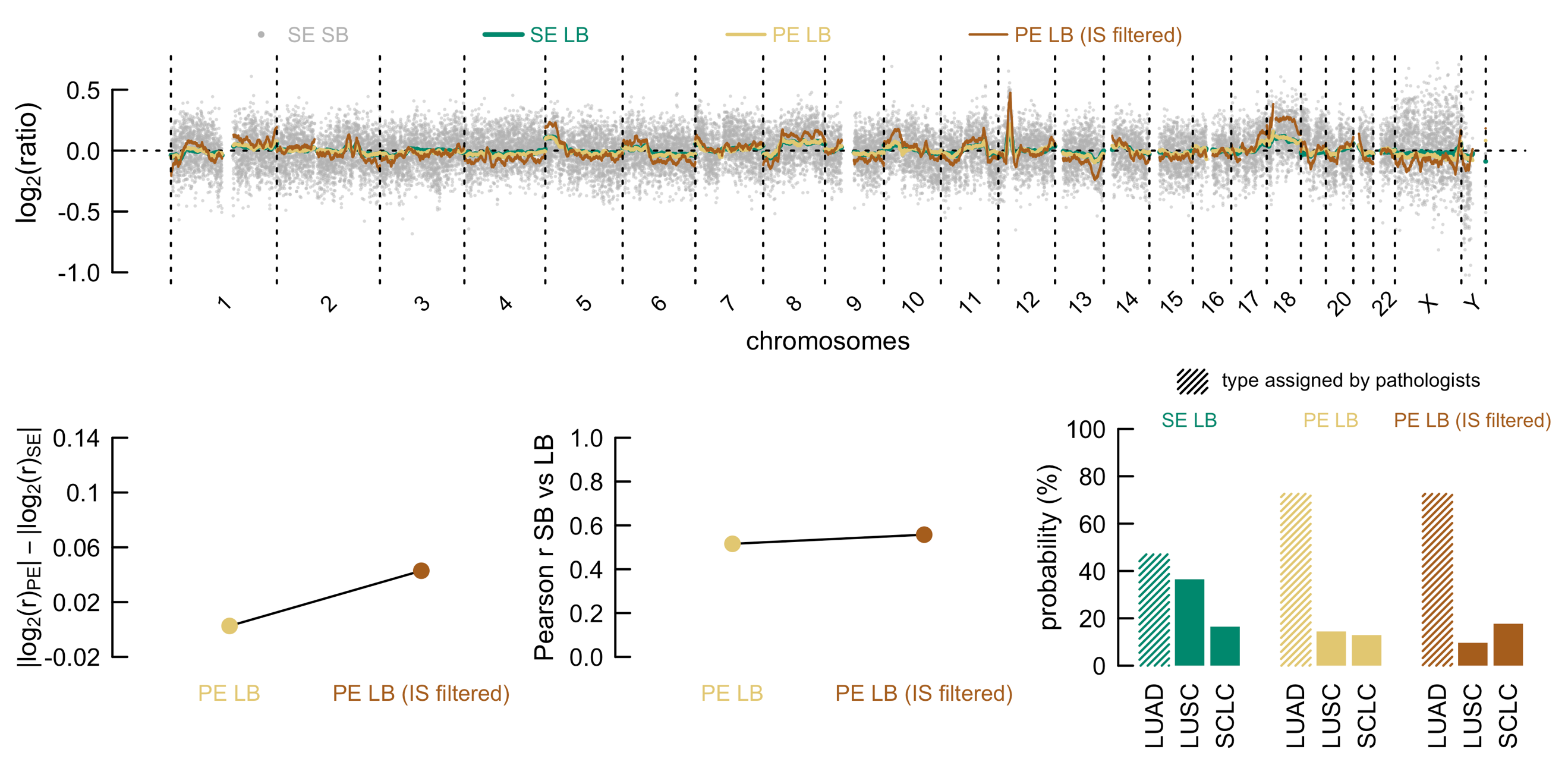


# Copy number profiles of patient 4


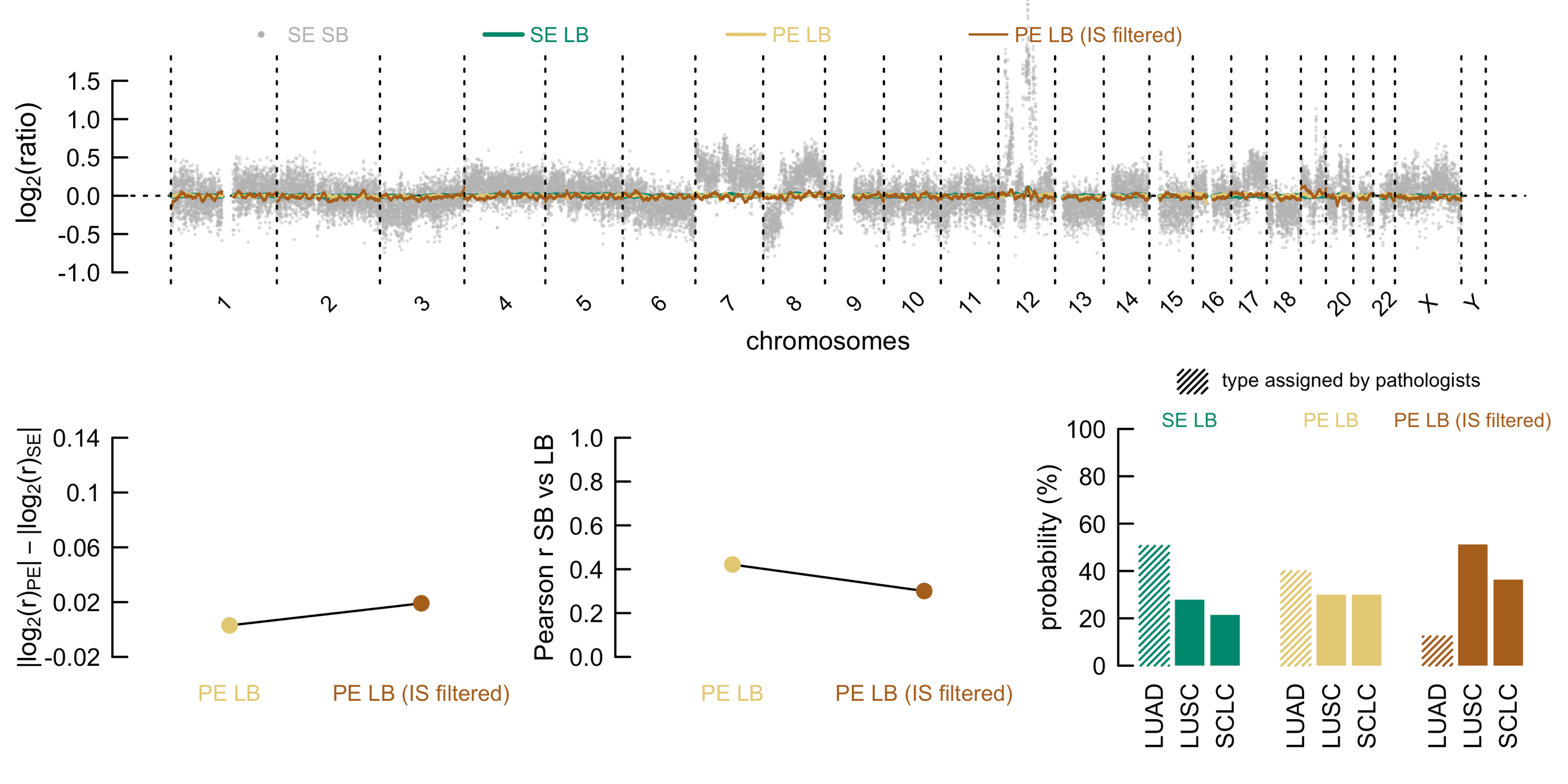


# Copy number profiles of patient 5


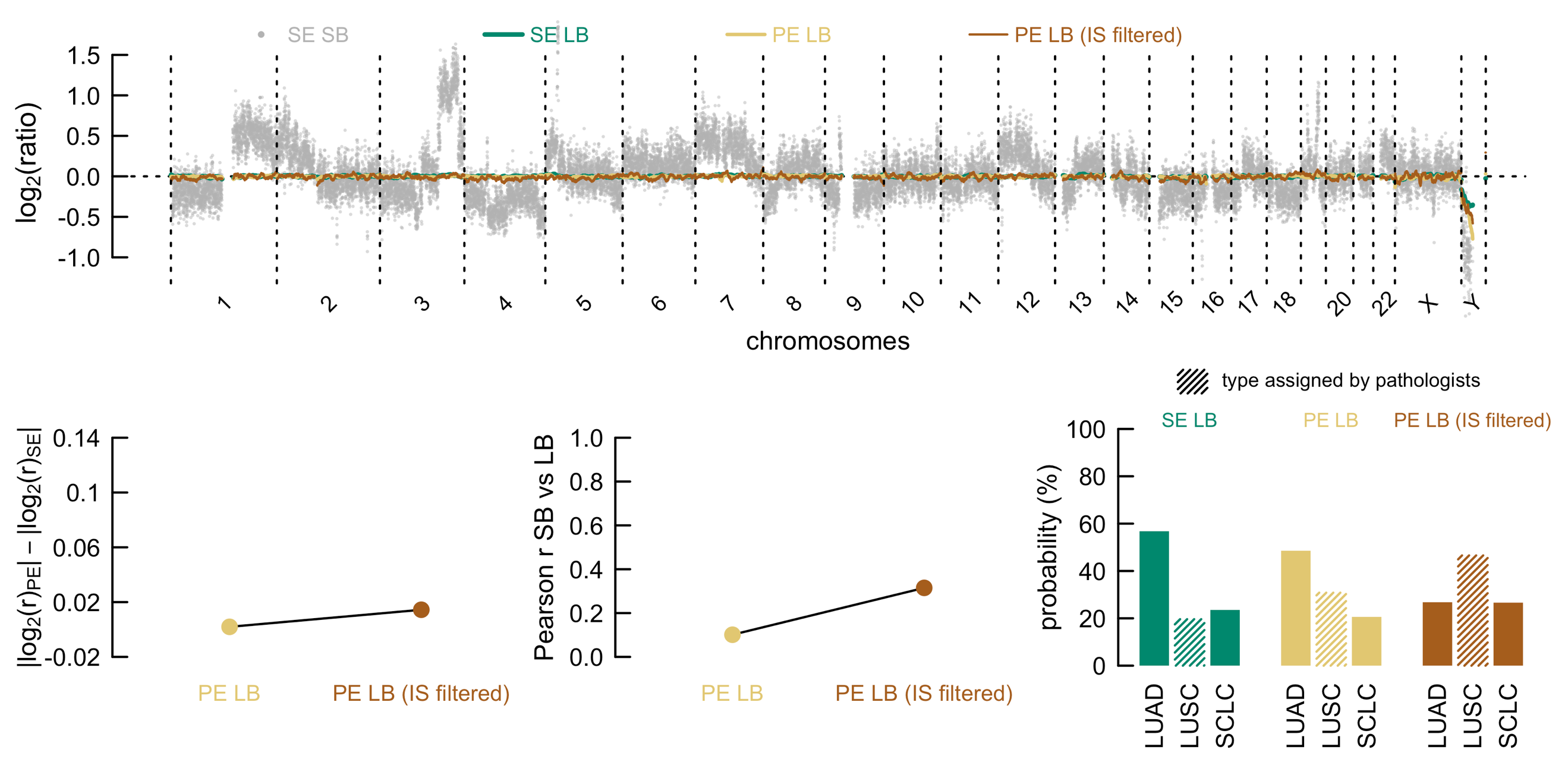


# Copy number profiles of patient 8


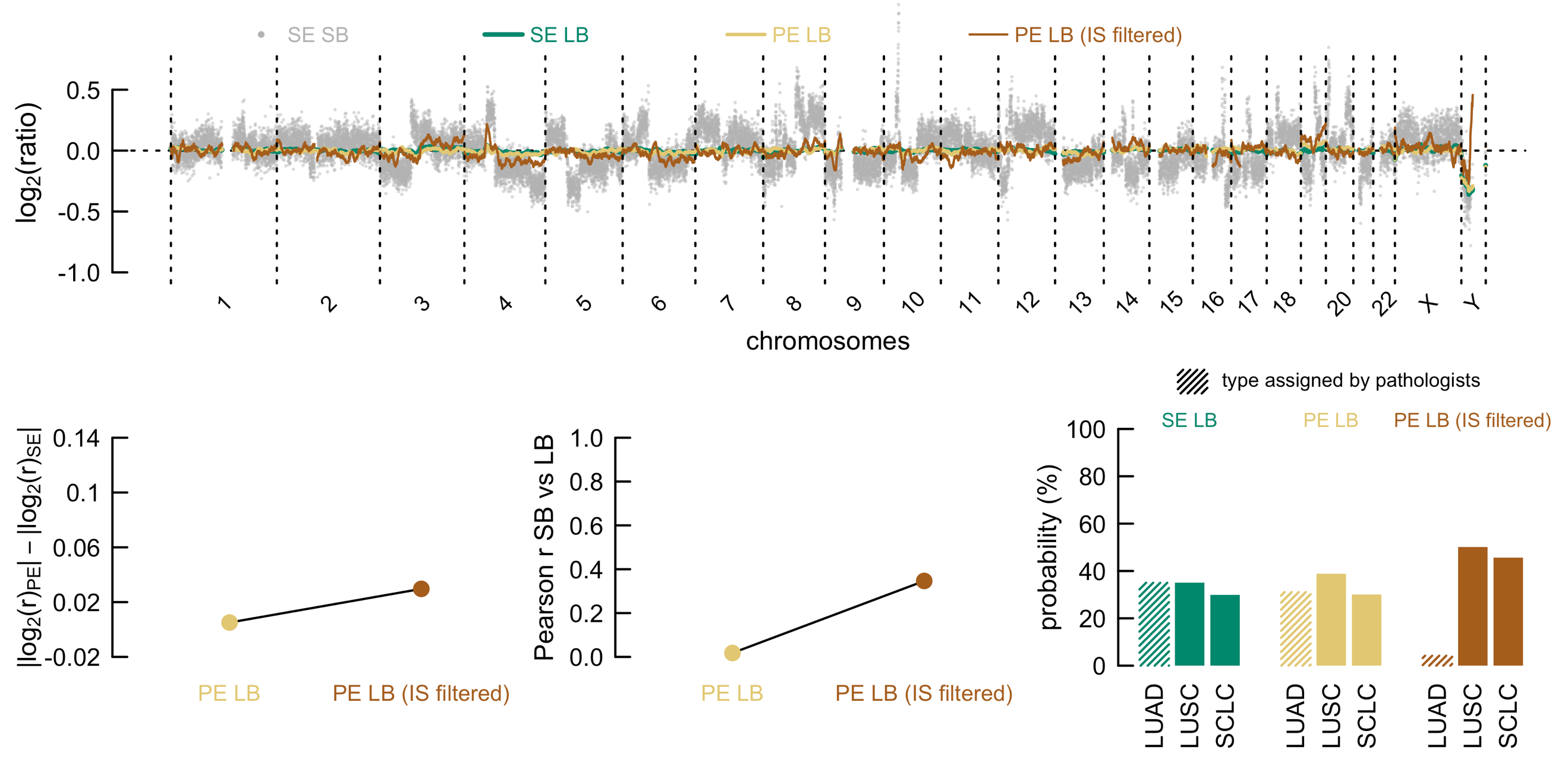


# Copy number profiles of patient 10


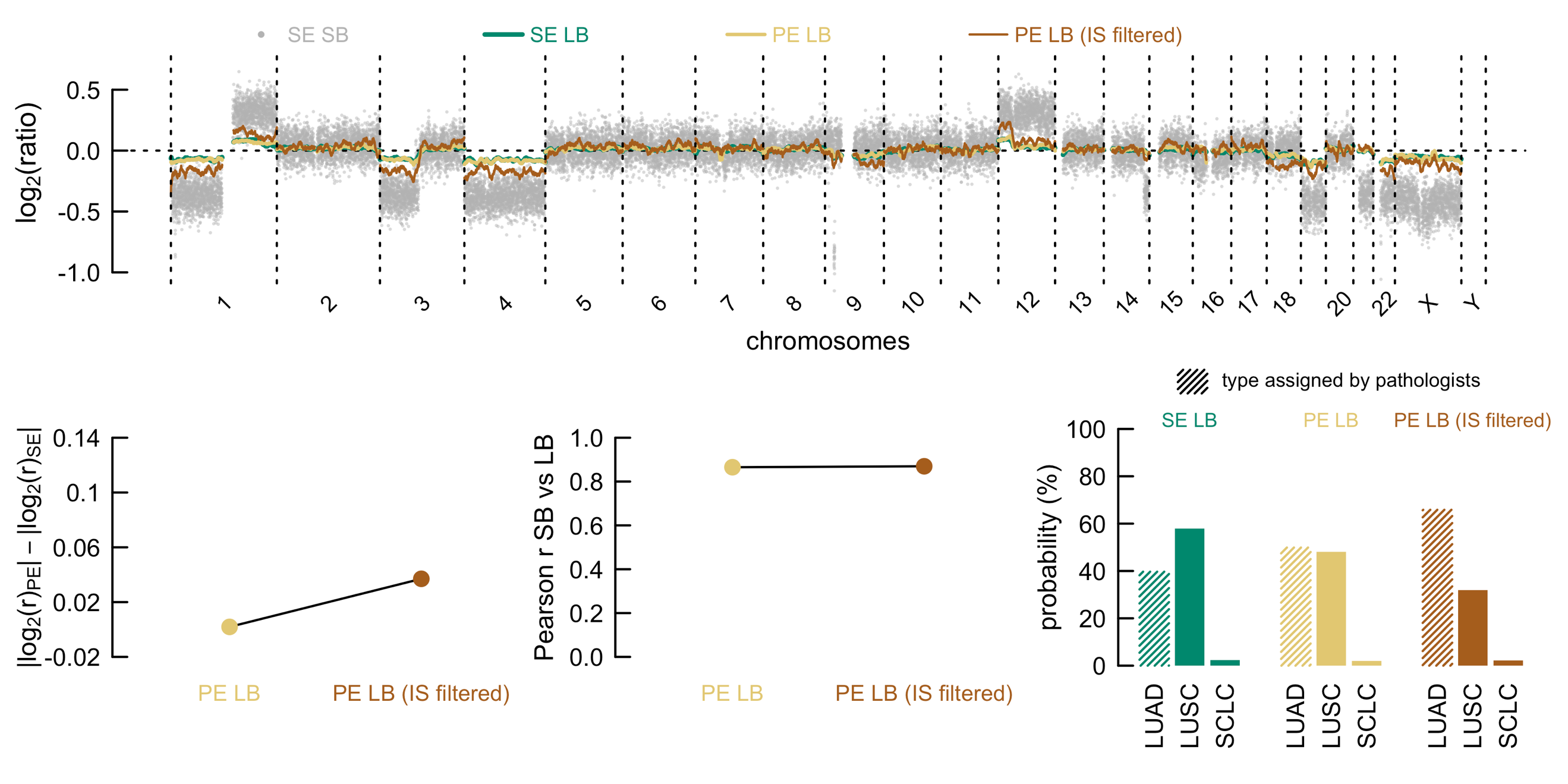


# Copy number profiles of patient 11


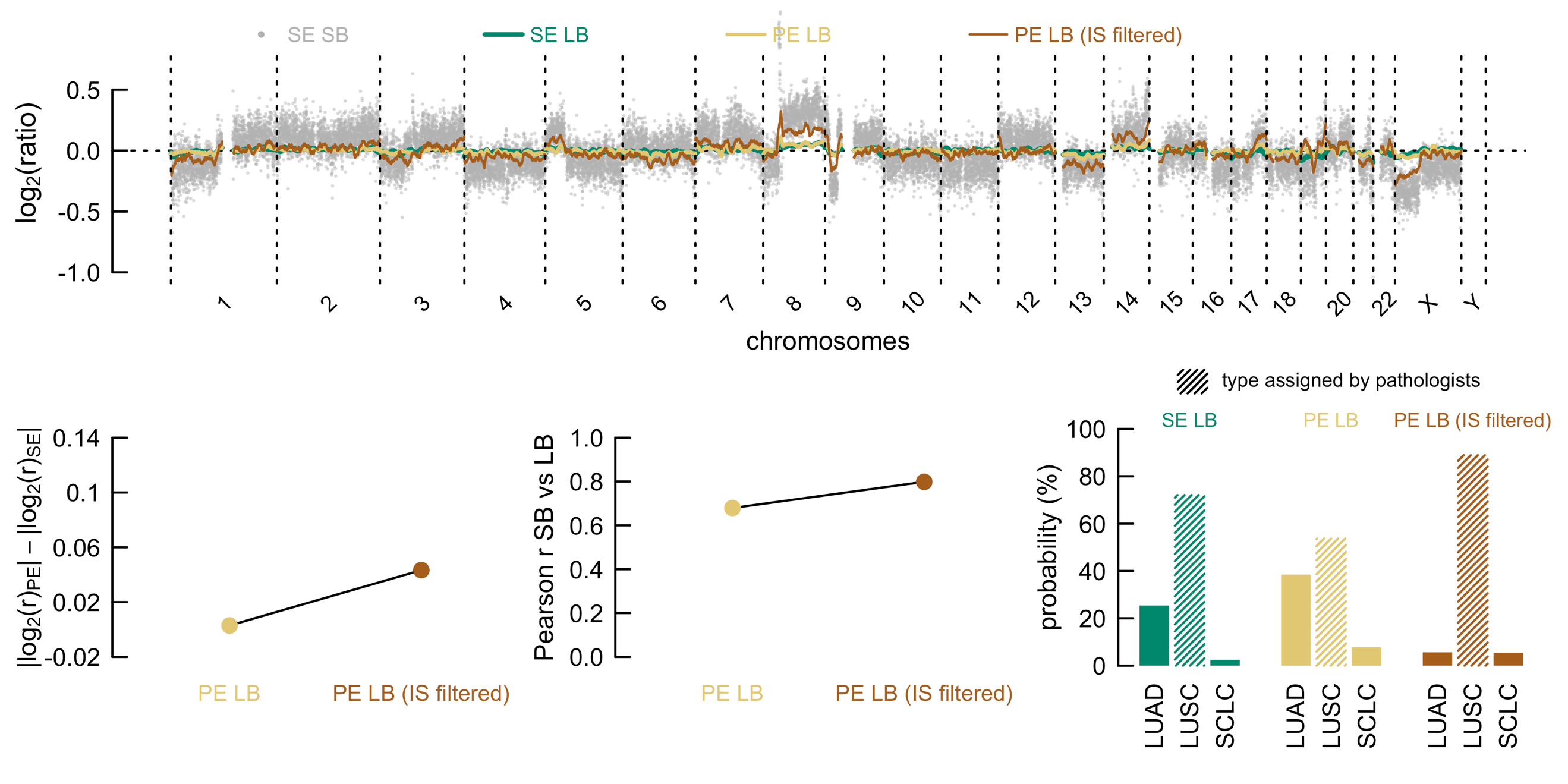


# Copy number profiles of patient 13


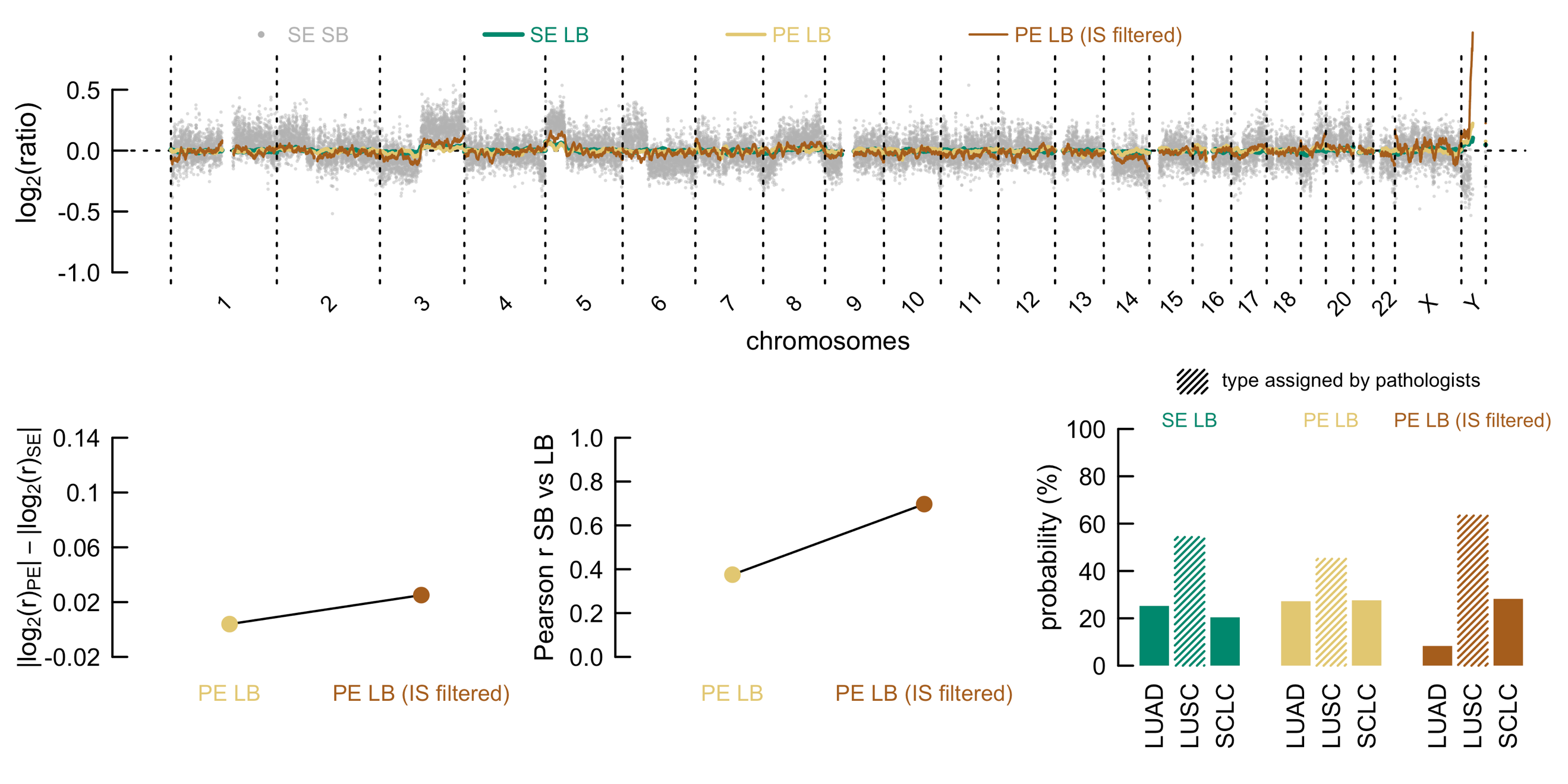


# Copy number profiles of patient 14


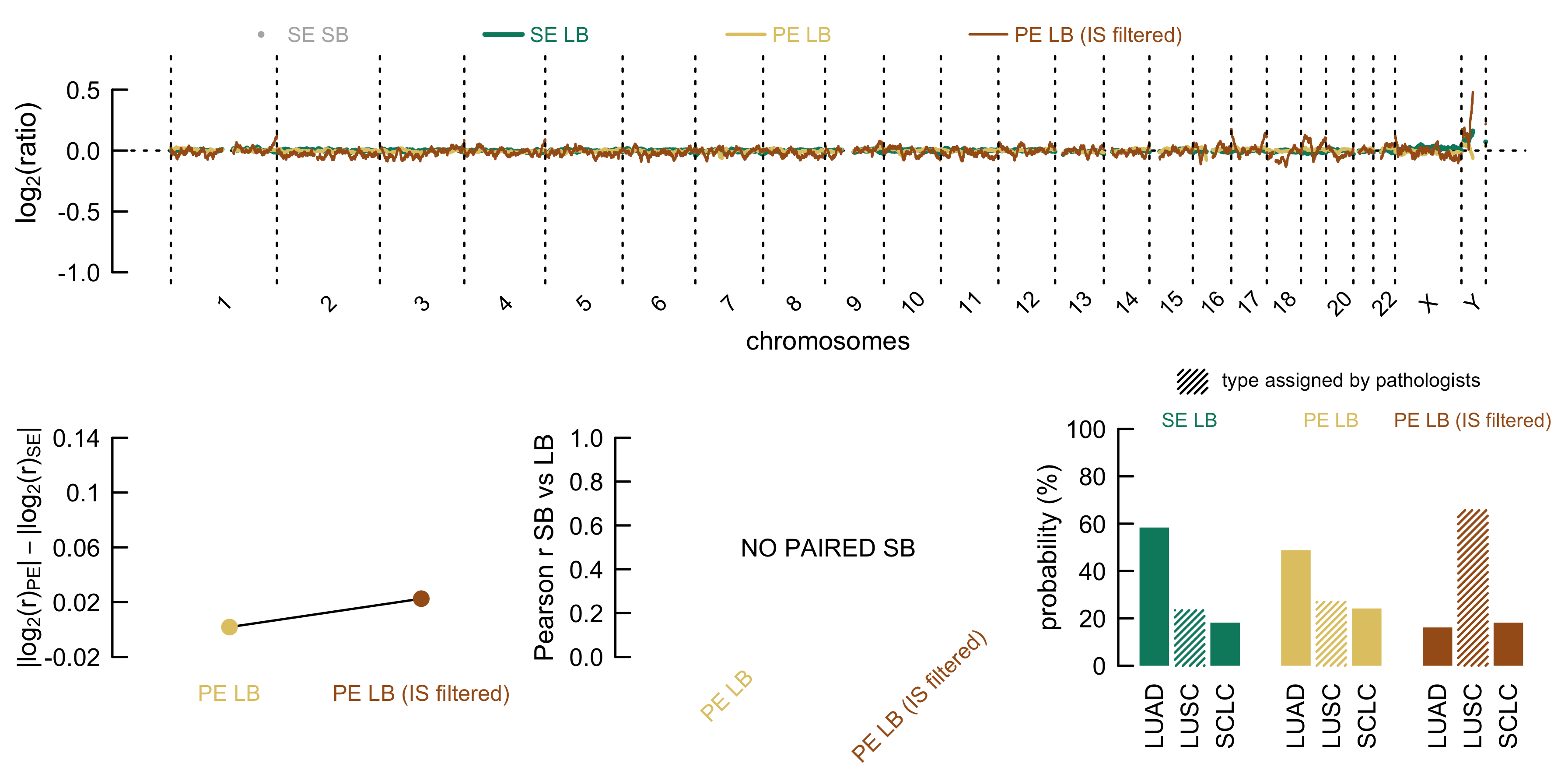


# Copy number profiles of patient 15


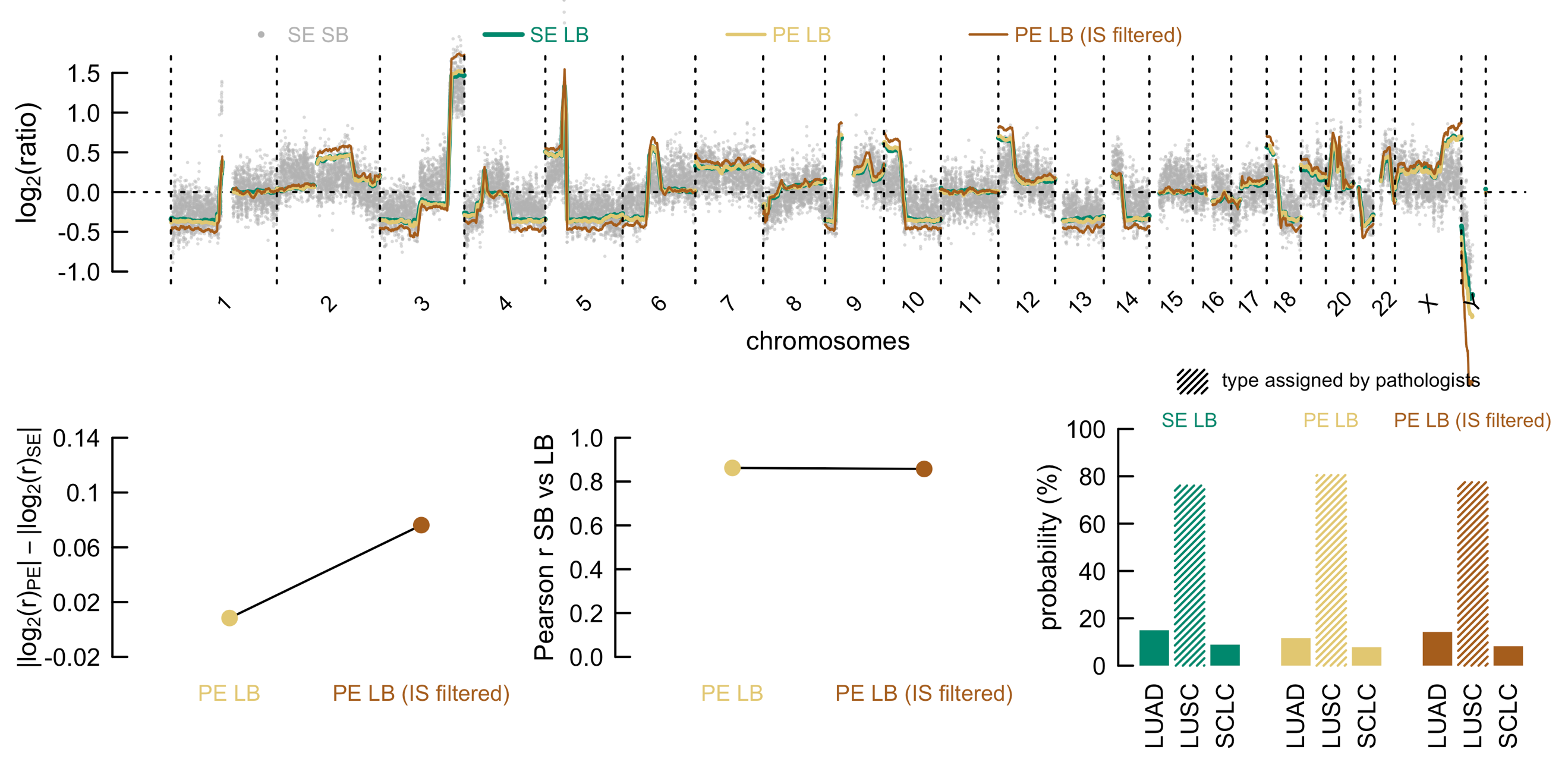


# Copy number profiles of patient 17


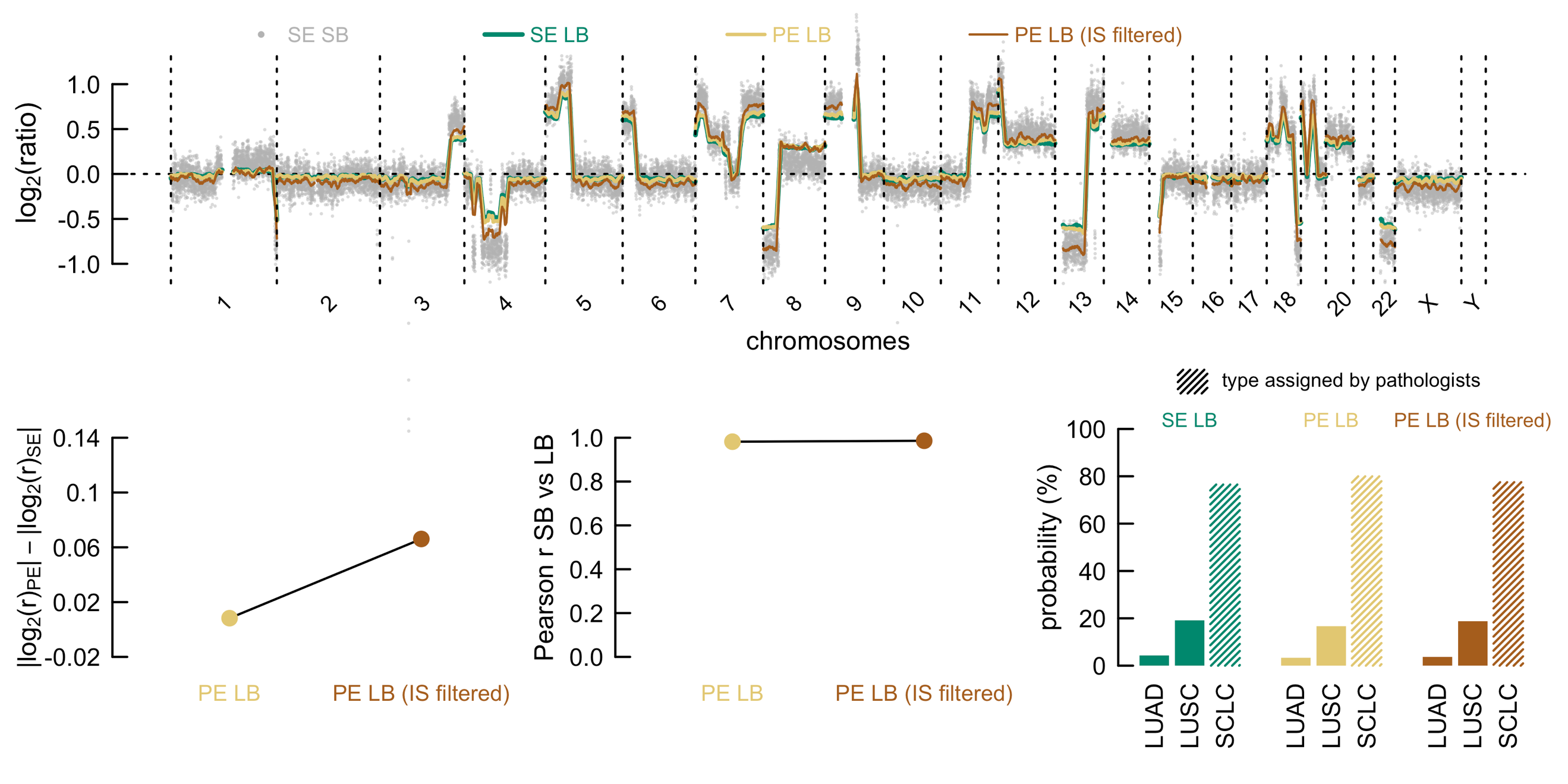


# Copy number profiles of patient 18


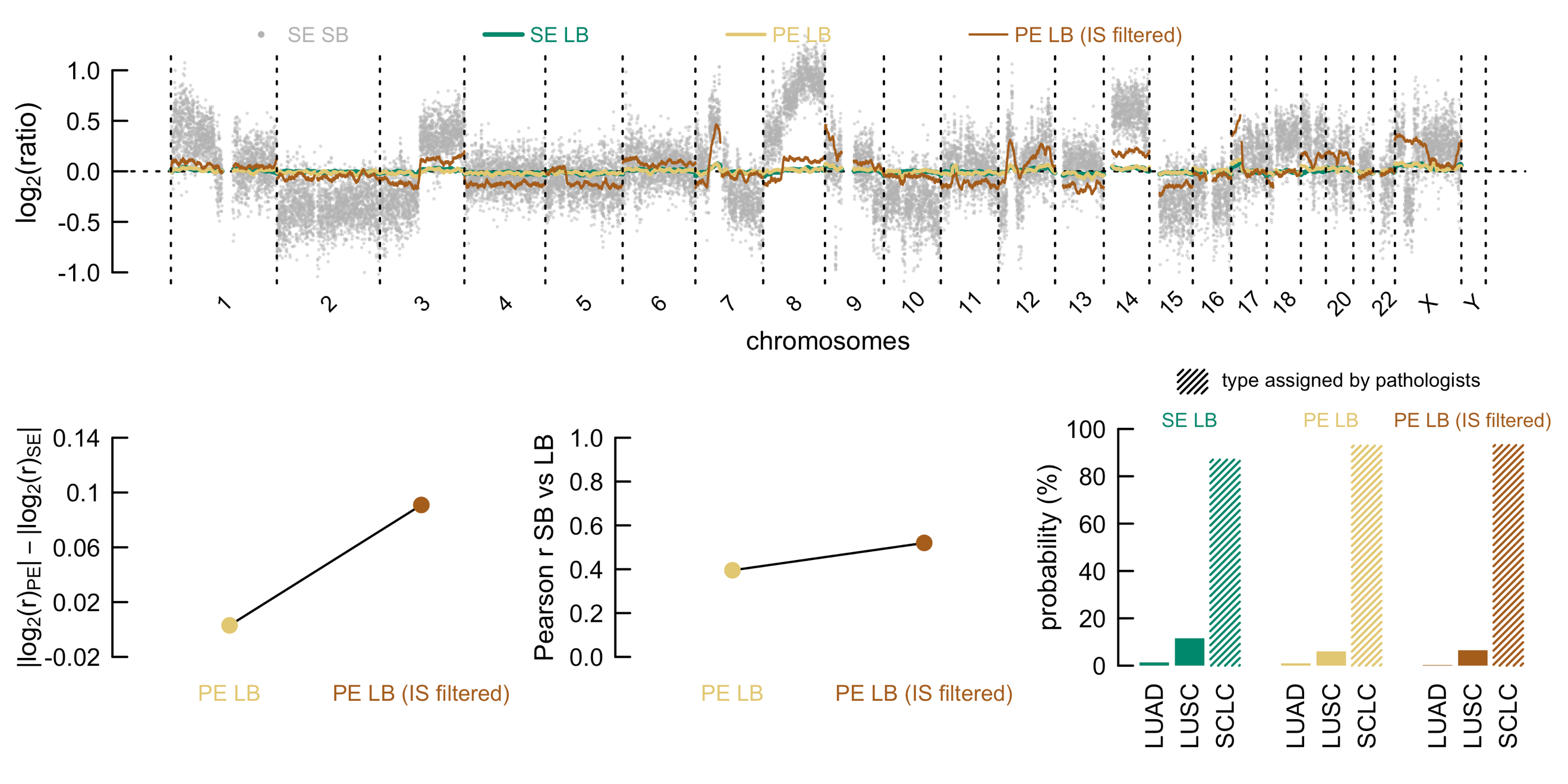


# Copy number profiles of patient 20


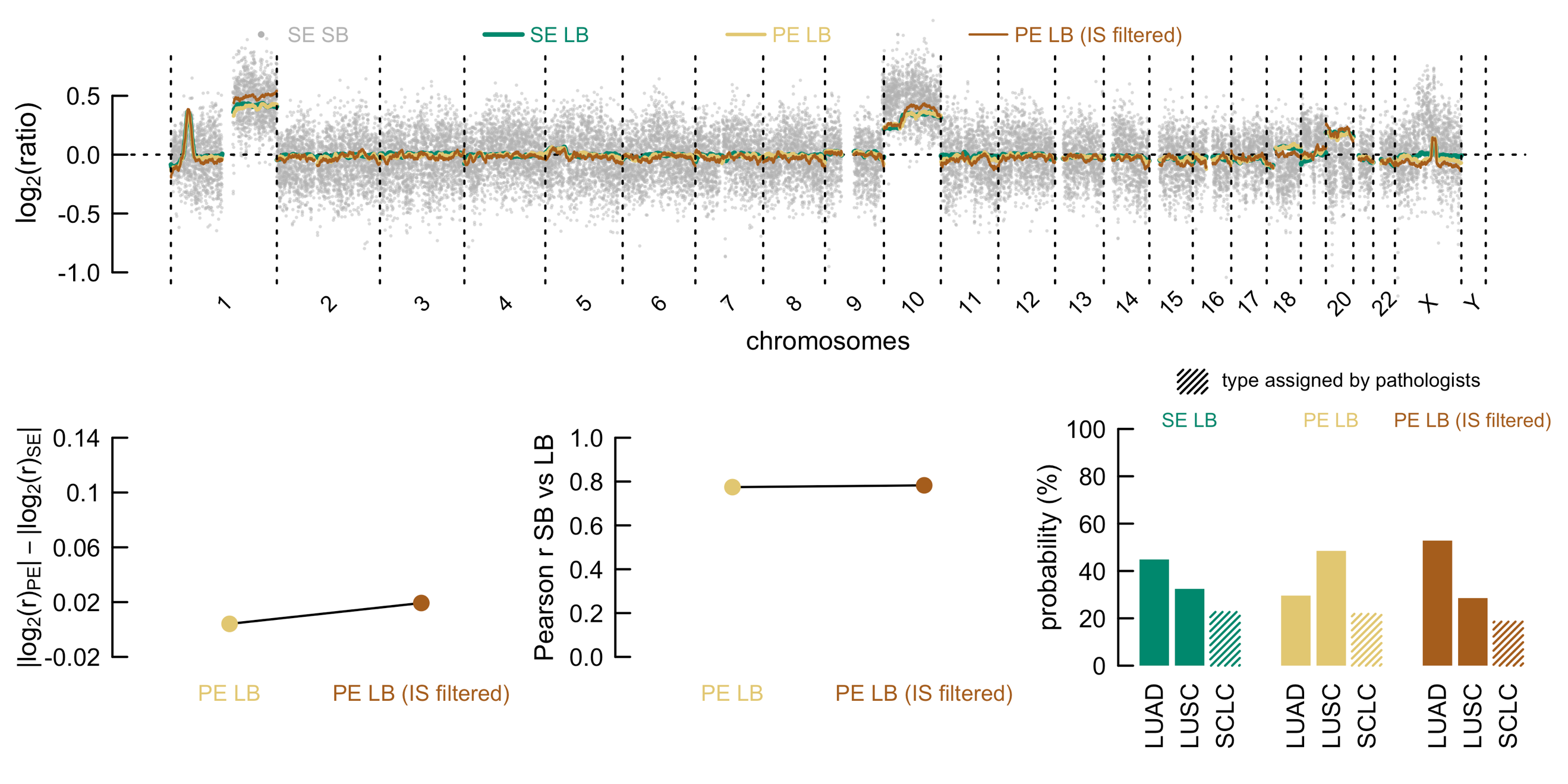


# Copy number profiles of patient 27


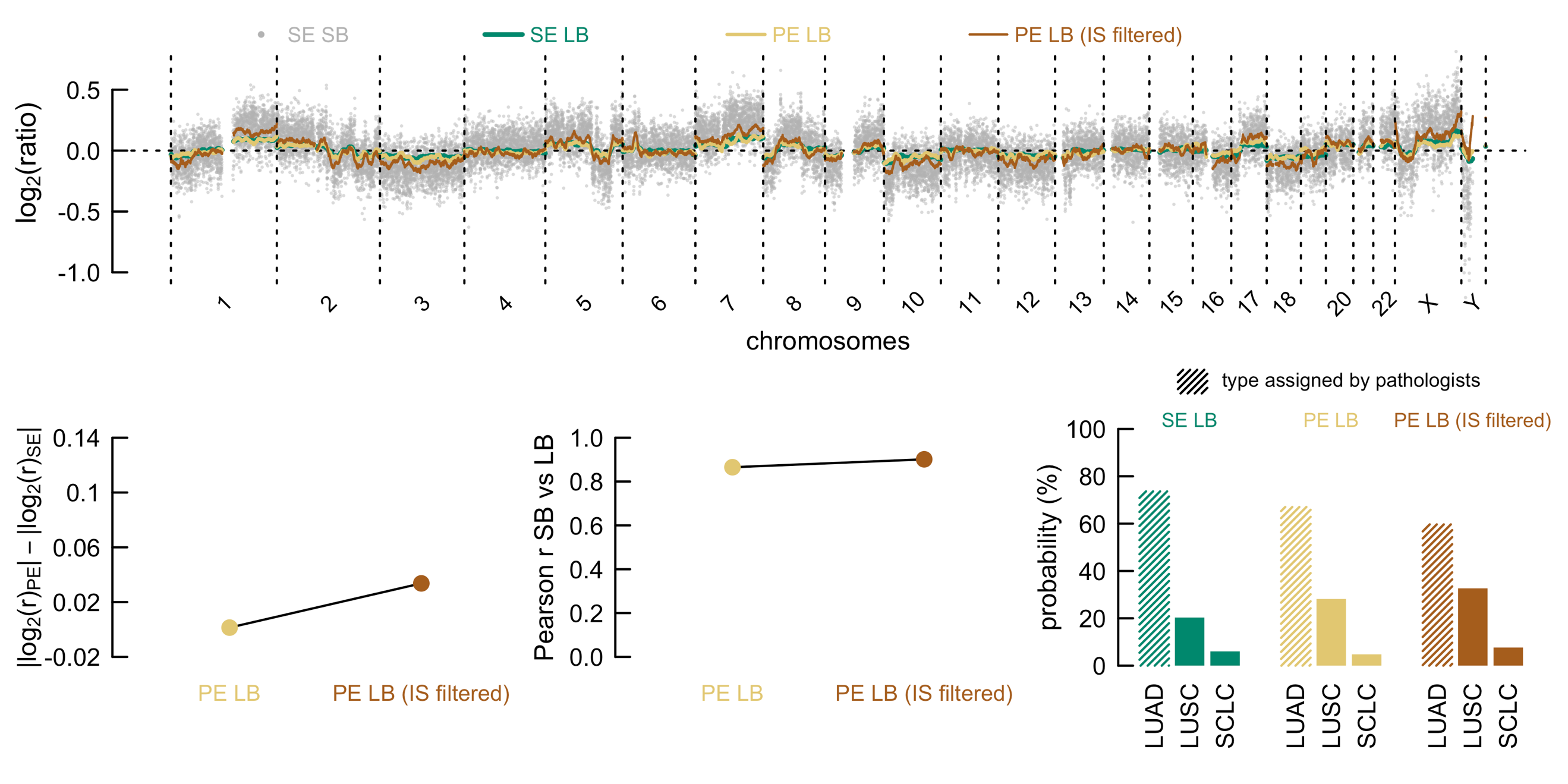


# Copy number profiles of patient 30


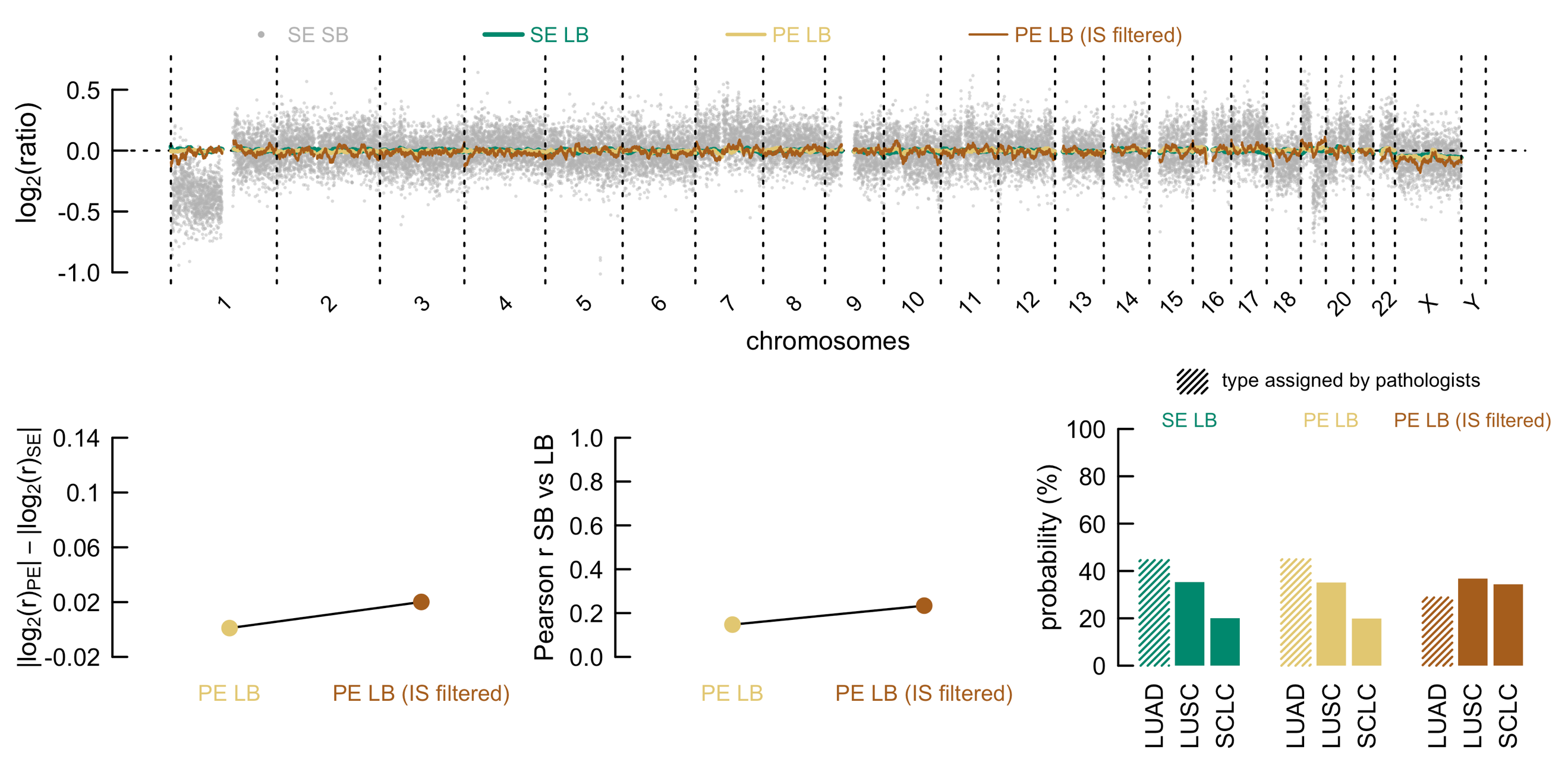


# Copy number profiles of patient 32


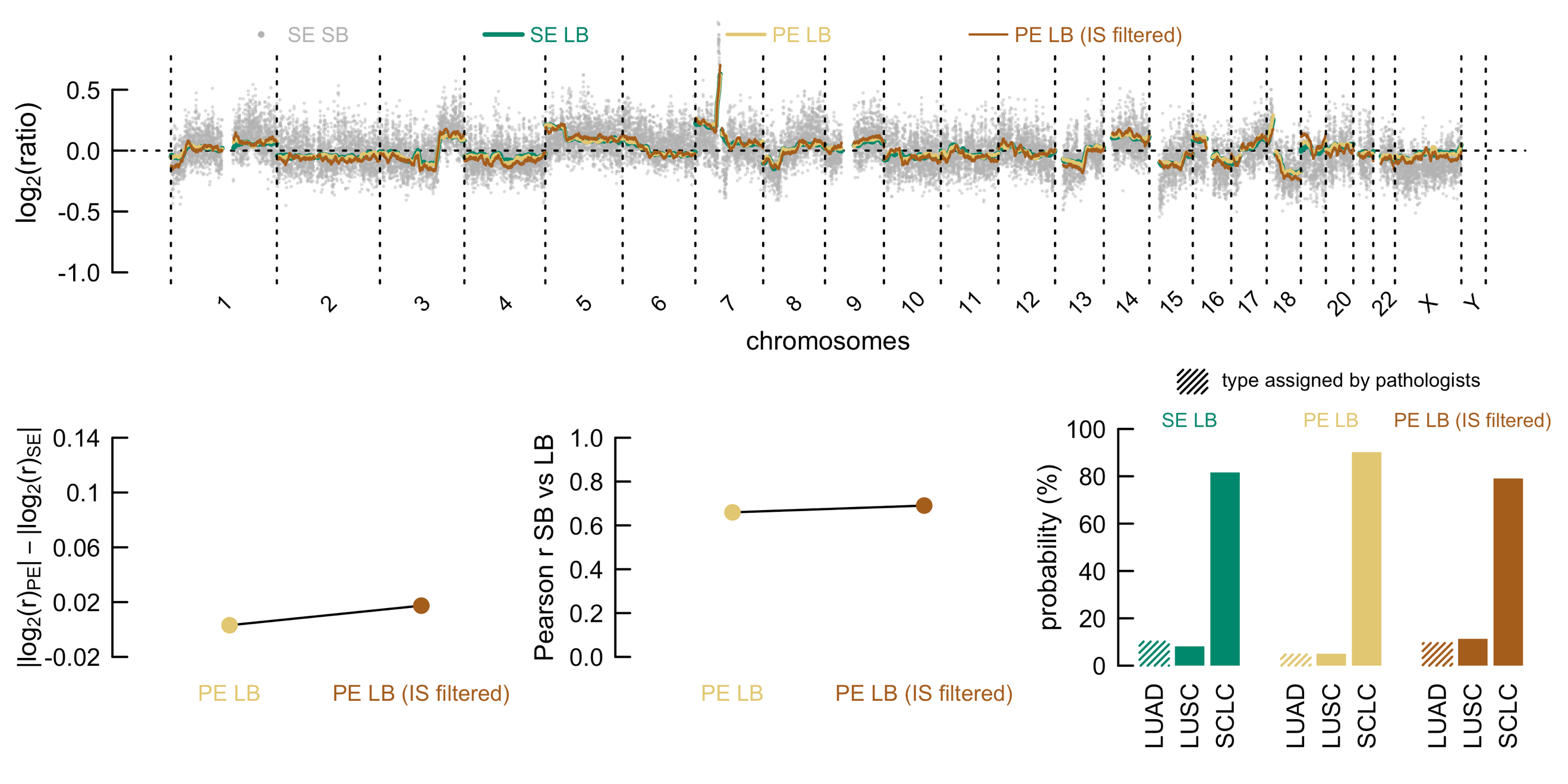


# Copy number profiles of patient 33


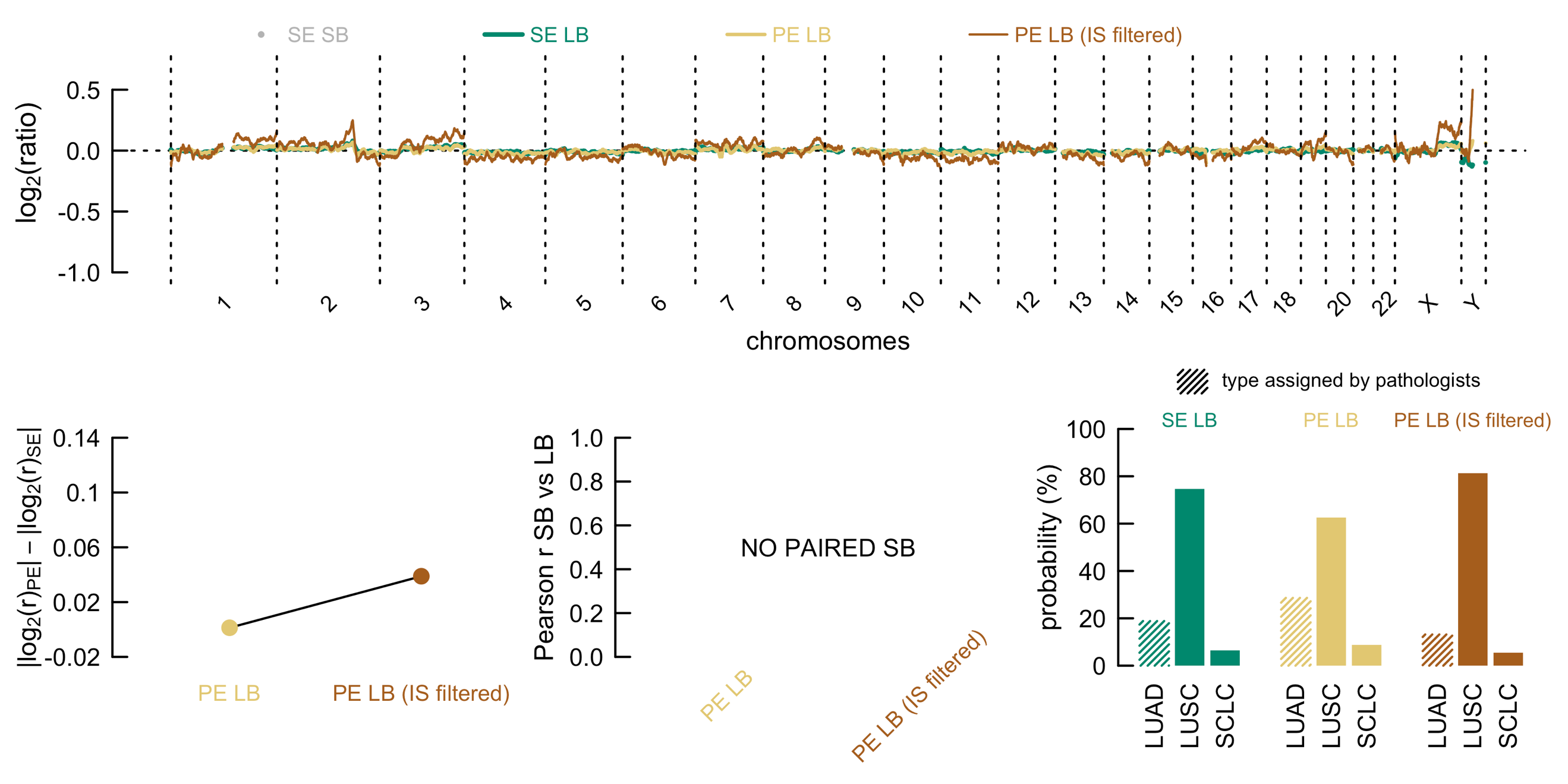


# Copy number profiles of patient 34


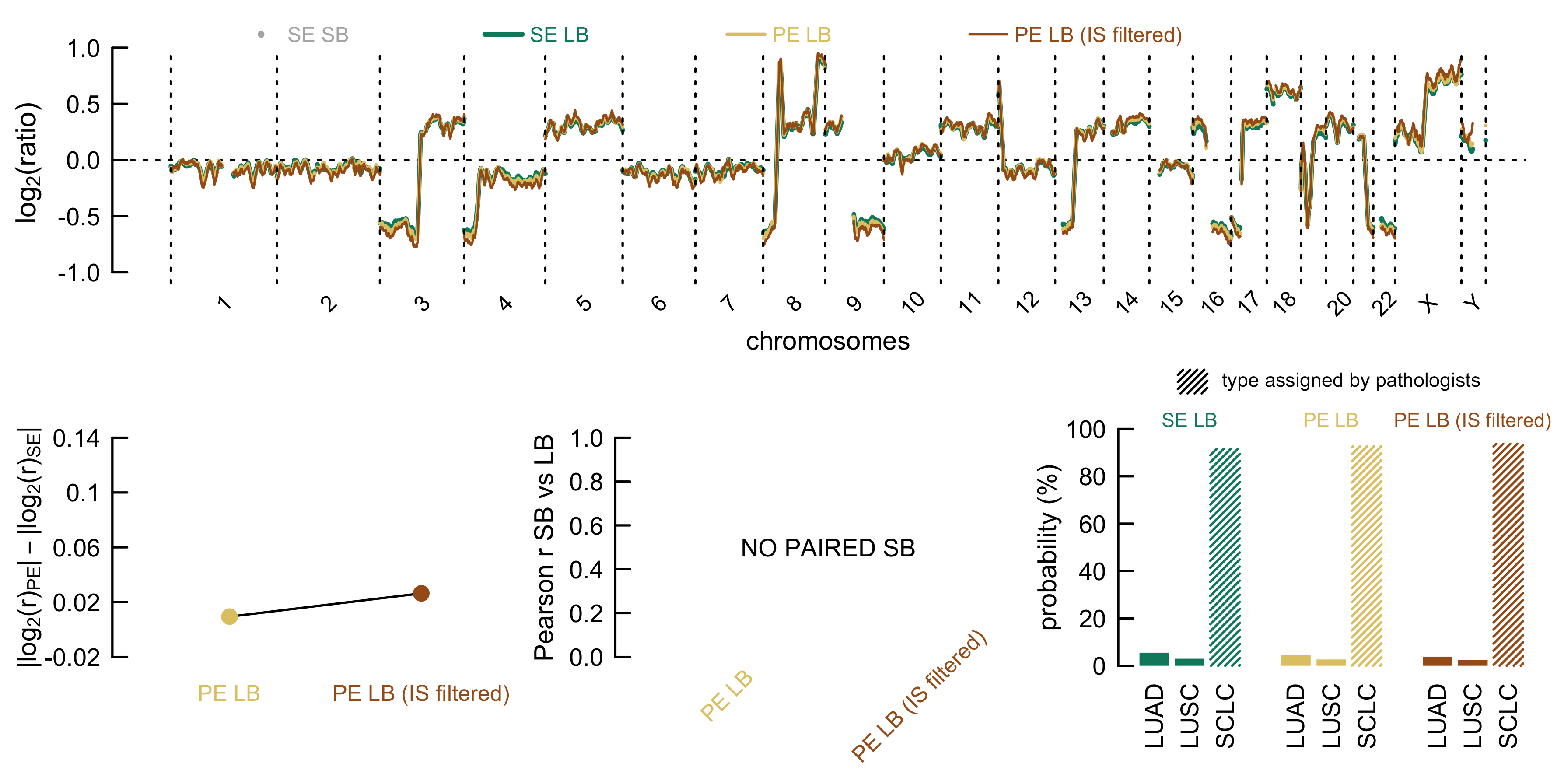


# Copy number profiles of patient 35


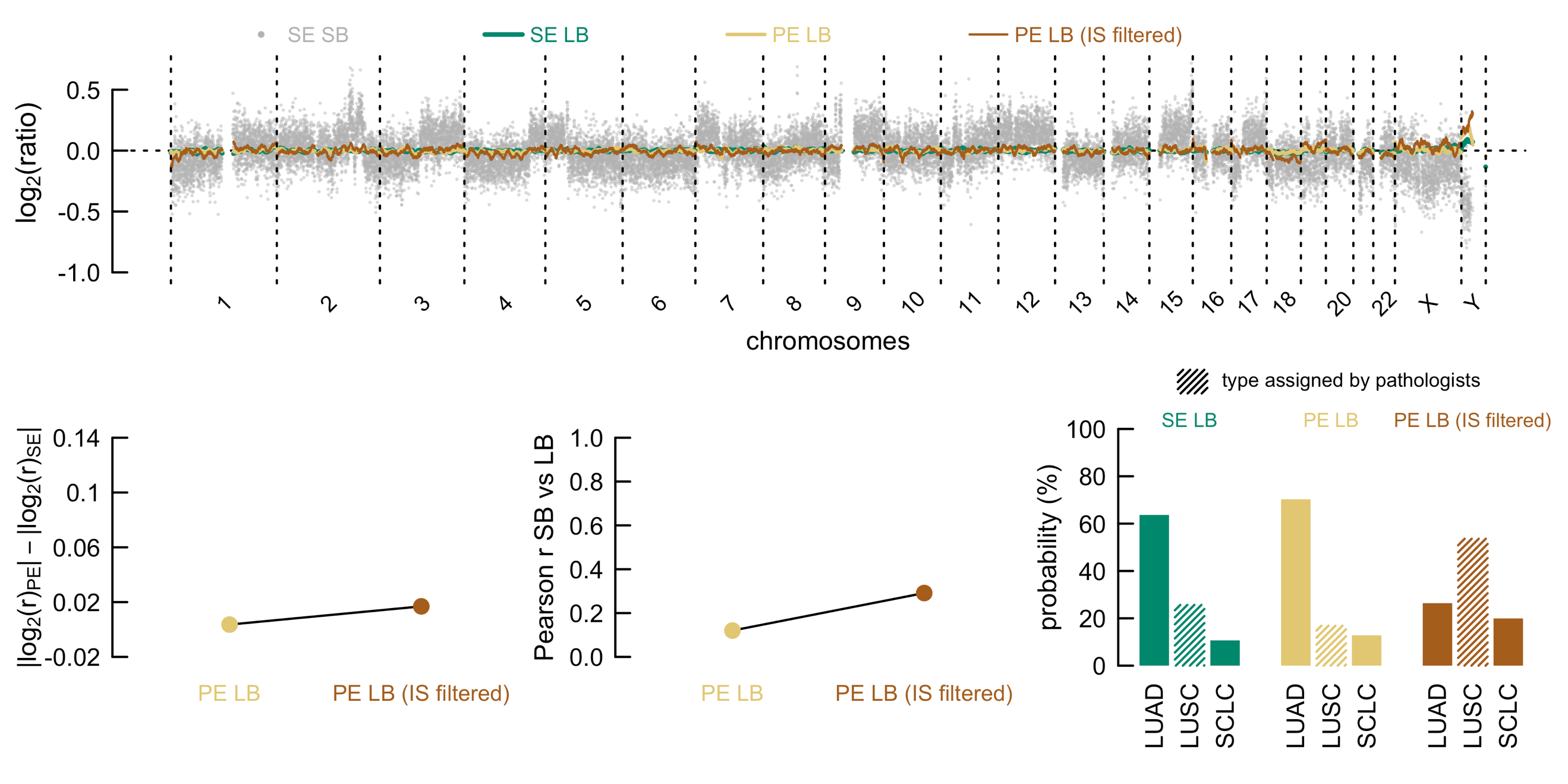


# Copy number profiles of patient 36


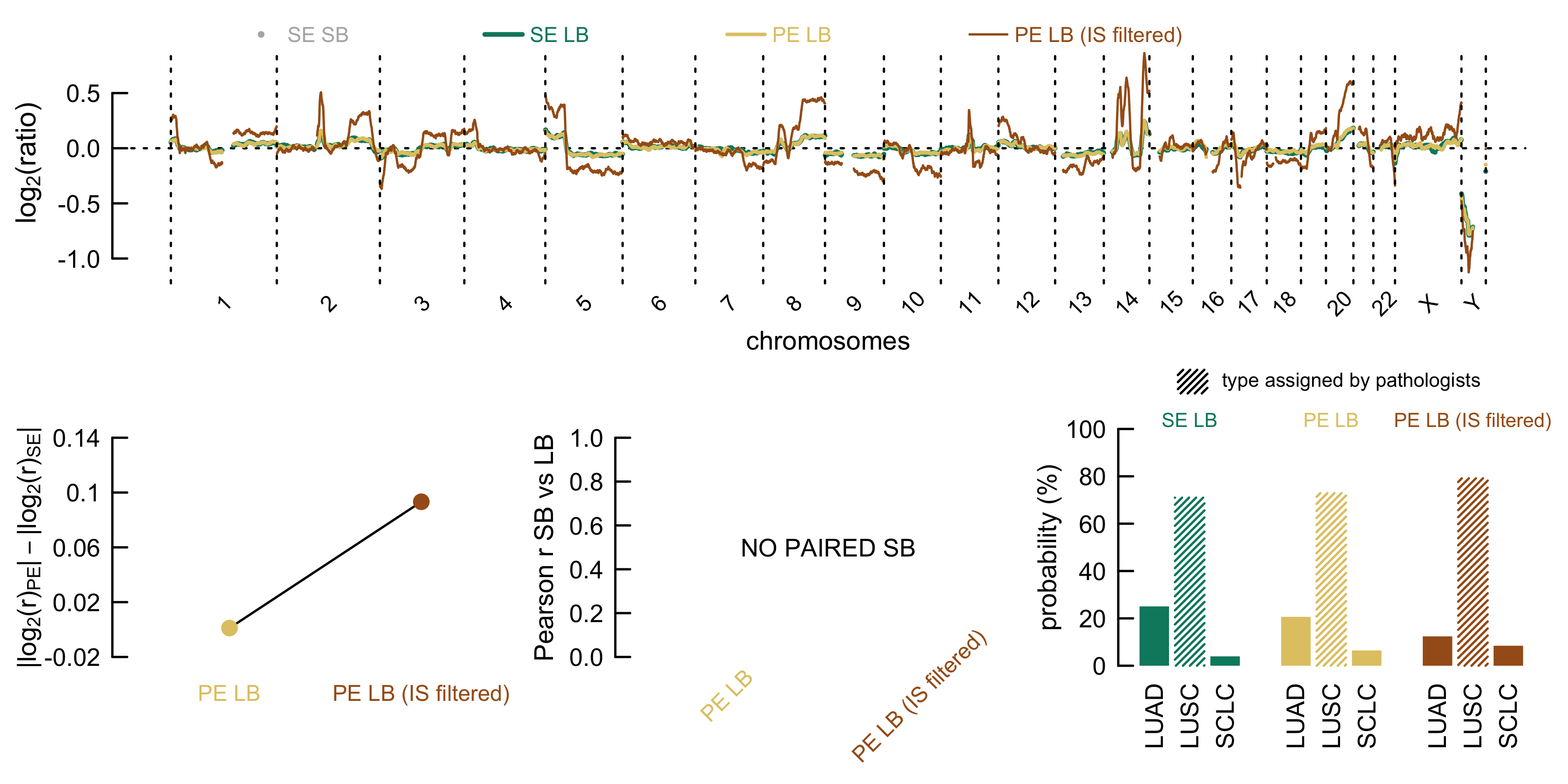


# Copy number profiles of patient 37


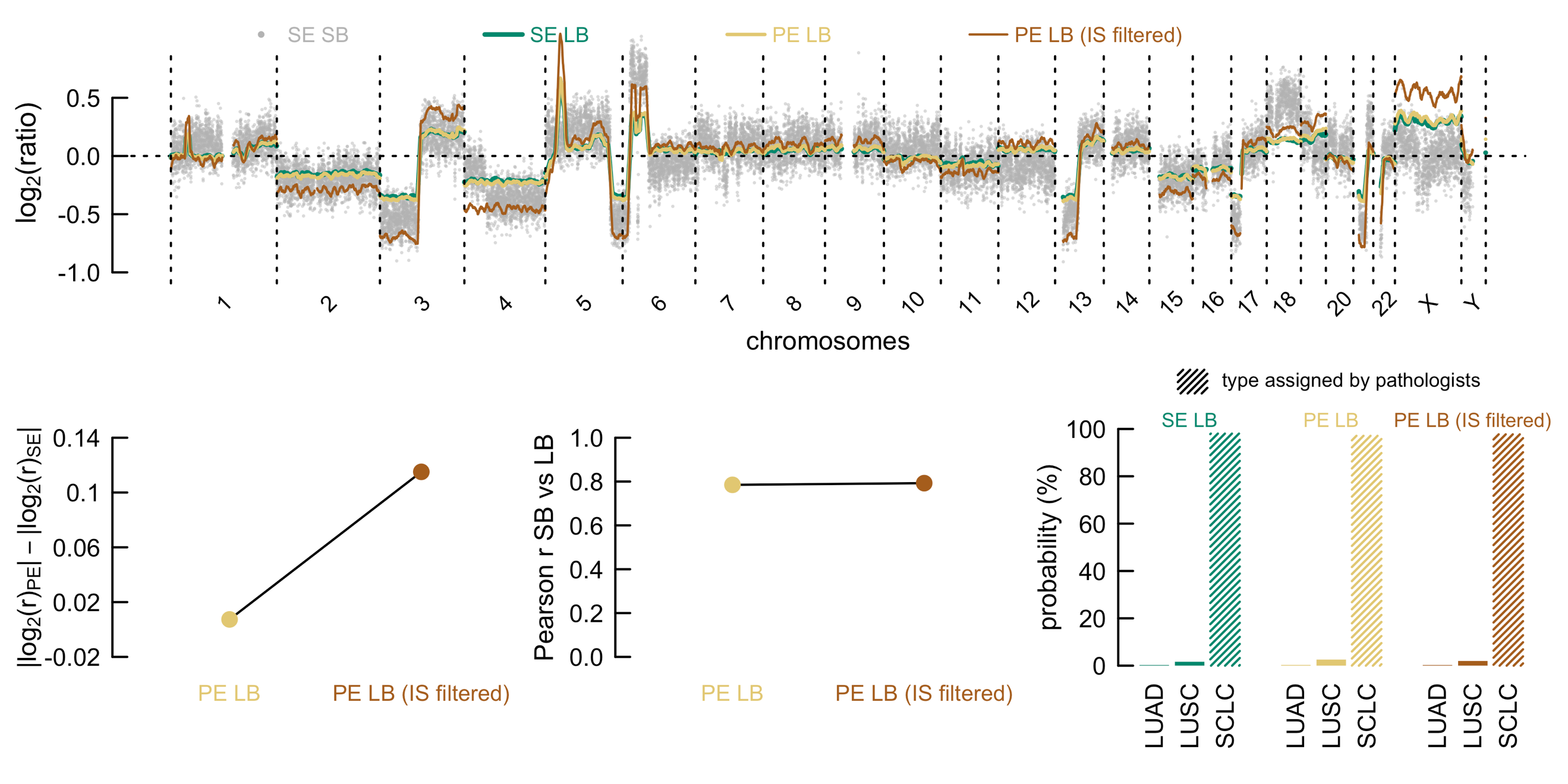


# Copy number profiles of patient 38


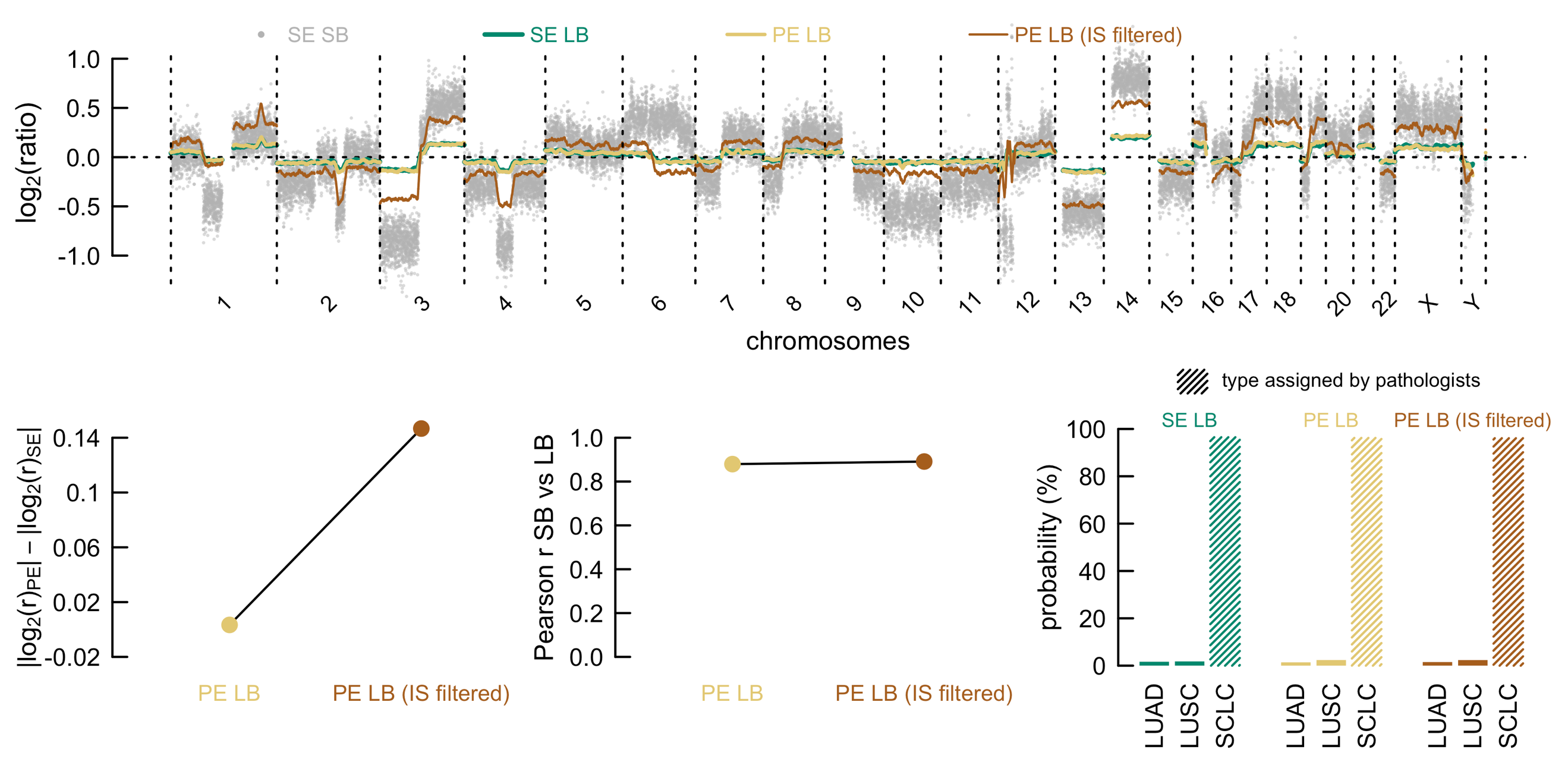


# Copy number profiles of patient 39


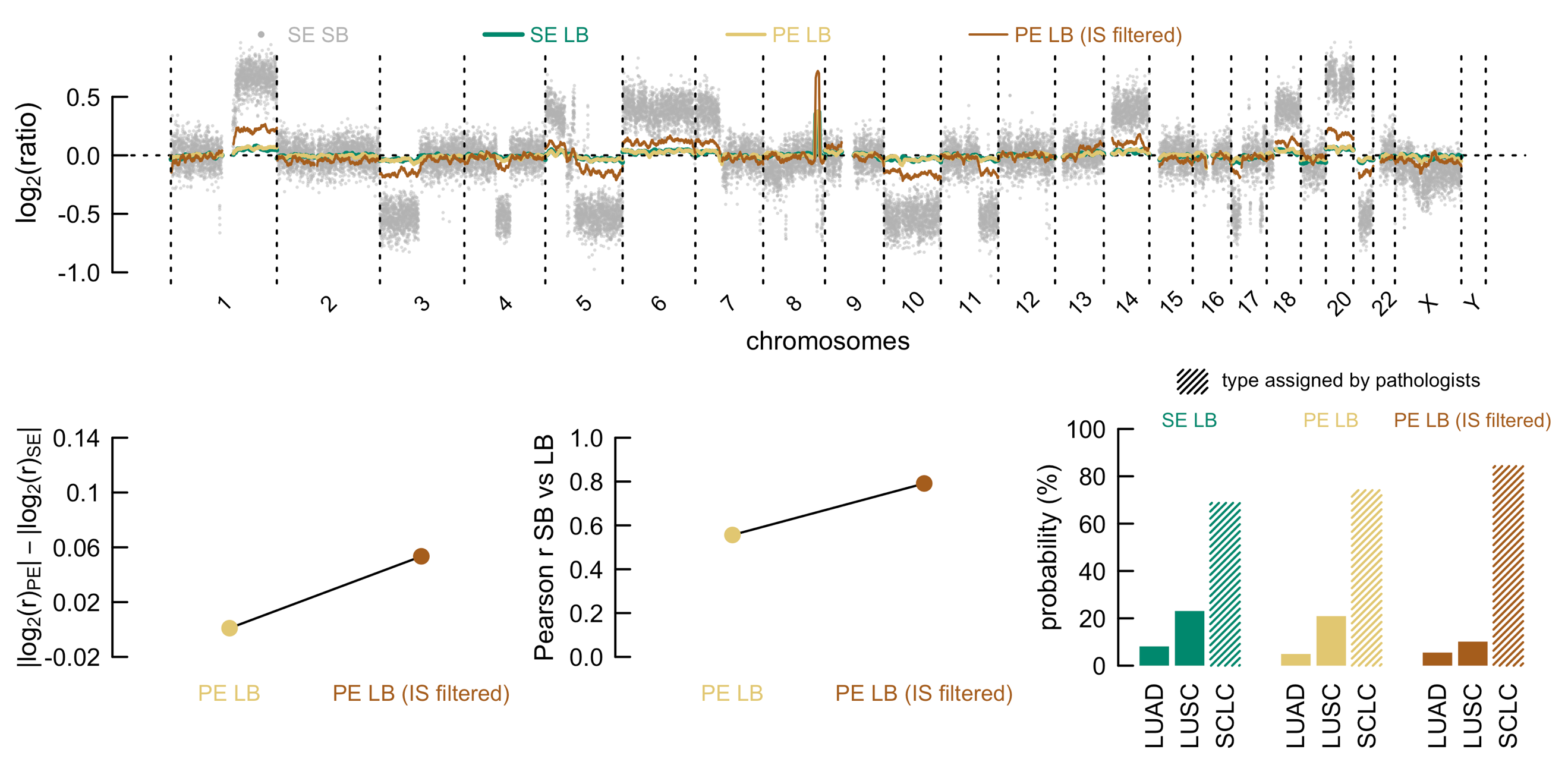


# Copy number profiles of patient 40


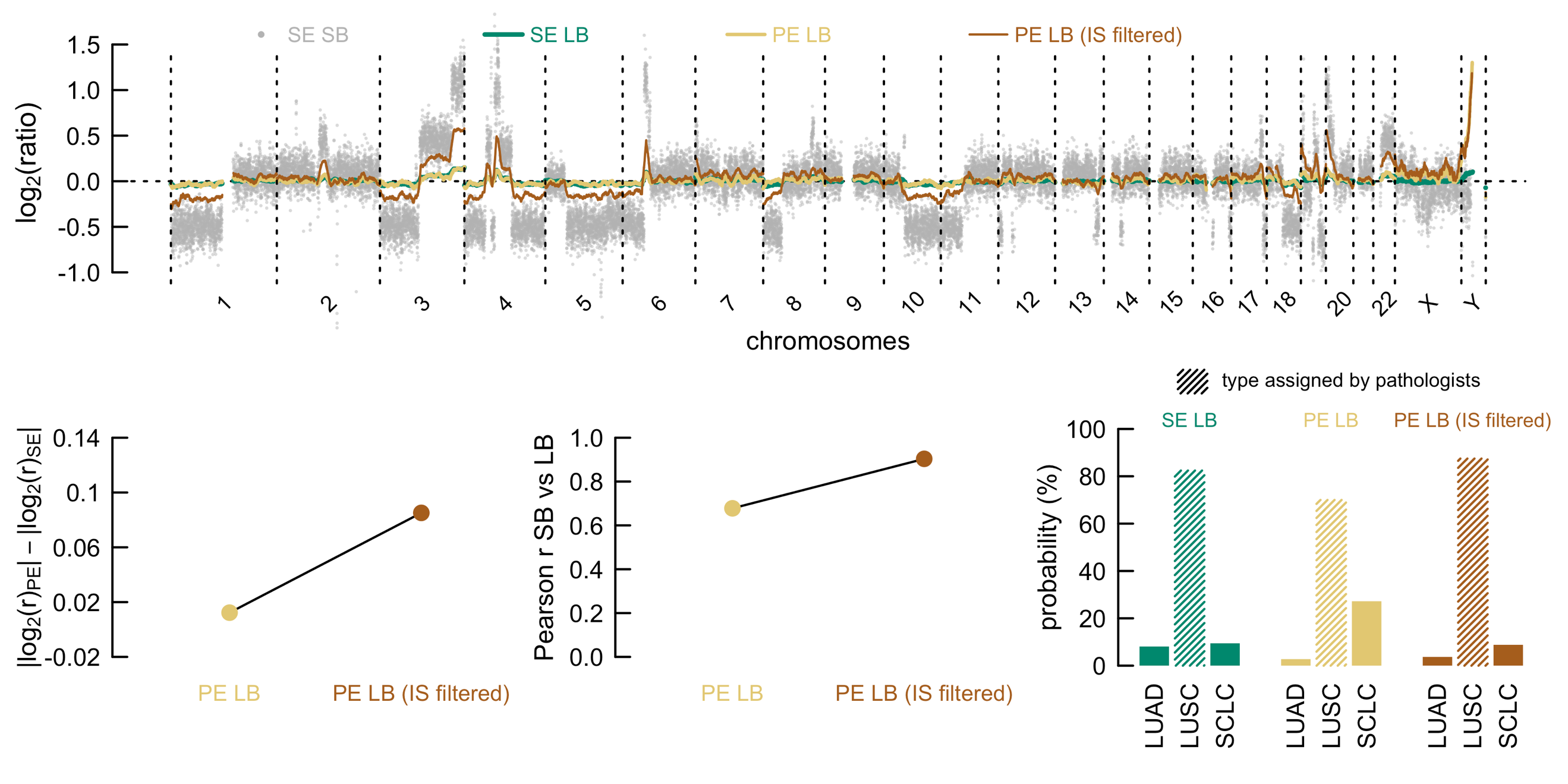


# Copy number profiles of patient 41


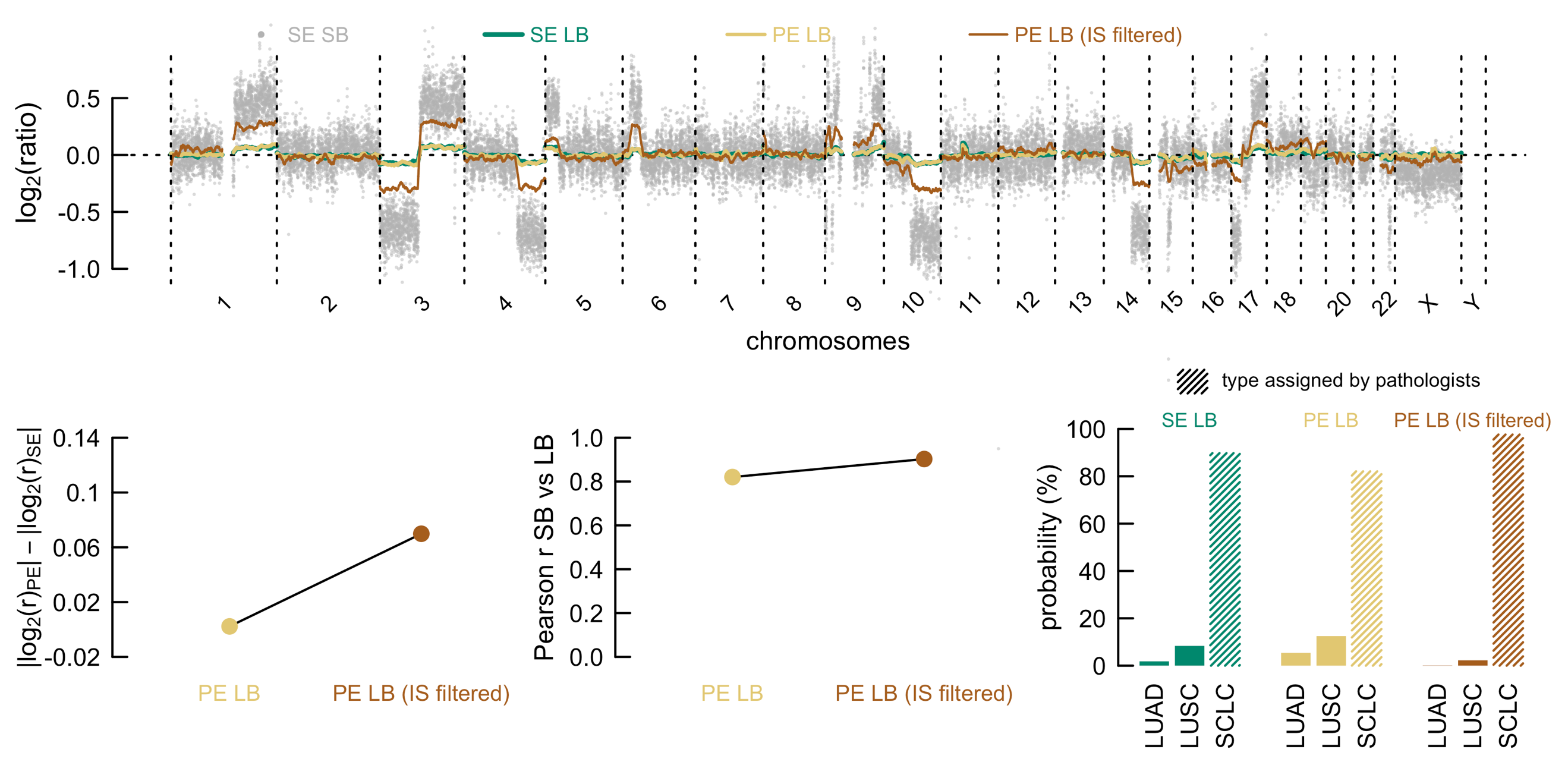


# Copy number profiles of patient 42


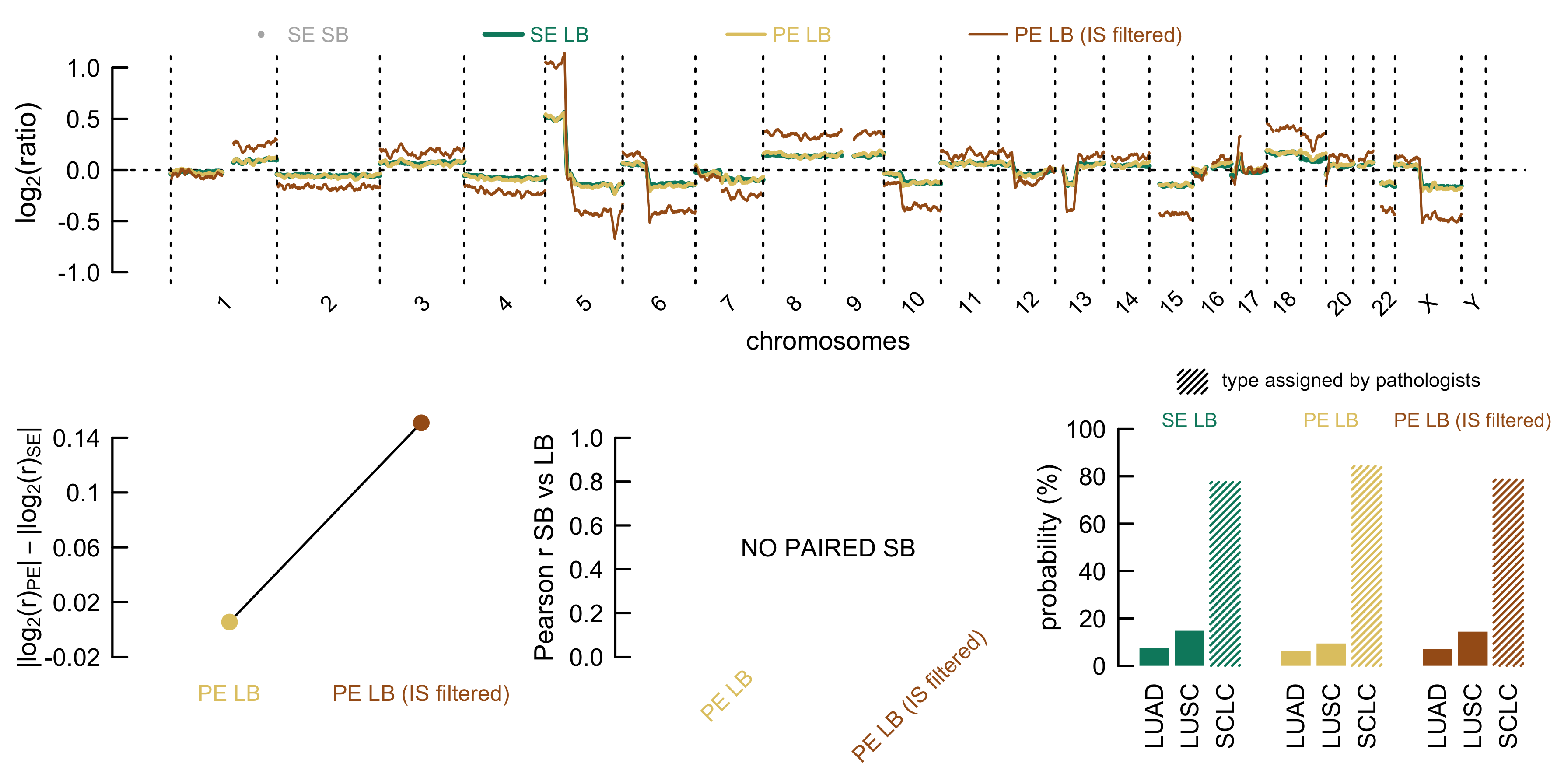


# Copy number profiles of patient 43


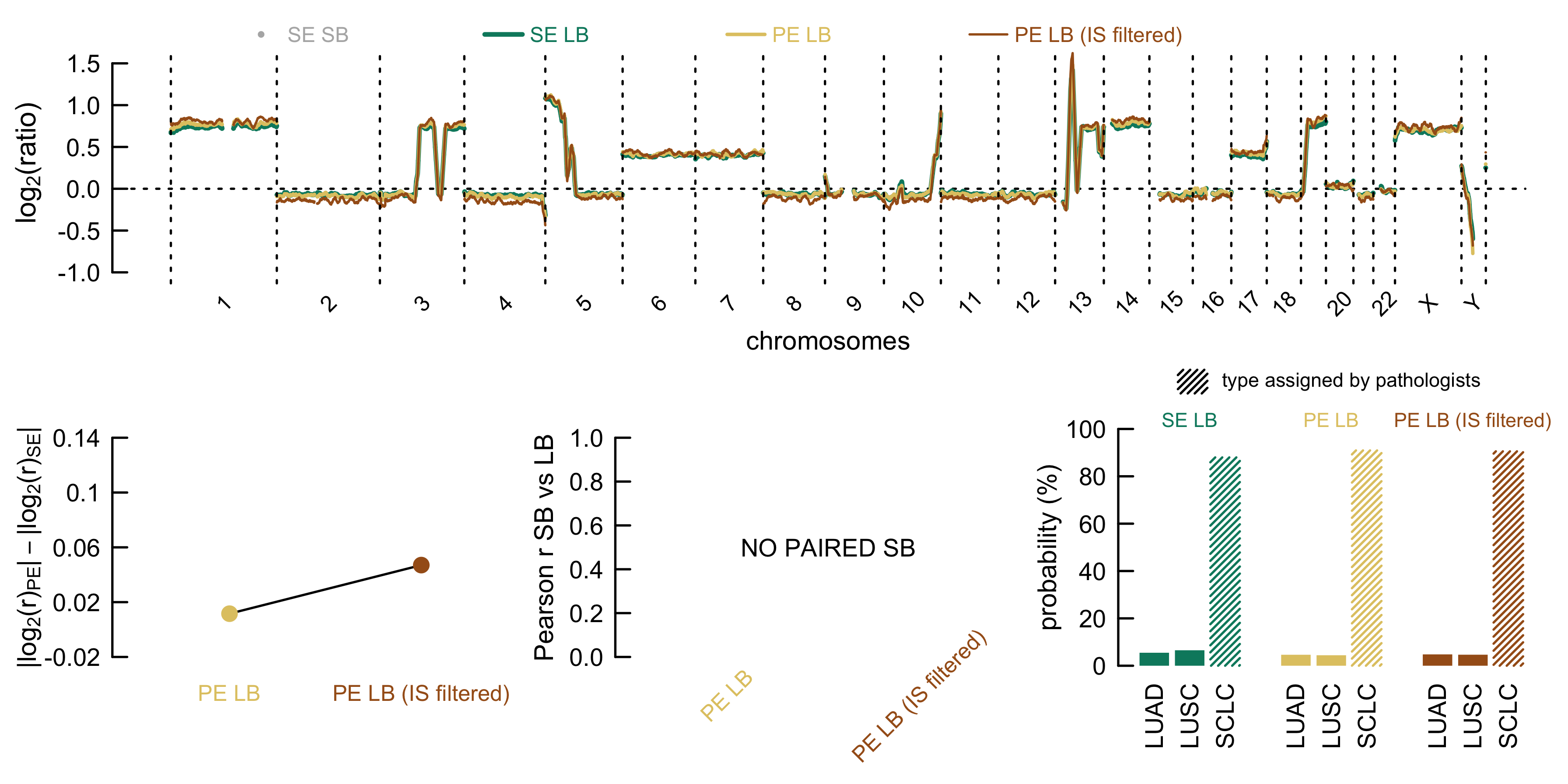


# Copy number profiles of patient 44


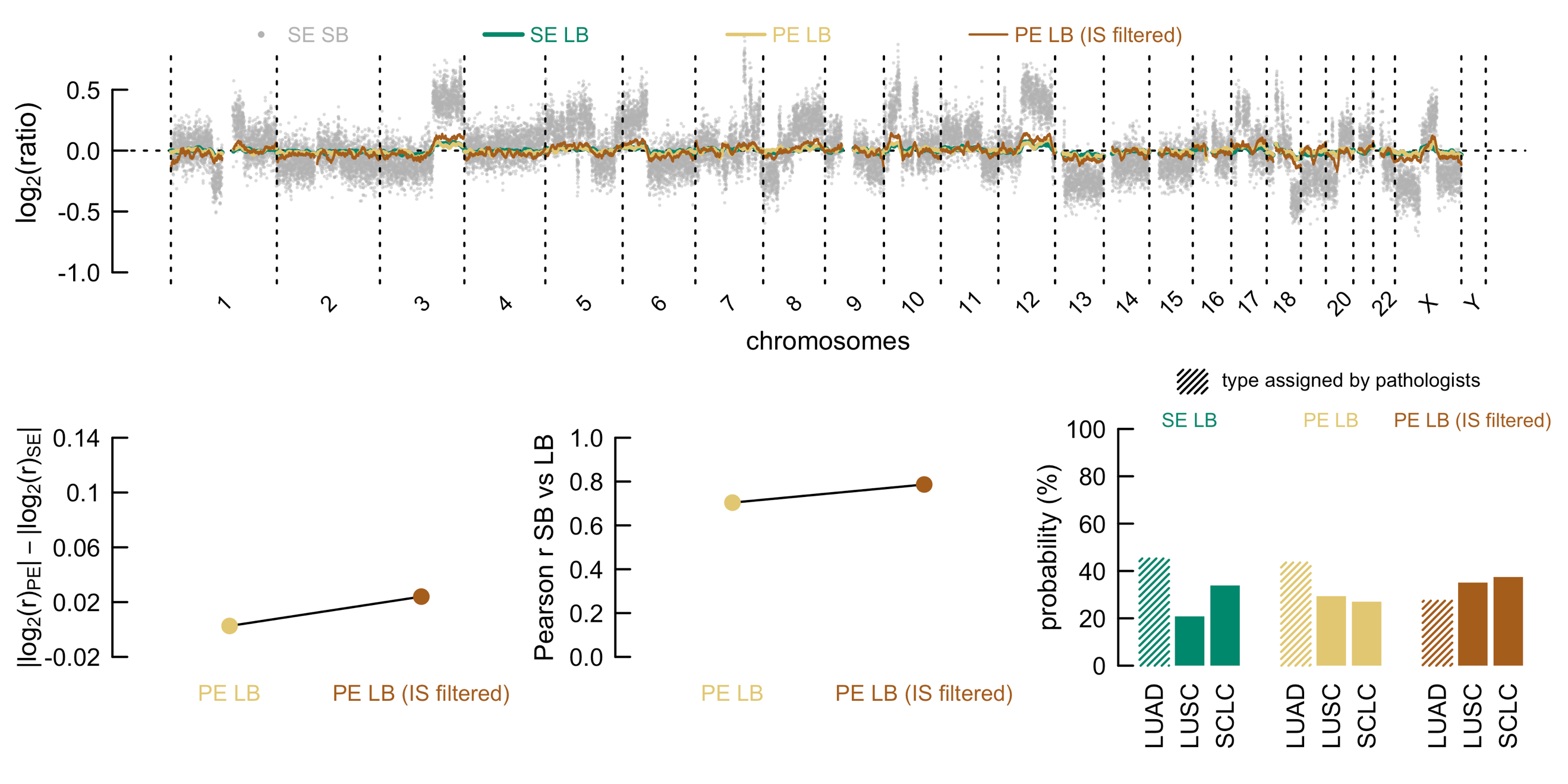

Supplement: Supplementary file 5 — Additional file 5. Supplementary paired-end copy number profiles, containing a thorough patient-wise comparison of paired single/paired-end copy number profiles. Prediction probabilities are shown in addition. [file 13073_2020_735_MOESM5_ESM.docx]
